# Supplementary material for: Genome wide association analysis in a mouse advanced intercross line
Source: Nat Commun. 2018 Dec 4;9:5162. doi: 10.1038/s41467-018-07642-8 (PMC6279738; doi:10.1038/s41467-018-07642-8)
Supplement: Supplementary file 1 — Supplementary Information [file 41467_2018_7642_MOESM1_ESM.pdf]

Supplementary Information

Genome wide association analysis in a mouse advanced intercross line

Natalia M. Gonzales, Jungkyun Seo, Ana Isabel Hernandez-Cordero, Celine L. St. Pierre, Jennifer S. Gregory, Margaret G. Distler, Mark Abney, Stefan Canzar, Arimantas Lionikas, and Abraham A. Palmer

Supplementary Methods 2
1. Phenotypes 2
1.1. Overview of phenotype data pre-processing 2
1.2 Conditioned place preference (**CPP**) and locomotor behavior 2
1.2.1. CPP background 2
1.2.3. CPP paradigm and testing environment 3
1.2.4. Measurement of CPP and locomotor behavior 3
1.2.5. Analysis of CPP and locomotor behavior 3
1.3. Prepulse inhibition of the acoustic startle response (**PPI**) 4
1.3.1. PPI paradigm and testing environment 4
1.3.2. Measurement of PPI and startle 4
1.3.3. Analysis of PPI and startle 4
1.4. Fasting blood glucose levels 5
1.5. Coat color 5
1.6. Wildness 5
1.7. Tissue collection 6
1.8. Hind limb muscle and bone 6
2. Genotying by sequencing (**GBS**) quality control 6
2.1. Identification of GBS sample mix-ups 6
2.2. Variant calling and quality control for error-corrected data (n=1,063) 7
2.2.1. Genotype likelihoods 7
2.2.2. Imputation 7
2.2.3. Hardy-Weinberg Equilibrium (**HWE**) 8
3. Identification and correction of RNA sample mix-ups 8
4. Genome-wide significance thresholds 9
4.1. Multiple hypothesis testing correction for GWAS 9
4.2. LOD drop intervals 9
4.3. Significance of *trans*-eQTLs 9
5. Software and URLs 10
5.1. R packages 10
5.2. Other software 10
5.3 URLs 10
Supplementary Table 1 11
Supplementary Note 1 12
Supplementary References 22
Supplementary Figures 1-24 25

Supplementary Methods

Here we have provided details about the animals and phenotypes measured in this study in accordance with ARRIVE Guidelines. All mouse experiments were approved by the University of Chicago IACUC (https://iacuc.uchicago.edu/). We describe how phenotypes were collected and analyzed in extended detail and we describe how we identified and corrected sample mix-ups in GBS and RNA-seq data. We also provide background about genome-wide significance thresholds and our rationale for using 1.5-LOD intervals to define associated loci. Finally, we list software and online resources that we used in our analyses.

1. Phenotypes

1.1. Overview of phenotype data pre-processing

We measured phenotypes in 1,123 AIL mice (562 female, 561 male) (Aap: LG,SM-G50-56) but only processed phenotype data for 1,063 mice (530 female, 533 male) that had high-quality GBS data. Sex was included as a covariate in all trait models. Other covariates (e.g. batch, generation, coat color, testing chamber) were selected in three stages: (1) we used the adjusted r-squared statistic from a univariate linear regression model to estimate the percent of phenotypic variance explained by each variable. (2) Variables that explained 1% or more of the trait variance were used in the model selection R package, leaps, to identify an optimal set of predictors for each trait. (3) We then reviewed each list of selected covariates and made revisions if necessary (trait-specific details are provided below). Residuals from the final trait models were plotted to identify outliers. An individual was considered an outlier if its residual value was more than three standard deviations from the mean and fell outside the 99% confidence interval of the normal distribution. Traits were quantile-normalized after removing outliers. The final sample size for each trait is shown in **Supplementary Data 2** along with a list of covariates used for GWAS.

1.2 Conditioned place preference (**CPP**) and locomotor behavior

1.2.1. CPP background

CPP is an associative learning paradigm that measures the motivational properties of a drug and the ability to associate its effects with a particular environment [1]. The subjectively positive effects of drugs are thought to contribute to the early stages of drug abuse in humans[2,3]. Humans and rodents display inter-individual variability in response to and preference for drugs of abuse[2,4] and this variability is known to be heritable[5–7]; moreover, the positive subjective response to drugs is correlated with the development of drug abuse[2,2,8]. Accordingly, we and others have proposed that individuals who are genetically predisposed to respond positively to drugs are more likely to continue using them, putting them at greater risk to develop addiction[9,10]. Thus, a broad goal of our approach was to identify genes that predispose an individual to like or dislike drugs of abuse so that future experiments might determine how such genes increase or decrease risk for drug abuse. Importantly, the positive subjective response to drugs can be measured in rodents, humans, and other model organisms[11,12] using the CPP paradigm.

Results from studies of CPP for methamphetamine[4,13,14] and d-amphetamine[15,16] in healthy human subjects demonstrates that self-reported preference for a drug-paired room is correlated with the self-reported pleasant effects of psychostimulants, suggesting that CPP is a reasonable way to measure the hedonic properties of rewarding drugs. However, the relationship between CPP and subjective preference for a drug is ambiguous in model organisms because behavioral observation rather than self-report is used to infer their internal states. Therefore, other aspects of reward, including associative learning, craving, or drug aversion[1,17] should also be considered when interpreting CPP in animals.

1.2.3. CPP paradigm and testing environment

Under the CPP paradigm, mice learn to distinguish between two environments that are paired with either administration of a drug or administration of saline. After repeated pairings, mice are given a choice between the two environments. An increased amount of time spent in the drug-paired environment is interpreted as 'preference' for the drug.

As previously described [18], we created two visually and tactilely distinct environments by dividing a transparent acrylic testing chamber (37.5 × 37.5 x 30 cm; AccuScan Instruments, Columbus, OH, USA) into equally sized arenas using an opaque divider with a passage (5 x 5 cm) in the bottom center. The other three walls of each partition are distinguished by visual cues (stripes on the walls) and tactile cues (floor textures). The chamber is placed inside a frame that transmits an evenly spaced grid of infrared photo beams through the chamber walls. Beam breaks used to monitor the mouse's activity and location are converted to time (s) and distance (cm) units by the AccuScan VersaMax Software (AccuScan Instruments, Columbus, OH, USA). Each testing chamber is encased within a sound attenuating PVC/lexan environmental chamber. Overhead lighting provides low illumination (~80 lux), and a fan provides both ventilation and masking of background noise.

We reversed the divider during conditioning trials to restrict the mouse to one arena within the testing chamber. 1 mg kg^{-1} methamphetamine was always paired with the left arena (white horizontal stripes, smooth floor) and physiological saline was always paired with the right arena (black vertical stripes, textured floor). We had previously established that mice did not prefer one arena over the other[18]. The 1 mg kg^{-1} dose of methamphetamine was intended to generate preference and locomotor stimulation without inducing stereotyped behaviors.

1.2.4. Measurement of CPP and locomotor behavior

CPP and locomotor activity were measured simultaneously. Up to a dozen mice were tested using 12 separate CPP chambers. Median age at the beginning of CPP was 54 days (mean=55.09, range=35-101). On each day (**D**) of the assay, mice were placed on a portable shelf and were transported from the colony to an adjacent testing room. Mice were given 30-45 min to acclimate to the room before being removed from their home cages. Before each 30-min test, mice were weighed and momentarily separated into clean holding cages. After injection and placement into test chambers, mice were free to travel between arenas on D1 and D8 after receiving an intraperitoneal injection of physiological saline. On D2-D5 (conditioning trials) mice were intraperitoneally administered either methamphetamine (1 mg kg^{-1}, D2 and D4) or vehicle (saline, D3 and D5) in a volume of 0.01 ml/g body weight. We also measured locomotor activity (total distance travelled in cm) on each day and recorded the number of times the mouse switched between sides of the chamber on D1 and D8. After each 30-min testing session, we returned mice to their home cages. Test chambers were cleaned with 10% isopropanol between runs. Home cages were returned to the colony at the end of each experiment.

1.2.5. Analysis of CPP and locomotor behavior

We define CPP as the increase in time spent in the methamphetamine-paired arena on D8 compared to D1. To account for the possibility of initial preference for one arena, we also considered preference on the final test day (without regard to preference on D1) as a second outcome measure for CPP. We refer to locomotor activity on day 1 as the locomotor response to a novel environment. The locomotor response to saline is measured on D3 and D5. We also measure activity on D8; locomotor activity on both D1 and D8 are unique in that the environment is different because the mouse has access to the entire CPP chamber. Side changes measured on D1 and D8 provided additional measures of locomotor activity in response to novelty and saline, respectively. The locomotor response to 1 mg kg^{-1} methamphetamine is measured on D2 and D4. We also calculated the increase in methamphetamine-induced activity on D4 relative to D2 as a measure of locomotor sensitization. Locomotor sensitization is an increase in the magnitude of drug-induced activity after repeated administration of the same (or subthreshold) dose of the drug [19].

Because behavior is a dynamic response to the environment, we treated each day's measurements as a different set of traits. For example, a mouse's preference for an environment may change after associating it with a rewarding (or aversive) drug experience, and a drug-naive mouse may

have a different response to methamphetamine than a mouse that has already experienced its effects[20]. All CPP and locomotor phenotypes were measured in 5-min bins over the course of 30 min. We summed measurements across all six 5-min time bins to obtain total activity for each day and the total number of side changes side changes for D1 and D8. Thus, we obtained seven individual measurements: the total, and the six individual 5-min time bins. As shown in **Supplementary Fig. 3**, binned measurements are highly correlated; therefore, we used the same set of covariates for all binned phenotypes within a given day and phenotype class.

Occasional software malfunctions that occurred at the time of testing (in which the Accuscan software was unable to record movement in certain chambers for up to 14 s during the test) were automatically detected and included in the output for each 5-min time interval in which the error occurred. Data for these specific intervals and total activity on the affected day were marked as missing in 61 mice. Data were quantile-normalized prior to GWAS.

1.3. Prepulse inhibition of the acoustic startle response (**PPI**)

1.3.1. PPI paradigm and testing environment

When an animal is startled by a loud noise, its skeletal muscles contract rapidly. Although the startle reflex is automatic, it can be inhibited to some degree by a preceding stimulus of lower intensity; this phenomenon is called prepulse inhibition, or PPI [21]. In humans, PPI is measured from the startle-blink reflex, whereas in rodents, muscle contractions are measured from the whole body[22–24]. However, in both experiments, the startle reflex is elicited by a loud tone, and a transducer is used to output a voltage proportional to the magnitude of the animal's response. PPI is also considered to be an endophenotype for various psychiatric conditions, most notably schizophrenia [22,25].

To measure PPI, each mouse is placed inside a 5-cm Plexiglas cylinder within a lit, ventilated testing chamber (San Diego Instruments, San Diego, CA, USA). The mouse's movements are detected by a piezoelectric sensor. Once inside the chamber, mice have five min to acclimate to 70 dB white noise, which remains in the background for the duration of the 18-min test. After acclimation, mice are repeatedly exposed to acoustic startle stimuli (120 dB pulse; 40 ms) which is sometimes preceded by a 20 ms prepulse (3-12 dB above background noise) at variable intervals.

The acclimation period is followed by 62 trials that are a mixture of the following five types: a 'pulse alone trial', which consists of a 40-ms 120 dB burst (startle stimulus), a 'no stimulus' trial where no stimulus is presented, and three prepulse trials containing a 20 ms prepulse that is either 3, 6 or 12 dB above the 70-dB background noise level followed 100 ms later by a 40 ms 120 dB pulse. Trials are split into four consecutive blocks. Blocks 1 and 4 each contain 6 pulse-alone trials. Blocks 2 and 3 are a mixture of 25 trials (6 pulse-alone, 4 no stimulus and 15 prepulse trials). The variable intertrial interval is 9–20 s (mean=15 s) throughout all 62 trials.

1.3.2. Measurement of PPI and startle

We measured PPI 4-9 days after the last day of CPP (mean=7.13 days; median=7 days). Median age of mice at the time of testing was 68 days (mean=69.2, range=49-115). The PPI system was calibrated at the start of each testing day according to the manufacturer's instructions. Mice were transferred to the testing room one cage at a time, weighed, and then placed into the testing chamber. Mice were returned to their home cage after testing. PPI chambers were cleaned between sessions. Only the mice being tested were in the testing room to avoid exposing animals to the startling stimuli prior to the beginning of the test.

1.3.3. Analysis of PPI and startle

The startle response was calculated as the mean startle response across the startle-alone trials in blocks 1-4. We also performed GWAS for startle amplitude in blocks 1-4 separately. We define habituation to startle as the difference between the mean startle response during the first and last block of pulse-alone trials.

We define PPI as the normalized difference between two values: the mean startle response during pulse-alone trials and the mean startle response during prepulse trials. The first value is the raw

startle phenotype. The second value is calculated for each level of prepulse intensity (3, 6 and 12 dB above the 70-dB background noise) and divided by the raw startle value to obtain a proportion, which we transformed with the logit-10 function. To avoid extreme values caused by logit transformation of negative PPI values, we projected the transformed data onto an interval between 0.01 and 0.99, as described previously [26]. Startle values, which are positive, were transformed with the log10 function.

After transformation, we examined the distribution of startle responses during the no-stimulus trials to check for technical errors. Forty-four mice seemed to startle in the absence of a pulse (**Supplementary Fig. 20**), which we interpreted as a technical problem since all of these mice were tested in PPI box 3. We retained these mice in the analysis and included box 3 as a covariate for all PPI and startle phenotypes. Another group of mice had unusually low startle responses (**Supplementary Fig. 20**). It is likely that these mice are hearing-impaired because their startle responses overlapped the trait distribution for the no-stimulus trials (this was true once the 44 mice tested in box 3 were excluded due to the technical artifact mentioned above). PPI-related phenotypes for 13 mice with a mean startle response of 1.1 units or lower were marked as missing. Data were quantile-normalized prior to GWAS.

1.4. Fasting blood glucose levels

We measured blood glucose levels after a 4-hour fast 4-14 days (mean=7.3 days, median=7) after PPI testing. Median age of mice at the time of testing was 75 days (mean=76.4, range=56-122). Mice were brought into the testing room between 09:00 and 09:30 and transferred to new cages that did not contain food. After four hours of fasting, we weighed each mouse and used a razor blade to make a small incision at the tip of the tail, which allowed us to obtain a small drop of blood that we analyzed with glucose strips (Bayer Contour TS Blood Glucose Test Strips) and a glucometer (Bayer Contour TS Blood Glucose Monitoring System). Glucose levels are expressed in mg/dL units. Once all of the mice in a cage were tested, we gave them fresh food and returned them to the colony. Glucose measurements were quantile-normalized before performing GWAS.

1.5. Coat color

The LG x SM AIL segregates three coat color phenotypes. LG has a white (albino) coat. The SM strain is fully inbred except at the *agouti* locus on chromosome 2, where attempts to maintain a homozygous state have been unsuccessful; thus, LG x SM AIL mice can be either white (albino), black or brown (agouti) [27]. We transformed coat color into three indicator variables and treated them as quantitative traits for GWAS. We found this approach acceptable for testing our method because the genetic basis of coat color is well-known. Although GEMMA's linear mixed model (**LMM**) was intended for quantitative analysis, its robustness to model misspecification makes it acceptable for mapping factors expressed as binary indicator variables [28].

Because we did not expect to identify novel coat color loci, we did not consider coat color in the sum of 118 traits that we measured. Similarly, coat color did not contribute to the total number of associations reported in **Supplementary Data 1**. However, we provided heritability estimates for black, white and agouti coat in **Supplementary Data 2** as a benchmark for comparison to the quantitative traits of interest.

1.6. Wildness

Laboratory mice are known to vary in their ease of handling, or wildness [29]. Studying wildness could provide insight into the genetic consequences of domestication. We defined wild mice as those who escaped their home or holding cages in the moments leading up to the CPP test. Raw escape counts were converted into a binary indicator variable to account for increased experience in mouse handling by the experimenter. We did not find any loci associated with wildness, potentially due to lack of power (less than 10% of all mice were qualified as wild by our criteria). We also considered wildness as a potential covariate but found no evidence of its effect on the other traits.

1.7. Tissue collection

We collected tissues for DNA and RNA extraction and additional phenotyping by our collaborators 4-15 days (mean=7.46, median=7) after measuring glucose levels. We also measured body weight and tail length (cm from base to tip of the tail) at this time. Median age at death was 83 days (mean=84.4, range=64-129). Mice were removed from the colony immediately before dissection, weighed and killed using cervical dislocation followed by rapid decapitation and evisceration.

1.8. Hind limb muscle and bone

We phenotyped five muscles: two dorsiflexors, tibialis anterior (**TA**) and extensor digitorum longus (**EDL**), and three plantar flexors, gastrocnemius, plantaris and soleus. These muscles were selected because they differ in size and constitution of fiber types. Of the fast-twitch muscles, TA and gastroc are largest and express the entire range of type 2 fibers and some type 1 fibers. EDL and plantaris are smaller fast-twitch muscles comprised mainly of type 2B, 2X and 2A fibers. Soleus, a slow-twitch muscle, is comprised mostly type 1, 2A and 2X fibers [30]. Different morphological and functional properties are associated with each fiber type [31], and we reasoned that muscles composed of different types might be regulated by distinct genetic mechanisms.

Tibia length is indicative of skeleton size, and elongation of bones is associated with longer, larger muscles. Therefore, in order to isolate muscle-specific loci (as opposed to loci that regulate growth across multiple tissues) we included tibia length as a covariate in muscle mass GWAS. Skeletal muscle contributes substantially to body weight in mammals. To avoid circular correction, which would reduce power to detect muscle weight loci, we did not use body weight as a covariate for muscle traits. All hind limb traits were quantile-normalized before GWAS.

1.9. Locomotor phenotypes in G34 and *Csmd1* mutant mice

Similar to LG x SM G50-56, locomotor behavior for G34 [32] and for *Csmd1* mutant mice was measured in six 5-min bins for a total of 30 min. We did not observe covariate effects on locomotor behavior in *Csmd1* mutant mice; therefore, we used raw phenotype data to produce **Fig.4E**. However, we quantile-normalized phenotypes for G34 after regressing out the effects of sex, testing chamber, and body weight (**Fig.4D**). Covariates for G50-56 (listed in **Supplementary Data 2**) were also removed before quantile-normalizing the data to produce **Fig.4C.**

2. Genotying by sequencing (**GBS**) quality control

Mislabeling and sample mix-ups are common in large genetic studies and can reduce power for GWAS[33]. We were concerned about the possibility of samples being mistakenly swapped or mislabeled. Therefore, we called variants in two stages. First-pass variant calls were used to identify and resolve sample mix-ups (Section 2.1). In stage two, after correcting or discarding sample mix-ups, we repeated variant calling from scratch (Section 2.2).

2.1. Identification of GBS sample mix-ups

We used the ratio of reads that mapped to the X and Y chromosomes to validate the sex of each mouse. GBS data from LG, SM, and F1 controls (data sequenced from the same animals across multiple flow cells) and 24 mice that were genotyped using both GBS and the GigaMUGA were used as benchmarks, since the sex of these samples could be verified. For true females, we consistently observed that the number of X chromosome reads was an order of magnitude greater than the number of Y chromosome reads. However, a difference greater than one order of magnitude was never observed for true males. Twelve samples that violated these criteria were flagged as potential sex swaps. We then checked breeding records, mouse cage cards, pedigree information, and experiment logs to determine if the source of each error was typographical. We identified two female mice that were incorrectly labeled as male and corrected these typos in our records. However, we did not reassign mouse IDs for the 10

remaining samples at this time. To do this, we examined kinship estimates from genetic and pedigree data.

Most mice in our sample had an opposite-sex sibling, which allowed us to identify errors by comparing pedigree kinship to the realized relationships estimated from genetic data using IBDLD[34,35]. To calculate genetic kinship with IBDLD, we used first-pass genotypes called using ANGSD [36] and Beagle [37]. Here, we required that only 15% of the samples have reads at a given site in order for a call to be made. We removed first-pass variants with MAF<0.01 before using Beagle to fill in missing genotypes at 106,180 loci. We did not impute from a reference panel at this stage; instead, we inferred missing data from LD within the sample. This ensured that all mice had a genotype at each empirically typed GBS allele while avoiding perpetuating widespread errors by imputing from a reference panel or pedigree.

The purpose of using less stringent criteria for first-pass calls than for the final call set was to ensure that we would have a sufficient number of overlapping GBS genotypes from Beagle to compare against GigaMUGA genotypes, which we obtained for 24 mice that were genotyped using GBS. The GigaMUGA contains probes for over 143,000 SNPs [38]. After removing GigaMUGA SNPs with an Illumina quality score <0.7, we were left with 115,478 SNPs, only 24,934 of which were known to be polymorphic in LG and SM [39];**Supplementary Fig. 1**). As shown in **Supplementary Data 1**, we evaluated concordance among overlapping GBS and GigaMUGA genotypes at multiple stages to guide filtering and gauge the efficacy of our variant calling pipeline; for example, using a more stringent threshold for imputation quality (dosage r^2; **DR^2**) resulted in greater genotype concordance.

Approximately 86,180 first-pass Beagle variants with $DR^2>0.7$ and MAF>0.1 were used to calculate genetic kinship coefficients with IBDLD [34,35]. Pedigree kinship was calculated using a custom R script (see **URLs**). We estimated both types of kinship for every possible pair of mice in the sample. We compared the estimates by subsetting the data into sibling pairs and non-sibling pairs, which we identified using pedigree data. We flagged non-siblings with higher than average kinship and siblings with lower than average kinship compared to the rest of the subset. We identified 21 non-sibling pairs with unusually high genetic kinship and 22 sibling pairs with unusually low genetic kinship, 8 of which had already been flagged as sex swaps. In some cases, apparent mix-ups were caused by typos; we resolved these by comparing cage cards against breeding logs and experiment records. We also verified homozygous LG genotypes at the *Tyr* locus for mice listed as albino. When possible, we cross-checked GBS genotypes with RNA-seq genotypes (described in Section 3).

Ultimately there were 15 out of 1,078 samples whose identities could not be resolved (some of these mice did not have a sibling in the data). These mice were included in the process of variant calling for the final sample because they provided additional information for obtaining genotype likelihoods in ANGSD. However, they were discarded before imputation and were not used for mapping. In the error-corrected data, GBS and GigaMUGA genotype concordance for 18,278 overlapping SNPs after reference panel imputation and filtering was 97.4% (**Supplementary Data 1**). This is similar to concordance rates observed for other animal populations genotyped with GBS [26,40] and falls within the range of imputation concordance rates reported in human studies [41,42].

2.2. Variant calling and quality control for error-corrected data (n=1,063)

2.2.1. Genotype likelihoods

We used an implementation of the Samtools [43] variant calling algorithm in ANGSD [36]to obtain genotype likelihoods at 899,436 sites for which at least 20% of samples had reads. GBS produces variable coverage across individuals, which leads to highly heterogeneous call rates. In addition, GBS has a bias toward homozygous calls [44]. Accordingly, we expected ANGSD's allele frequency estimates to be biased and used a lenient MAF threshold of 0.005 to filter raw genotype likelihoods.

2.2.2. Imputation

We used Beagle [37,45] to call genotypes from ANGSD likelihoods at 221,091 autosomal sites that passed our filters. When a reference panel is provided, Beagle requires hard genotype calls as input; however, ANGSD only outputs likelihoods. Therefore, we imputed missing genotypes in three steps. First, we used Beagle to phase and fill in missing calls using within-sample LD (no reference panel or pedigree was

provided). This produces a file with hard genotype calls, genotype probabilities and dosages that can be used to impute additional genotypes from an external reference panel. Next, we excluded SNPs with MAFs <0.1, leaving 38,238 variants for step three (we used this threshold because both alleles are expected to be common in an AIL; this was confirmed in G34 mice [32]). We then used Beagle to impute untyped SNPs from LG and SM reference haplotypes [39]. The JAX Mouse Map Converter (see **URLs**) was used to create a genetic map from mm10 base pair coordinates [46]. We retained 3.4M variants with very high imputation quality (DR^2>=0.9) and MAF>0.1 for further analysis.

2.2.3. Hardy-Weinberg Equilibrium (**HWE**)

An AIL is not a randomly mating population, its effective population size is not infinitely large, and LD is extensive. Selecting an appropriate threshold for excluding HWE deviations is further complicated by the tendency of GBS to wrongly call heterozygous genotypes as homozygous. We ran 1,000 gene dropping simulations in QTLRel [47]to simulate null genotypes consistent with the AIL pedigree (but not impacted by the overrepresentation of homozygotes that is observed when using GBS). We used the R package Hardy Weinberg [48]to test simulated genotypes for deviation from HWE. To reduce the computational burden of gene dropping, we restricted our analysis to 372,995 SNPs with unique centimorgan (cM) positions [46]. Chi-squared p-values $\leq 7.62 \times 10^{-6}$ were only detected for 1% of simulated genotypes. We used this value to identify loci where the observed genotype proportions constituted a significant deviation from HWE given that the data are from an AIL. 52,466 SNPs (1.5% out of 3.4M imputed SNPs with $DR^2 \geq 0.9$ and MAF≥0.1) were found to deviate from HWE at $p \leq 7.62 \times 10^{-6}$ and were excluded.

2.2.4 Linkage disequilibrium (**LD**)

Finally, we used PLINK to remove variants in high LD (r^2>0.95), leaving 523,028 SNPs for GWAS and eQTL mapping [49].

3. Identification and correction of RNA sample mix-ups

To identify samples that were apparently mislabeled with the incorrect mouse ID, we retrieved allele counts from each sample at sites with ≥25 reads and no more than one mismatched base; we used these data to produce RNA-seq genotype calls. We obtained up to three sets of RNA-seq genotypes (one per tissue). We measured RNA genotype concordance for all pairs of samples, expecting that the best match for each sample would be from a different tissue belonging to the same mouse. If the best match belonged to a different mouse, the samples were flagged as potential mix-ups.

Next, we examined concordance between RNA and error-corrected GBS genotypes from phase one to reassign mixed-up sample IDs. If we could not resolve the identity of a sample, we discarded its expression data. 108 samples were discarded during this process (33 HIP; 36 PFC; 39 STR). We also discarded expression data for 29 samples whose genotype data was removed during GBS quality control (11 HIP; 9 PFC; 9 STR). The mean concordance rate among RNA genotypes derived from the same mouse was 94.6% after error correction, indicating that our approach was successful.

We also examined *Xist* (X-inactive specific transcript) expression to identify apparent sex mix-ups. *Xist* regulates dosage compensation and is only expressed in females. One STR sample associated with a male mouse (54896_STR) had high *Xist* gene expression. One HIP and one STR sample associated with the same female mouse (56203_HIP; 56203_STR) had no *Xist* gene expression. We reassigned the sex of the two female tissue samples to male, since both samples belonged to the correct tissue and their RNA genotypes indicated that they were extracted from the same mouse. We excluded 54896_STR from further analyses due to a lack of evidence for sample reassignment.

Finally, we used correlation-based statistics to identify and remove 12 additional outliers (2 HIP; 7 PFC; 3 STR). For each tissue, we computed the mean correlation of expression level for an individual i:

$$\overline{r_i} = \sum_j r_{ij}/(n-1)$$

Where n is the number of mice in a tissue, j is remaining mice in the tissue that are not i, and r_{ij} is the gene expression correlation between an individual i and j. We considered an individual i as an outlier if $\overline{r_i} < 0.9$.

4. Genome-wide significance thresholds

4.1. Multiple hypothesis testing correction for GWAS

Because LD is greater in AILs than in human populations, we did not use $5\text{x}10^{-8}$ as a significance threshold. Furthermore, the complex relationships in an AIL imply a covariance among phenotypes and genotypes that makes the standard Bonferroni adjustment for multiple testing correction too conservative. Naïve permutation can effectively correct for multiple hypothesis testing even when the assumption of independence among phenotypes and genotypes is violated by relatedness[50,51]. Parametric bootstrapping can provide greater accuracy, but the computational expense of resampling null phenotypes and calculating their test statistics while also maintaining the covariance structure of the data makes it impractical for large studies [50].

We used MultiTrans[52] and SLIDE [53] to obtain a genome-wide significance threshold for GWAS because when combined, they offer a compromise between the two methods. Like parametric bootstrapping, MultiTrans estimates the phenotypic covariance (*V*) under an LMM. Rather than proceeding to sample phenotypes from a multivariate normal distribution with covariance *V*, it uses *V* to transform the genotype data such that the correlation between transformed genotypes is equivalent to the correlation among test statistics sampled from an MVN [52]. This improves efficiency because it obviates the need to generate null phenotypes and calculate their p-values. Instead, p-values are sampled directly from a multivariate normal distribution using SLIDE, which accounts for LD between nearby markers.

We specified a sliding window of 5,000 SNPs and used 2.5 million samples to obtain a per-marker threshold of $p=8.06\text{x}10^{-6}$ given a genome-wide significance threshold (α) of 0.05. Because all phenotypic data was quantile-normalized, we applied the same threshold to all phenotypes.

4.2. LOD drop intervals

We considered using bootstrapping to estimate a confidence interval around each associated region; however, it is not clear that this approach would have been effective enough to justify the high computational cost [50]. Interval coverage depends on locus effect size, chromosome size, SNP density, and the location of the causal SNP in relation to the associated markers. Instead we converted p-values to LOD scores and used a 1.5-LOD support interval to approximate a critical region around each association. The LOD drop approach provides a quick, straightforward way to gauge mapping precision and systematically identify overlap between eQTL genes and candidate QTGs; however, it does not correspond to a specific confidence interval (e.g. 95% confidence interval).

4.3. Significance of *trans*-eQTLs

These *trans*-eQTLs called “significant” in this study were genome-wide significant at α=0.05 after correction for the individual genome scan, but before attempting any correction for the number of genes tested. Given that α=0.05, we expected that 5% of genes for which the null hypothesis was correct would be declared “significant” by this standard. We performed an internal replication analysis of *trans*-eQTLs in HIP, PFC and STR by randomly splitting each sample in half and separately mapping eQTLs in the two subsets. We used the Wald test (`lmm -1` option in GEMMA), which yields effect size estimates (β) for each SNP. We then examined the effect size of each SNP that was called significant in the full sample, comparing the sign of the effect across the two subsets in each tissue. For HIP, 95.2% of effect sizes at significant *trans*-eQTLs were concordant among the random subsets (Pearson’s r^2=0.922). For PFC, effect size concordance was 84.8% (Pearson’s r^2=0.889). For STR, effect size concordance was 99.7% (Pearson’s r^2=0.923). We note that this test does not estimate the study-wide false positive rate because the subsets are not independent of the discovery set. Thus, beta estimates are expected to be biased in a way that makes replication more likely.

5. Software and URLs

We have provided commands used to analyze the data described in this study in the **Supplementary Note**. We have also submitted phenotype, genotype and gene expression data from this study to www.GeneNetwork.org. Here we provide a list of the software we used for the analyses in this paper with the minimum version number and a brief description of how we used it.

5.1. R packages

*All of the R packages below were run using **R v.3.1.0** or higher.*
permute v.0.9-4: permuted phenotypes and genotypes for significance thresholds
leaps v.2.9: covariate selection for AIL phenotypes
SOFIA v.1.0: automatic generation of files formatted for use in Circos software [54]
DEseq v.1.24.0: processing RNA sequencing reads [55]
QTLRel v.0.2-15: gene dropping simulations for HWE test [47]
HardyWeinberg v.1.5.6: HWE test for AIL genotypes and gene dropping simulations [48]
GenomicAlignments v.1.8.4: genome assembly for RNA-seq reads [56]
ggpubr v.0.1.5; **ggplot2 v.2.2.1** [57]; **viridis v.0.4.0**; **VennDiagram v.1.6.17** [58]: plotting tools

5.2. Other software

BWA v.0.7.5a: sequencing read alignment [59]
GATK v.3.3.0: base quality score recalibration and indel realignment [60]
GEMMA v.0.94: QTL/eQTL mapping; heritability estimation; GRM calculation [28,61,62]
PLINK v.1.9: genotype file processing; LD pruning [49]
ANGSD v.0.912: genotype likelihoods/variant calling [36]
Beagle v.4.1: genotype imputation [37,45]
IBDLD v.3.34: pedigree error checking [34,35]
HISAT v.0.1.6: RNA-seq read alignment [63]
Circos v.0.69-5: eQTL figures [64]
CASAVA v.1.6: demultiplexing RNA-seq reads (Illumina, San Diego, USA)
MultiTrans (no version number) [52] and **SLIDE v.1.0.4**[53]: QTL significance thresholds
bcftools v.1.3; **picard-tools v.1.92**; **samtools v.1.2** [65]: file formatting; summary statistics

5.3 URLs

BreedAIL.R; R script to select AIL breeders
https://github.com/pcarbo/breedail
dbSNP v.142 data; SNP annotation and rsids (file: snp142.txt.gz)
http://hgdownload.cse.ucsc.edu/goldenPath/mm10/database/
Ensembl; gene coordinates, transcript and regulatory annotations
http://www.ensembl.org/Mus_musculus/Info/Index
JAX Mouse Map Converter; bp to cM conversion for genetic map
http://cgd.jax.org/mousemapconverter/
Mouse Genome Informatics (MGI); gene-level queries
http://www.informatics.jax.org/
UCSC Genome Browser; mm10 reference genome, gene and SNP information, liftOver
http://genome.ucsc.edu/cgi-bin/hgGateway
GeneNetwork; AIL data is available here
http://www.genenetwork.org

Supplementary Table 1

	unfiltered	MAF ≥ 0.1	MAF ≥ 0.1<br>DR^2 ≥ 0.9	MAF ≥ 0.1<br>DR^2 ≥ 0.9<br>HWE p ≤ 7.62e-6	MAF ≥ 0.1<br>DR^2 ≥ 0.9<br>HWE p ≤ 7.62e-6<br>LD (r^2 > 0.95)
number of SNPs	24,907	24,886	18,486	18,278	3,678
number of calls (24 mice)	593,949	593,457	440,845	435,867	87,867
mean concordance rate	0.944	0.944	0.973	0.974	0.974
number of GBS HET calls	254,100	253,981	193,830	191,885	38,711
proportion GBS HET calls / total calls	0.428	0.428	0.440	0.440	0.441
number of GBS HOM calls	339,849	339,476	247,015	243,982	49,156
proportion GBS HOM calls / total calls	0.572	0.572	0.560	0.560	0.559
number of chip HET calls	251,102	250,908	190,764	188,557	38,180
proportion of chip HET calls	0.423	0.423	0.433	0.433	0.435
number of correct GBS HET calls	238,834	238,747	187,543	185,676	37,558
proportion of correct GBS HET calls	0.951	0.952	0.983	0.985	0.984
number of GBS HET calls where true genotype is HOM	15,266	15,234	6,287	6,209	1,153
proportion of GBS HET calls where true genotype is HOM	0.060	0.060	0.032	0.032	0.030
number of GBS HOM calls where true genotype is HET	12,268	12,161	3,221	2,881	622
proportion of GBS HOM calls where true genotype is HET	0.036	0.036	0.013	0.012	0.013
number of GBS HOM calls that are flipped	5,846	5,839	2,482	2,450	538
proportion of GBS HOM calls that are flipped	0.017	0.017	0.010	0.010	0.011

GBS and GigaMUGA genotype concordance. Concordance among genotypes called by the GigaMUGA genotyping array and GBS genotype calls in 24 mice are provided at various levels of filtering. HET and HOM refer to heterozygous and homozygous genotype calls. MAF is minor allele frequency, DR^2 is dosage r-squared (a measure of imputation accuracy used by Beagle), LD is linkage disequilibrium, and HWE is Hardy-Weinberg Equilibrium. HWE p-values are from a Chi-squared test (see Supplementary Methods for details).

Supplementary Note 1

AIL pipeline. Example commands and software used for the analyses described in this paper are provided below. A complete list of software with version numbers and references is provided in the **Supplementary Methods**.

```
## ------------------------------------------------------------------------------------------
## AIL Pipeline
## ------------------------------------------------------------------------------------------

## DEMULTIPLEX AND QC -----------------------------------------------------------------------
## Input: Per-lane fastq files, a tab-delimited file with sample id, barcode sequence, library
name in columns 1-3.
## Requirements: demux_GBS_PstI.py (below), python v.2.7.6 or other compatible version.
demux_GBS.PstI.py --library $LIBRARY --infirst $FILE_LIST --adapters $BARCODE_KEY --zipped

#! /usr/bin/env python
#########################################################
# Code to demux the GBS raw sequence files using the li- #
# rary number. The input files are read from other files #
#########################################################
# You could import using the grammar 'from sys import argv' so you could type
# argv instead of sys.argv with each use... but know that this decreases
# readability and could result in name clashes.

import os           # misc OS interfaces
import re           # regular expression operations
import argparse     # parser for cmd line options, args and sub-cmds
import gzip         # support for gzip files
import numpy as np  # NUMerical PYthon for scientific computing
                    # np = alias for numpy. purpose: avoid namespace conflicts.
import sys          # system-specific params and fxns
import datetime     # basic date and time types


if (__name__=='__main__'):  # 'is __name__ being run standalone by the user?'


    parser = argparse.ArgumentParser(description='Demultiplexing script for GBS')


    parser.add_argument('-l', '--library', metavar='Library', type=str,
                        dest='lib', help='Library to demultiplex',
                        required=True)


    parser.add_argument('-1', '--infirst', metavar='LeftFile', type=str,
                        dest='left',
                        help='File of input filenames (left reads if paired)',
                        required=True)


    parser.add_argument('-2', '--insecond', metavar='RightFile', type=str,
                        dest='right', help='File of right read filenames',
                        required=False, default='')

    parser.add_argument('-s', '--adapters', metavar='AdapterFile', type=str,
                        dest='sidfname', help='Adapter file (tab separated)',
                        required=True)


    parser.add_argument('-z', '--zipped', dest='zipped',
                        help='Input file zipped', action='store_true')

    parser.add_argument('-p', '--pairedEnd', dest='paired',
                        help='Paired end reads', action='store_true')

    parser.add_argument('-q', '--qseqs', dest='qseq',
                        help='Qseq files not sequence files',
                        action='store_true')

    parser.add_argument('-u', '--unzipoutput', dest='unzipout',
                        help='Unzipped output', action='store_true')
```

```
    parser.add_argument('-e', '--enzymeSuffix', dest='enzyme',
                        help='Overhang of Enzyme', required=False,
                        default='TGCAG')


    args = parser.parse_args()


# Hamming distance between 2 strings of equal length is the num of positions
# at which the corresponding symbols are different. E.g. it measure the min
# num of substitutions/errors required to change one string into the other.
def hamming (s1, s2):
    """
    Computes the hamming distance between
    2 strings of bases.
    """
    if len(s1) != len(s2): return 50    # 50= #bases in common adapter seq?
    dist = 0
    for i in range(len(s1)):
        if s1[i] != s2[i]: dist += 1
    return dist


def nearestNeigh(readstr, adapters):
    """
    This function calculates the hamming distance
    between the read and all the adapters.
    Returns the nearest adapter if the minimium
    hamming distance is 0 or 1. If not, returns
    NULL.
    """
    minDist = 50
    nearest = None
    numnearest = 0
    for aid in adapters:
        cd = hamming(readstr[0:len(aid)], aid)
        if cd < 2:
            if cd < minDist:
                minDist = cd
                nearest = aid
                numnearest += 1
            elif cd == minDist:
                numnearest += 1
                nearest = None
    if numnearest > 1: return None
    return nearest

# sidfname is the adapter file name (-s arg above)
sidfile = open(args.sidfname)
#enzymeSuffix = 'TGCAG' #works only for PstI
# enzyme suffix is given after -e; default is 'TGCAG'
enzymeSuffix = args.enzyme
line = sidfile.readline() # gets rid of header
sids = {}   # sid stands for sample id?
for line in sidfile:
    line = line.strip() # line is a string with whitespace removed
    toks = line.split() # alias for splitting a string into subunits

    adap = toks[1].upper()+enzymeSuffix # make subunit[1] (the idx) an uppercase
                                        # string & cat(TGCAG) to the end
    if (args.lib == toks[2]):   # the toks[2] must be the library name ('AIL50')
        sids[adap] = toks[0]    # toks[0] is the mouse id
sidfile.close()

# the function below handles paired end reads and zipped files
fhands_1 = {}
if (args.paired):
    fhands_2 = {}
if (args.unzipout):
    for name in np.unique(sids.values()):
        ftemp = open(name+'.fq', 'w')
        fhands_1[name] = ftemp
        if (args.paired):
            ftemp = open(name+'.fq', 'w')
            fhands_2[name] = ftemp
else:
```

```
    for name in np.unique(sids.values()):
        ftemp = gzip.open(name+'.fq.gz', 'w')
        fhands_1[name] = ftemp
        if (args.paired):
            ftemp = gzip.open(name+'.fq.gz', 'w')
            fhands_2[name] = ftemp

cntReads = 0
success = 0
firsts = open(args.left)  # this will be the only files for SE reads
if args.right != "":      # e.g. 'if paired end' there will be righthand reads
    seconds = open(args.right)
if (args.qseq):           # handles qseq files
    fs=[x.strip() for x in firsts.readlines()]
    for fname1 in fs:
        if(args.paired):
            fname3 = seconds.readline().strip()
            if (args.zipped):
                f3=gzip.open(fname3)
            else:
                f3=open(fname3)
        if (args.zipped):
            f1=gzip.open(fname1)
        else:
            f1=open(fname1)
        if (args.paired):
            for l1, l3 in zip(f1, f3):
                l1=l1.strip()
                t1=l1.split()
                l3=l3.split()
                t3=l3.split()
                nearest = nearestNeigh(t1[8], sids)
                if(nearest != None):
                    fhands_1[sids[nearest]].write('@'+':'.join(t1[0:8])+'\n')

fhands_1[sids[nearest]].write(t1[8][len(nearest):]+'\n+\n'+t1[9][len(nearest):]+'\n')
                    fhands_2[sids[nearest]].write('@'+':'.join(t3[0:8])+'\n')

fhands_2[sids[nearest]].write(t3[8][len(nearest):]+'\n+\n'+t3[9][len(nearest):]+'\n')
        else:
            for l1 in f1:
                l1=l1.strip()
                t1=l1.split()
                nearest = nearestNeigh(t1[8], sids)
                if (nearest != None):
                    fhands_1[sids[nearest]].write('@'+':'.join(t1[0:8])+'\n')

fhands_1[sids[nearest]].write(t1[8][len(nearest):]+'\n+\n'+t1[9][len(nearest):]+'\n')
        f1.close()
        if(args.paired):
            f3.close()
else:
#######NOT A QSEQ -- implement part for fq files.
    sname = ''
    curcnt = 0
    if (args.paired):
        print 'Cannot deal with paired end data with fq input file.'
        sys.exit(1)
    files=os.listdir('.')
    fs=[x.strip() for x in firsts.readlines()]
    print 'Processing', len(fs), 'files.'
    for fname1 in fs:
        if (args.zipped):
            f1=gzip.open(fname1)
        else:
             f1=open(fname1)
        for l1 in f1:
            if (curcnt == 0):
                if (len(l1) == 0):
                    break
                l1=l1.strip()
                if l1[0] == '@':
                    if (sname == ''):
                        sname = l1[1:]
                    else:
                        print 'Error after reading read', sname, l1
                        print 'Read', cntReads, 'reads.'
```

```
                        sys.exit(2)
                curcnt += 1
            elif (curcnt == 1):
                seq = l1.strip()
                curcnt += 1
            elif (curcnt == 2):
                if l1[0] != '+':
                    print 'Error after reading read -', sname, '- -', l1, '-'
                    print 'Read', cntReads, 'reads.'
                    sys.exit(3)
                curcnt += 1
            elif (curcnt == 3):
                qual = l1.strip()
                nearest = nearestNeigh(seq, sids)
                if (nearest != None):
                    success += 1
                    fhands_1[sids[nearest]].write('@'+sname+'\n')

fhands_1[sids[nearest]].write(seq[len(nearest):]+'\n+\n'+qual[len(nearest):]+'\n')
                curcnt = 0
                sname = ''
                cntReads += 1
                if (cntReads % 100000 == 0):
                    print datetime.datetime.now().time(), ': Processed', cntReads, 'reads.'
                    sys.stdout.flush()
        f1.close()
  print "Done processing file."

for name in np.unique(sids.values()):
    fhands_1[name].close()
    if (args.paired):
        fhands_2[name].close()

firsts.close()
if args.right != "":
    seconds.close()
print 'Number of reads processed:', success, '/', cntReads



## ALIGNMENT ----------------------------------------------------------------------------
## Input: demultiplexed and gzipped fastq files
## Requirements: bwa v.0.7.5a

# our cluster uses the Torque/PBS scheduler; we submit each line in the list of fq.gz files as
# an array, e.g. `qsub -t 1-100 align.mm10.sh` where the numbers = $PBS_ARRAYID
LIBRARY="AIL99"
INFILE=`head -$PBS_ARRAYID $LIBRARY.list | tail -1`
SAMPID=`basename $INFILE .fq.gz`
LANE="4"
MACHINERUN="K00180.172"
CENTER="Chicago"
REFGENOME='mm10.fa.gz'
STDIN='/dev/stdin'
RG="@RG\tID:$MACHINERUN.$LANE\tPU:$MACHINERUN.$LANE\tSM:$SAMPID\tCN:$CENTER\tPL:Illumina"

echo "Running alignment for $INFILE"
if [ ! -s ${SAMPID}.bam ]; then
  bwa mem -M -R "$RG" $REFGENOME $INFILE | \
  java -Xmx4g -jar picard-tools-1.126/picard.jar SortSam \
    INPUT=$STDIN \
    OUTPUT=$OUTDIR/$SAMPID.bwa.bam \
    SORT_ORDER=coordinate \
    CREATE_INDEX=true
fi

exitstatus=( ${PIPESTATUS[@]} )
if [ ${exitstatus[0]}  != 0 ]; then
  echo "ERROR: Failed bwa mem with exit code "${exitstatus[0]}
  exit ${exitstatus[0]}
elif [ ${exitstatus[1]} != 0 ]; then
  echo "ERROR: Could not sort and make the bam file from sam file "${exitstatus[1]}
  exit ${exitstatus[1]}
else
  echo "Successfully mapped the reads and then made, sorted and indexed the bam file "
fi
```

```
## STANDARDIZE PHRED QUALITY SCORES -----------------------------------------------------------
## Input: aligned, coordinate-sorted and indexed .bam files
## Requirements: samtools v.1.2, convertQualScoreBam
## Code obtained from Devon Ryan at http://seqanswers.com/forums/showthread.php?t=39549

## Determine whether the file is encoded in Illumina 1.3 or 1.5 (phred+64) or Illumina 1.9
(phred+33)
## by looking at the first 100k reads of each file. Convert to phred+33 format.

## make a list of commands to process multiple files at once as part of a Torque/PBS task
array.
for bamfile in /group/palmer-lab/AIL/GBS/bams/controls/*bam; do
    n64=`samtools view $i | cut -f11 | head -100000 | grep -c [a-h]`
    n32=`samtools view $i | cut -f11 | head -100000 | grep -c [0-9]`
    ns64=`samtools view $i | cut -f11 | head -100000 | grep -c [\<\=\>?]`
    if [ $n32 -eq 0 ]; then
    if [ $ns64 -ne 0 ]; then
        echo $bamfile Solexa - ignoring file because Solexa is obsolete
    else
        echo convertQualScoreBam $bamfile >> requal.cmds
    fi
    else
    if [ $n64 -eq 0 ]; then
        echo mv $bamfile ${bamfile/.bam/.requaled.bam} >> requal.cmds
    else
        echo $bamfile Impossible $n32 $n64 $ns64 - so ignoring file.
    fi
    fi
done

/****************************************************
Code obtained from online seqanswers thread
http://seqanswers.com/forums/showthread.php?t=39549
Courtesy of Devon Ryan (userid dpryan).
****************************************************/
#include "bam.h"

int main(int argc, char *argv[]) {
  int i;
  uint8_t *qual;
  bam_header_t *header;
  char *oname, *p;
  bam1_t *read;
  bamFile f, of;

  if(argc != 2 || strcmp(argv[1], "-h") == 0) {
    printf("Usage: %s file.bam\n", argv[0]);
    printf("Converts a BAM file with Phred scores encoded as QUAL+64 into the standard QUAL+33
format\n");
    return -1;
  }

  //Open the input
  f = bam_open(argv[1], "rb");
  header = bam_header_read(f);
  read = bam_init1();

  //Create the output file
  oname = strdup(argv[1]);
  oname = realloc(oname, sizeof(char) * (strlen(argv[1]) + strlen(".requaled ")));
  p = strrchr(oname, '.');
  *p = '\0';
  sprintf(oname, "%s.requaled.bam", oname);
  of = bam_open(oname, "wb");
  bgzf_mt(of, 4, 256); //Use 4 compression threads

  //Write the header
  bam_header_write(of, header);

  //Iterate over the reads
  while(bam_read1(f, read) > 1) {
    qual = bam1_qual(read);
    for(i=0; i<read->core.l_qseq; i++) *(qual+i) -= 31;
    bam_write1(of, read);
  }

  bam_close(of);
```

```
  bam_close(f);
  bam_destroy1(read);
  free(oname);
  return 0;
}


## INDEL REALIGNMENT --------------------------------------------------------------------------
## Input: Aligned, coord-sorted, requaled .bams
## Requirements: GATK v.3.3-0, java, interval list, mm10 dictionary

## make interval list
java -Xmx2g -jar /apps/software/GenomeAnalysisTK/3.3-0/GenomeAnalysisTK.jar \
     -T RealignerTargetCreator \
     -R:REFSEQ mm10.fasta \
     -known:VCF LG_SM_Indels.vcf \
     -o LG_SM.mm10.realign.intervals

## realign around target intervals
INFILE=`head -$PBS_ARRAYID realignFiles.list | tail -1`
BASE=`basename $INFILE .bam`
echo "Running realignment for: $INFILE"
java -Xmx2g -jar /apps/software/GenomeAnalysisTK/3.3-0/GenomeAnalysisTK.jar \
     -T IndelRealigner \
     -R mm10.fasta \
     -I $INFILE \
     -targetIntervals LG_SM.mm10.realign.intervals \
     -o $BASE.ra.bam
echo "Done running realignment."


## BASE RECALIBRATION --------------------------------------------------------------------------
## Input: Input: Aligned, coord-sorted, requaled, realigned, regrouped .bams
## Requirements: GATK v.3, samples.list files, R

## Generate first-pass recalibration table
java -Xmx9g -jar /apps/software/GenomeAnalysisTK/3.3-0/GenomeAnalysisTK.jar \
     -T BaseRecalibrator \
     -R:REFSEQ mm10.fasta \
     -I filesLane1.list \
     -knownSites:VCF LGSM.mm10.SNPs.vcf \
     -knownSites:VCF LGSM.mm10.Indels.vcf \
     -o bqsrLane1.table

## Generate second-pass recalibration table
java -Xmx9g -jar /apps/software/GenomeAnalysisTK/3.3-0/GenomeAnalysisTK.jar \
     -T BaseRecalibrator \
     -R:REFSEQ mm10.fasta \
     -I filesLane1.list \
     -knownSites:VCF LGSM.mm10.SNPs.vcf \
     -knownSites:VCF LGSM.mm10.Indels.vcf \
     -BQSR bqsrLane1.table
     -o bqsrLane1_recal.table

## Apply recalibration and make new bam files.
java -Xmx9g -jar /apps/software/GenomeAnalysisTK/3.3-0/GenomeAnalysisTK.jar \
     -T PrintReads \
     -R:REFSEQ mm10.fasta \
     -I filesLane1.list \
     -BQSR bqsrLane1.table
     -o output.bam


## VARIANT CALLING --------------------------------------------------------------------
## 1. ANGSD - get genotype likelihoods.
## Input: Aligned, coord-sorted, requaled, realigned, regrouped bams, 1 file per sample.
## Requirements: angsd

INFILE="bamfiles.list"
window=10000000
for chrom in {1..19} X; do
   length=`grep -w chr$chrom chrLengths_mm10.txt | tr -s " " " " | cut -f2 -d " "`
   start=1
   end=0
   while [ $start -lt $length ]; do
     let end=start+window-1
     if [ $end -gt $length ]; then
```

```
      end=$length
    fi
  echo angsd -b $INFILE \
        -doVcf 1 \
        -gl 1 \
        -doMajorMinor 4 \
        -ref mm10.fasta \
        -doMaf 8 \
        -doCounts 1 \
        -snp_pval 1e-5 \
        -P 2 \
        -r chr$chrom:$start-$end \
        -minMapQ 20 \
        -minQ 20 \
        -doPost 2 \
        -minInd 216 \ ## 20% of sample
        -out chr$chrom.raw.$start
   let start=start+window
   done
done > angsd.cmds


## 2. Beagle round 1 (Within) - fill in missing genotypes using within-sample LD; make
genotype
## calls from ANGSD likelihoods to use for quality control and imputation from a reference
panel.
## Input: chrwise ANGSD genotype likelihood VCF files, genetic map
## Requirements: Beagle v.4.1

## recommended: make sure REF/ALT alleles are specified correctly with respect to the
## reference genome using bcftools v.1.3
bcftools norm \
           -c s \ # fixes bad sites where REF allele is wrong or missing
           -f mm10.fasta \
           -O z \
           -o outfile
           infile.vcf.gz

java -Xmx12g -jar ~/Beagle/beagle.27Jul16.86a.jar \
        gtgl=chr$chrom.raw.vcf.gz \
        out=chr$chrom.beagWithin \
        chrom=chr$PBS_ARRAYID \
        excludemarkers=excludeLowMafSnps.list \
        excludesamples=excludeErrorsMice.list \
        map=LGSM.genetic.map \
        window=300 \
        overlap=50 \
        gprobs=true \
        niterations=10 \
        err=0.04 \
        nthreads=4


## 3. Beagle round 2 (Ref) - impute from a LG/SM reference panel.
## Input: BeagleWithin VCF files with GT field, map, reference panel VCFs
## Requirements: Beagle v.4.1

java -Xmx12g -jar ~/Beagle/beagle.27Jul16.86a.jar \
        gt=chr$chrom.beagWithin.vcf.gz \
        ref=LGSM.chr$PBS_ARRAYID.phased.vcf.gz \
        out=chr$chrom.beagRef \
        chrom=chr$PBS_ARRAYID \
        excludemarkers=excludeLowMafSnps.list \
        map=LGSM.genetic.map \
        window=70000 \
        overlap=32000 \
        impute=true \
        niterations=10 \
        err=0.03 \
        nthreads=4

## KINSHIP ESTIMATES -----------------------------------------------------------------
## Use IBDLD to estimate kinship from genotypes and compare with pedigree kinship
## for identifying and resolving sample mix-ups.
## Input: genotypes in Plink PED/MAP format. Map file must list cM positions.
## Requirements: plink v.1.90, IBDLD v.3.34
```

```
## EXAMPLE IBDLD commands to obtain genetic kinship (different from the one
## generated by GEMMA for GWAS)
IBDLD -plink genos.ped \
      -m_int genos.map \
      -method GIBDLD \
      -ploci 50 \
      -dist 5 \
      -nthreads 4 \
      -error 0.035 \
      -ibc \
      -o genos.ibc

## R code to obtain pedigree kinship adapted from AIL breeder selection code.
## Author: Andrew Skol, University of Chicago
kinship <- function(ped) {

  ## ped should be a matrix with 4 cols in the follwing order:
  ## ID, Sire, Dam, Sex. Ped should be ordered from from founding
  ## generation to current. Sex may be coded as "M"/"F" or as 1/0.

  m.idx = ped[,4] == "M"
  f.idx = ped[,4] == "F"
  N = dim(ped)[1]

  if (sum(c(m.idx,f.idx)) == N){
    ped[m.idx,4] = 1
    ped[f.idx,4] = 0
  }else if (sum(c(m.idx,f.idx)) != 0) {
    sex.warning()
    return()
  } else if (sum(ped[,4] %in% c(0,1))<N){
    sex.warning()
    return()
  }

  ## Recode ids from 1:N ##
  ped.tmp = matrix(0,N,4)
  ped.tmp[,1] = 1:N
  for (i in 1:N){
    id.tmp = ped[i,1]
    idx = ped[,2]==id.tmp
    ped.tmp[idx,2] = i
    idx = ped[,3] == id.tmp
    ped.tmp[idx,3] = i
  }
  ped = ped.tmp

  ## Begin calculation ##
  kinship = diag(rep(1,N))
  for (i in 1:N){

    i.M = ped[i,2]
    i.F = ped[i,3]

    for (j in i:1){

      if (j==i){
        ## if j's parents are in the pedigree ##
        if (i.M!=0 & i.F!=0){
          kinship[i,j] = 1 + .5*kinship[i.M,i.F]
        }
      } else {
        if (i.M!=0 & i.F!=0){
          kinship[i,j] = .5*kinship[i.M,j] + .5*kinship[i.F,j]
          kinship[j,i] = kinship[i,j]
        }
      }

    }
  }
  return(kinship)
}


## GENE DROPPING SIMULATIONS ---------------------------------------------------------
## Input: reduced pedigree file that only includes lineages of G50-56 mice used for GWAS.
## A reduced genetic map that only includes one SNP from each unique cM position. These
```

```
## data were used in an attempt to reduce the large computational burden of gene dropping
## simulations.
## Requirements: R v.3.1 or higher; packages QTLRel and HardyWeinberg

## EXAMPLE SHELL SCRIPT
## e.g. `qsub -t 1-1000` geneDrop.sh such that the array id (-t) is
## interpreted by R as the seed.
R --vanilla --args seed $PBS_ARRAYID chrom 1 < ./geneDropHardy.R

## geneDropHardy.R
seed <- 0
chrom <- 0

library("batch")
parseCommandArgs(evaluate=TRUE)

     seed <- runif(1, min=0, max=2^31-1)
     seed <- round(seed,0)
     set.seed(seed)

     require("QTLRel")
     require("HardyWeinberg")
     load("pedReduced.50to56.geneDrop.RData")
     map <- read.table(paste0("./hardyMapReduced.chr", chrom, ".map.txt"),
                      sep="\t", head=TRUE, stringsAsFactors = FALSE, as.is=TRUE)

        drop <- genoSim(ped=pedReduced, gmap=map, ids=ids.ped, method="Haldane")
        drop <- MakeCounts(drop, coding=c(AA=1, BB=2, AB=3))
        drop <- drop[,1:3]
        mono <- (drop[,2]==0 & drop[,1]==0) | (drop[,2]==0 & drop[,3]==0)
        drop <- drop[!mono,]

        stat <- quantile(HWChisqStats(drop, pvalues = TRUE),
             c(0, 0.001, 0.01, 0.05, 0.25, 0.5, 0.75, 1))

     write.table(stat, file=paste0("./output/sim", chrom, "/chr", chrom, ".haldaneSim_", seed,
".txt"),
                 sep="\t", row.names=FALSE, quote=FALSE, col.names=FALSE)


## LD PRUNING ------------------------------------------------------------------------
## Input: VCF with imputed genotypes filtered for low MAF, deviation from HWE,
## and low imputation quality. Filters were applied using bcftools v.1.3.
## Requirements: plink v.1.90

## First convert to plink BED format
plink --vcf imputedGenos_filt.vcf \
         --make-bed \
         --mouse \
         --keep-allele-order \
         --out imputedGenos_filt

## this command produces two text files; one lists SNPs that were retained
## and one lists the SNPs that were pruned out
plink --bfile imputedGenos_filt \
         --indep-pairphase 1 kb 10 0.95 \
         --keep-allele-order \
         --mouse \
         --out imputedGenps_filt_LD

## use bcftools to filter SNPs from the input VCF
bcftools view \
              --o imputedGenos_filt_pruned \
              --R imputedGenps_filt_LD.prune.in \
             imputedGenos_filt.vcf

## convert to dosage format for GEMMA using 'chr.bp' in the SNP ID field
bcftools query \
              --f '%CHROM\.%POS %REF %ALT[ %DS]\n'
            --o imputedGenos_filt_pruned.dosage
            imputedGenos_filt.vcf
bcftools query \
              --f '%CHROM\.%POS %POS %CHROM\n'
            --o imputedGenos_filt_pruned.snpinfo
             imputedGenos_filt.vcf
```

```
## QTL/eQTL MAPPING ------------------------------------------------------------------------

## Make GRM
## Input: QC-filtered, LD-pruned file with genotype dosages from all chromosomes but one
## Requirements: gemma v.0.94
## dummy.pheno.txt contains a single column of 1s with 1063 rows; no header.
gemma -g chrNot7.dosage \
      -p dummy.pheno.txt \
      -gk 1 \
      -o chrNot7.kinship

## Map QTLs or eQTLs using a LOCO-LMM
## Input: leave-one-out GRMs, genotype dosages, phenotypes, covariates
## Requirements: gemma v.0.94
gemma -g chr7.dosage \
      -p phenos.allgeno \
      -k chrNot7.kinship.cXX.txt \
      -a chr7.snpinfo \
      -c trait.covs \
      -o trait.chr7.gwas \
      -lmm 2 \
      -n 5  # column number for trait of interest

## Heritability
## Input: GRM for all autosomal SNPs, phenotypes, covariates
## Requirements: gemma v.0.95alpha
## vc option 2 is the REML model (estimates are not constrained to be positive)
## remove -noconstrain argument to constrain estimates to be positive.
## to calculate pedigree-based heritability, replace the GRM (-k) with a kinship
## matrix calculated from the AIL pedigree (Supplementary Data 6)
gemma -p phenos.allgeno \
        -k chrAuto.refFiltFinal.kinship.cXX.txt \
      -c trait.covs \
      -o vg.trait.chr7 \
      -vc 2 \
      -noconstrain \
      -n 5  # column number for trait of interest
```

Supplementary References

1. Tzschentke, T. M. Measuring reward with the conditioned place preference (CPP) paradigm: update of the last decade. *Addict. Biol.* **12,** 227–462 (2007).
2. Hart, A. B., de Wit, H. & Palmer, A. A. Genetic factors modulating the response to stimulant drugs in humans. *Curr. Top. Behav. Neurosci.* **12,** 537–577 (2012).
3. de Wit, H., Uhlenhuth, E. H. & Johanson, C. E. Individual differences in the reinforcing and subjective effects of amphetamine and diazepam. *Drug Alcohol Depend.* **16,** 341–360 (1986).
4. Mayo, L. M. *et al.* Conditioned preference to a methamphetamine-associated contextual cue in humans. *Neuropsychopharmacol. Off. Publ. Am. Coll. Neuropsychopharmacol.* **38,** 921–929 (2013).
5. Niwa, M., Yan, Y. & Nabeshima, T. Genes and molecules that can potentiate or attenuate psychostimulant dependence: relevance of data from animal models to human addiction. *Ann. N. Y. Acad. Sci.* **1141,** 76–95 (2008).
6. Uhl, G. R., Drgon, T., Johnson, C. & Liu, Q.-R. Addiction genetics and pleiotropic effects of common haplotypes that make polygenic contributions to vulnerability to substance dependence. *J. Neurogenet.* **23,** 272–282 (2009).
7. Li, C.-Y. *et al.* Meta-analysis and genome-wide interpretation of genetic susceptibility to drug addiction. *BMC Genomics* **12,** 508 (2011).
8. Scherrer, J. F. *et al.* Subjective effects to cannabis are associated with use, abuse and dependence after adjusting for genetic and environmental influences. *Drug Alcohol Depend.* **105,** 76–82 (2009).
9. de Wit, H. & Phillips, T. J. Do initial responses to drugs predict future use or abuse? *Neurosci. Biobehav. Rev.* **36,** 1565–1576 (2012).
10. Shabani, S., McKinnon, C. S., Reed, C., Cunningham, C. L. & Phillips, T. J. Sensitivity to rewarding or aversive effects of methamphetamine determines methamphetamine intake. *Genes Brain Behav.* **10,** 625–636 (2011).
11. Collier, A. D., Khan, K. M., Caramillo, E. M., Mohn, R. S. & Echevarria, D. J. Zebrafish and conditioned place preference: a translational model of drug reward. *Prog. Neuropsychopharmacol. Biol. Psychiatry* **55,** 16–25 (2014).
12. Imeh-Nathaniel, A., Adedeji, A., Huber, R. & Nathaniel, T. I. The rewarding properties of methamphetamine in an invertebrate model of drug addiction. *Physiol. Behav.* **153,** 40–46 (2016).
13. Liberzon, A. *et al.* The Molecular Signatures Database (MSigDB) hallmark gene set collection. *Cell Syst.* **1,** 417–425 (2015).
14. Cavallo, J. S., Mayo, L. M. & de Wit, H. Acquisition of Conditioning between Methamphetamine and Cues in Healthy Humans. *PloS One* **11,** e0161541 (2016).
15. Childs, E. & de Wit, H. Contextual conditioning enhances the psychostimulant and incentive properties of d-amphetamine in humans. *Addict. Biol.* **18,** 985–992 (2013).
16. Childs, E. & de Wit, H. Amphetamine-induced place preference in humans. *Biol. Psychiatry* **65,** 900–904 (2009).
17. Hogarth, L., Balleine, B. W., Corbit, L. H. & Killcross, S. Associative learning mechanisms underpinning the transition from recreational drug use to addiction. *Ann. N. Y. Acad. Sci.* **1282,** 12–24 (2013).
18. Bryant, C. D., Kole, L. A., Guido, M. A., Cheng, R. & Palmer, A. A. Methamphetamine-induced conditioned place preference in LG/J and SM/J mouse strains and an F45/F46 advanced intercross line. *Front. Genet.* **3,** 126 (2012).
19. Heidbreder, C. Advances in animal models of drug addiction. *Curr. Top. Behav. Neurosci.* **7,** 213–250 (2011).
20. Vezina, P. & Leyton, M. Conditioned cues and the expression of stimulant sensitization in animals and humans. *Neuropharmacology* **56 Suppl 1,** 160–168 (2009).
21. Graham, F. K. Presidential Address, 1974. The more or less startling effects of weak prestimulation. *Psychophysiology* **12,** 238–248 (1975).
22. Swerdlow, N. R., Braff, D. L. & Geyer, M. A. Sensorimotor gating of the startle reflex: what we said 25 years ago, what has happened since then, and what comes next. *J. Psychopharmacol. Oxf. Engl.* **30,** 1072–1081 (2016).

23. Samocha, K. E., Lim, J. E., Cheng, R., Sokoloff, G. & Palmer, A. A. Fine mapping of QTL for prepulse inhibition in LG/J and SM/J mice using F(2) and advanced intercross lines. *Genes Brain Behav.* **9,** 759–767 (2010).
24. Palmer, A. A. *et al.* Identification of quantitative trait loci for prepulse inhibition in rats. *Psychopharmacology (Berl.)* **165,** 270–279 (2003).
25. Swerdlow, N. R. *et al.* Deficient prepulse inhibition in schizophrenia in a multi-site cohort: Internal replication and extension. *Schizophr. Res.* (2017). doi:10.1016/j.schres.2017.05.013
26. Parker, C. C. *et al.* Genome-wide association study of behavioral, physiological and gene expression traits in outbred CFW mice. *Nat. Genet.* **48,** 919–926 (2016).
27. Hrbek, T., de Brito, R. A., Wang, B., Pletscher, L. S. & Cheverud, J. M. Genetic characterization of a new set of recombinant inbred lines (LGXSM) formed from the inter-cross of SM/J and LG/J inbred mouse strains. *Mamm. Genome Off. J. Int. Mamm. Genome Soc.* **17,** 417–429 (2006).
28. Zhou, X., Carbonetto, P. & Stephens, M. Polygenic modeling with bayesian sparse linear mixed models. *PLoS Genet.* **9,** e1003264 (2013).
29. Wahlsten, D., Metten, P. & Crabbe, J. C. A rating scale for wildness and ease of handling laboratory mice: results for 21 inbred strains tested in two laboratories. *Genes Brain Behav.* **2,** 71–79 (2003).
30. Bloemberg, D. & Quadrilatero, J. Rapid determination of myosin heavy chain expression in rat, mouse, and human skeletal muscle using multicolor immunofluorescence analysis. *PloS One* **7,** e35273 (2012).
31. Messina, G. & Cossu, G. The origin of embryonic and fetal myoblasts: a role of Pax3 and Pax7. *Genes Dev.* **23,** 902–905 (2009).
32. Cheng, R. *et al.* Genome-wide association studies and the problem of relatedness among advanced intercross lines and other highly recombinant populations. *Genetics* **185,** 1033–1044 (2010).
33. Toker, L., Feng, M. & Pavlidis, P. Whose sample is it anyway? Widespread misannotation of samples in transcriptomics studies. *F1000Research* **5,** 2103 (2016).
34. Han, L. & Abney, M. Identity by descent estimation with dense genome-wide genotype data. *Genet. Epidemiol.* **35,** 557–567 (2011).
35. Abney, M. Identity-by-descent estimation and mapping of qualitative traits in large, complex pedigrees. *Genetics* **179,** 1577–1590 (2008).
36. Korneliussen, T. S., Albrechtsen, A. & Nielsen, R. ANGSD: Analysis of Next Generation Sequencing Data. *BMC Bioinformatics* **15,** 356 (2014).
37. Browning, S. R. & Browning, B. L. Rapid and Accurate Haplotype Phasing and Missing-Data Inference for Whole-Genome Association Studies By Use of Localized Haplotype Clustering. *Am. J. Hum. Genet.* **81,** 1084–1097 (2007).
38. Morgan, A. P. *et al.* The Mouse Universal Genotyping Array: From Substrains to Subspecies. *G3 Bethesda Md* **6,** 263–279 (2015).
39. Nikolskiy, I. *et al.* Using whole-genome sequences of the LG/J and SM/J inbred mouse strains to prioritize quantitative trait genes and nucleotides. *BMC Genomics* **16,** 415 (2015).
40. Bimber, B. N. *et al.* Whole-genome characterization in pedigreed non-human primates using genotyping-by-sequencing (GBS) and imputation. *BMC Genomics* **17,** (2016).
41. Marchini, J. & Howie, B. Genotype imputation for genome-wide association studies. *Nat. Rev. Genet.* **11,** 499–511 (2010).
42. Huang, L., Wang, C. & Rosenberg, N. A. The relationship between imputation error and statistical power in genetic association studies in diverse populations. *Am. J. Hum. Genet.* **85,** 692–698 (2009).
43. Li, H. A statistical framework for SNP calling, mutation discovery, association mapping and population genetical parameter estimation from sequencing data. *Bioinforma. Oxf. Engl.* **27,** 2987–2993 (2011).
44. Elshire, R. J. *et al.* A robust, simple genotyping-by-sequencing (GBS) approach for high diversity species. *PloS One* **6,** e19379 (2011).
45. Browning, B. L. & Browning, S. R. Genotype Imputation with Millions of Reference Samples. *Am. J. Hum. Genet.* **98,** 116–126 (2016).
46. Cox, A. *et al.* A new standard genetic map for the laboratory mouse. *Genetics* **182,** 1335–1344 (2009).
47. Cheng, R., Abney, M., Palmer, A. A. & Skol, A. D. QTLRel: an R package for genome-wide association studies in which relatedness is a concern. *BMC Genet.* **12,** 66 (2011).

48. Graffelman, J. Exploring Diallelic genetic markers: The Hardy Weinberg package. *J. Stat. Softw.* **64,** 1–23 (2015).
49. Chang, C. C. *et al.* Second-generation PLINK: rising to the challenge of larger and richer datasets. *GigaScience* **4,** 7 (2015).
50. Cheng, R., Parker, C. C., Abney, M. & Palmer, A. A. Practical considerations regarding the use of genotype and pedigree data to model relatedness in the context of genome-wide association studies. *G3 Bethesda Md* **3,** 1861–1867 (2013).
51. Abney, M. Permutation testing in the presence of polygenic variation. *Genet. Epidemiol.* **39,** 249–258 (2015).
52. Joo, J. W. J., Hormozdiari, F., Han, B. & Eskin, E. Multiple testing correction in linear mixed models. *Genome Biol.* **17,** 62 (2016).
53. Han, B., Kang, H. M. & Eskin, E. Rapid and accurate multiple testing correction and power estimation for millions of correlated markers. *PLoS Genet.* **5,** e1000456 (2009).
54. Diaz-Garcia, L., Covarrubias-Pazaran, G., Schlautman, B. & Zalapa, J. SOFIA: an R package for enhancing genetic visualization with Circos. *bioRxiv* 088377 (2016). doi:10.1101/088377
55. Anders, S. & Huber, W. Differential expression analysis for sequence count data. *Genome Biol.* **11,** R106 (2010).
56. Lawrence, M. *et al.* Software for computing and annotating genomic ranges. *PLoS Comput. Biol.* **9,** e1003118 (2013).
57. Wickham, H. Ggplot2 : elegant graphics for data analysis. (2009). Available at: http://public.eblib.com/choice/publicfullrecord.aspx?p=511468.
58. Chen, H. & Boutros, P. C. VennDiagram: a package for the generation of highly-customizable Venn and Euler diagrams in R. *BMC Bioinformatics* **12,** 35 (2011).
59. Li, H. & Durbin, R. Fast and accurate long-read alignment with Burrows-Wheeler transform. *Bioinforma. Oxf. Engl.* **26,** 589–595 (2010).
60. DePristo, M. A. *et al.* A framework for variation discovery and genotyping using next-generation DNA sequencing data. *Nat. Genet.* **43,** 491–498 (2011).
61. Zhou, X. A unifed framework for variance component estimation with summary statistics in genome-wide association studies. *Ann. Appl. Stat.* **In press,** (2017).
62. Zhou, X. & Stephens, M. Genome-wide efficient mixed-model analysis for association studies. *Nat. Genet.* **44,** 821–824 (2012).
63. Kim, D., Langmead, B. & Salzberg, S. L. HISAT: a fast spliced aligner with low memory requirements. *Nat. Methods* **12,** 357–360 (2015).
64. Krzywinski, M. *et al.* Circos: an information aesthetic for comparative genomics. *Genome Res.* **19,** 1639–1645 (2009).
65. Li, H. *et al.* The Sequence Alignment/Map format and SAMtools. *Bioinforma. Oxf. Engl.* **25,** 2078–2079 (2009).

Supplementary Figures 1-24

Supplementary Figures 1-24, listed below, are provided on the following pages.

Supplementary Figure 1: SNPs obtained using GBS and GigaMUGA
Supplementary Figure 2: Manhattan plots
Supplementary Figure 3: MAF and locus effect size in G50-56 of the LGxSM AIL as compared to CFW mice
Supplementary Figure 4: Trait correlation heat maps
Supplementary Figure 5: Genetic and environmental correlations between traits
Supplementary Figure 6: Summary of eQTLs by brain region
Supplementary Figure 7: *cis*-eQTLs and *trans*-eQTLs in HIP, PFC and STR
Supplementary Figure 8: Quantile-quantile plot of *trans*-eQTLs in HIP, PFC and STR
Supplementary Figure 9: Master eQTLs in HIP, PFC and STR
Supplementary Figure 10: A locus strongly associated with startle on chromosome 17 has pleiotropic effects on behavior
Supplementary Figure 11: Average *Csmd1* read coverage for SM and LG homozygotes in HIP, PFC and STR
Supplementary Figure 12: Several *trans*-eQTLs located in *Itpr1* overlap a locus associated with D1 side changes on chromosome 6
Supplementary Figure 13: Pleiotropic effects of a locus associated with body weight on chromosome 2
Supplementary Figure 14: A locus on chromosome 7 has pleiotropic effects on body weight and TA mass
Supplementary Figure 15: A strong association with EDL mass on chromosome 13
Supplementary Figure 16: Pleiotropic effects on physiology and behavior at a locus on chromosome 4
Supplementary Figure 17: Pleiotropic effects on gastrocnemius weight and locomotor activity at a locus on chromosome 4
Supplementary Figure 18: Pleiotropic effects of a locus associated with locomotor activity on chromosome 12
Supplementary Figure 19: Sample size needed for 80% power to detect associations with effect sizes ranging from 0.01 to 0.05
Supplementary Figure 20: Identification of outliers for startle and PPI
Supplementary Figure 21: Primary alignment rate and number of reads in GBS samples
Supplementary Figure 22: Primary alignment rate and number of reads in RNA-seq samples
Supplementary Figure 23: Principal components analysis of RNA-seq data after correcting sample mix-ups
Supplementary Figure 24: Principal components of gene expression data for HIP, STR and PFC

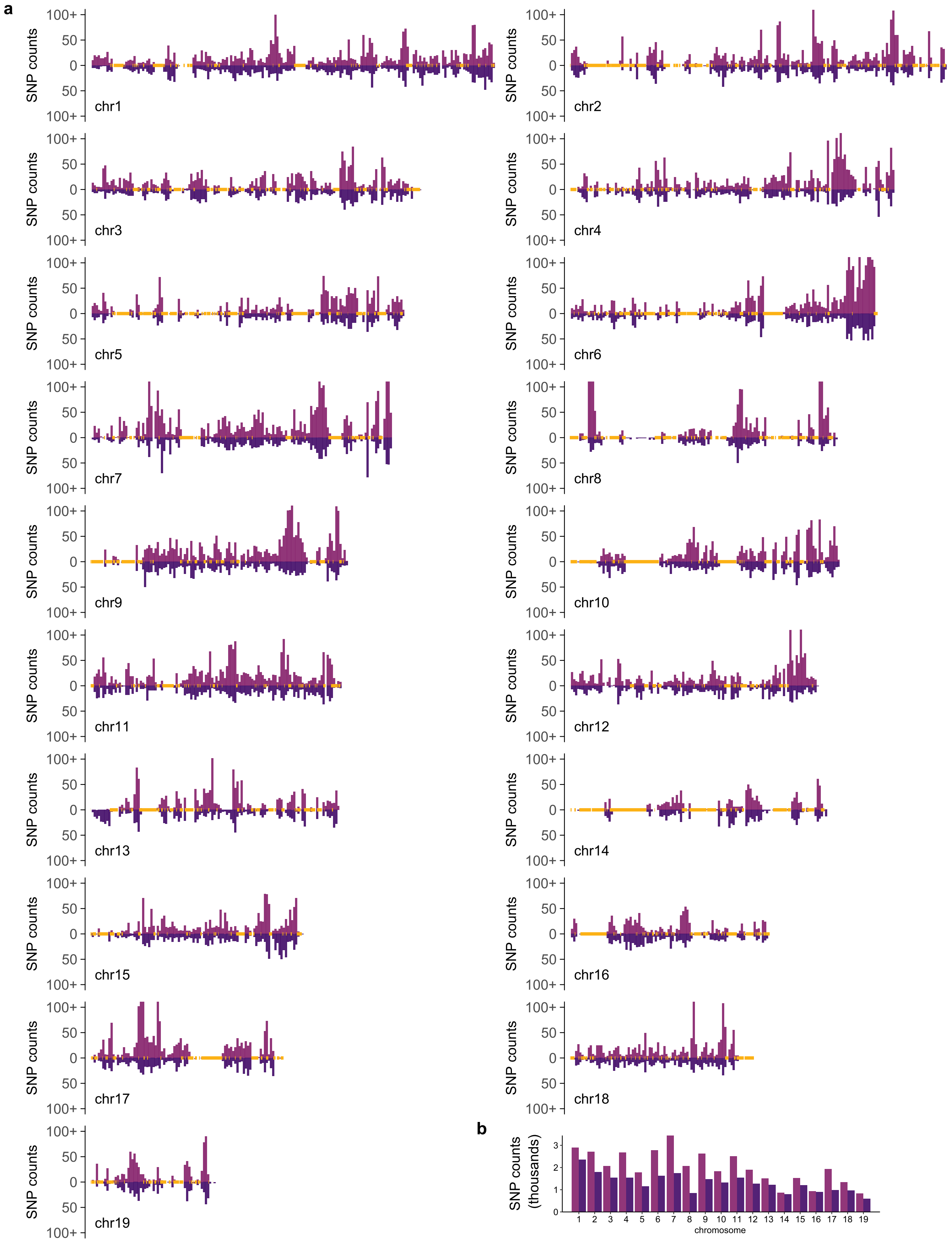

Figure 1: **SNPs obtained using GBS and GigaMUGA. (a)** Histograms showing the distribution of GBS SNPs before reference panel imputation (top, light purple), and GigaMUGA SNPs (bottom, dark purple) that overlap known SNPs segregating in LG and SM. SNPs are plotted in 1 Mb bins for each autosome. At the x-axes, regions predicted by Nikolskiy *et al.* to be nearly IBD in LG and SM are marked in gold. **(b)** Histogram showing the total number of known SNPs segregating in LG and SM that were captured using GBS before reference panel imputation (light purple) and GigaMUGA (dark purple).

Figure 2: **Manhattan plots.** **(a-r)** Manhattan plots are grouped by trait. The dashed line in each panel indicates a permutation-derived significance threshold of $p = 8.06 \times 10^{-6} (\alpha = 0.05)$. The top SNP for each locus is marked and labeled with its rsid (dbSNP v.142). SNP heritability (proportion of variance explained by 523,028 SNPs used for GWAS) and standard error are shown in the upper right corner of each panel. **(a)** body weight measured on D1-D8 of the CPP test. **(b)** body weight measured after the CPP test. **(c)** hind limb muscle weights. **(d)** startle and habituation. **(e)** PPI (prepulse intensities are expressed in absolute terms (e.g. 82 dB = 12 dB over the 70 dB background). **(f)** CPP on D8 (number of seconds spent on the methamphetamine-paired side of the testing chamber after conditioning). **(g)** CPP on D1 (number of seconds spent on the methamphetamine-paired side of the testing chamber before conditioning). **(h)** CPP defined as the difference in CPP between D8 and D1 (D8-D1). **(i)** methamphetamine-induced activity (distance traveled) on D2. **(j)** methamphetamine-induced activity (distance traveled) on D4. **(k)** locomotor sensitization to methamphetamine (distance traveled on D4 D2). **(l)** saline-induced activity (side changes) on D1. **(m)** saline-induced activity (side changes) on D8. **(n)** saline-induced activity (distance traveled) on D3. **(o)** saline-induced activity (distance traveled) on D5. **(p)** saline-induced activity (distance traveled) on D1. **(q)** saline-induced activity (distance traveled) on D8. **(r)** fasting glucose levels, bone length and wildness.

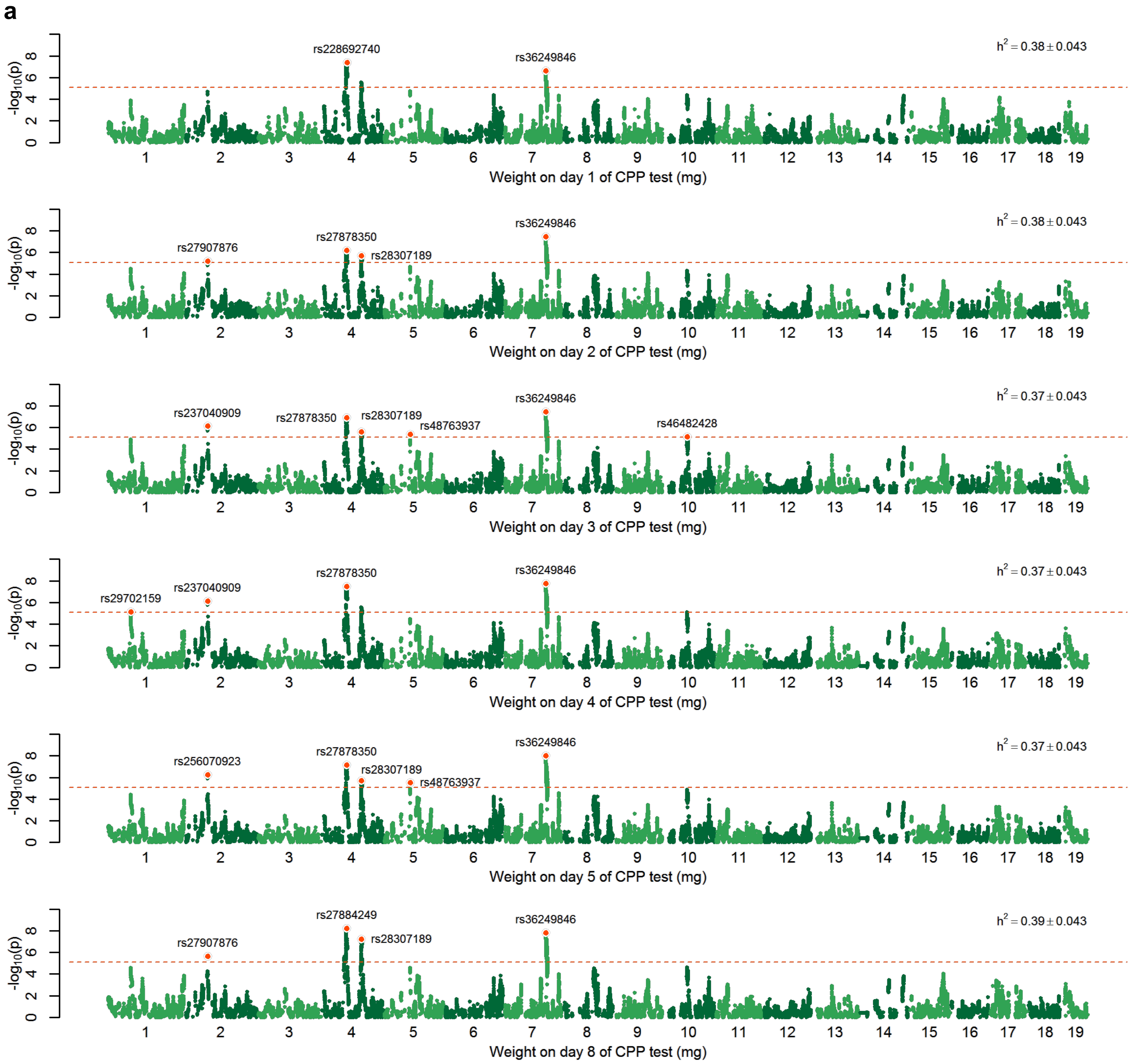
a
rs228692740
rs36249846
h2 = 0.38 ± 0.043
-log10(p)
Weight on day 1 of CPP test (mg)
rs27907876
rs27878350
rs28307189
rs36249846
h2 = 0.38 ± 0.043
Weight on day 2 of CPP test (mg)
rs237040909
rs27878350
rs28307189
rs48763937
rs36249846
rs46482428
h2 = 0.37 ± 0.043
Weight on day 3 of CPP test (mg)
rs29702159
rs237040909
rs27878350
rs36249846
h2 = 0.37 ± 0.043
Weight on day 4 of CPP test (mg)
rs256070923
rs27878350
rs28307189
rs48763937
rs36249846
h2 = 0.37 ± 0.043
Weight on day 5 of CPP test (mg)
rs27907876
rs27884249
rs28307189
rs36249846
h2 = 0.39 ± 0.043
Weight on day 8 of CPP test (mg)

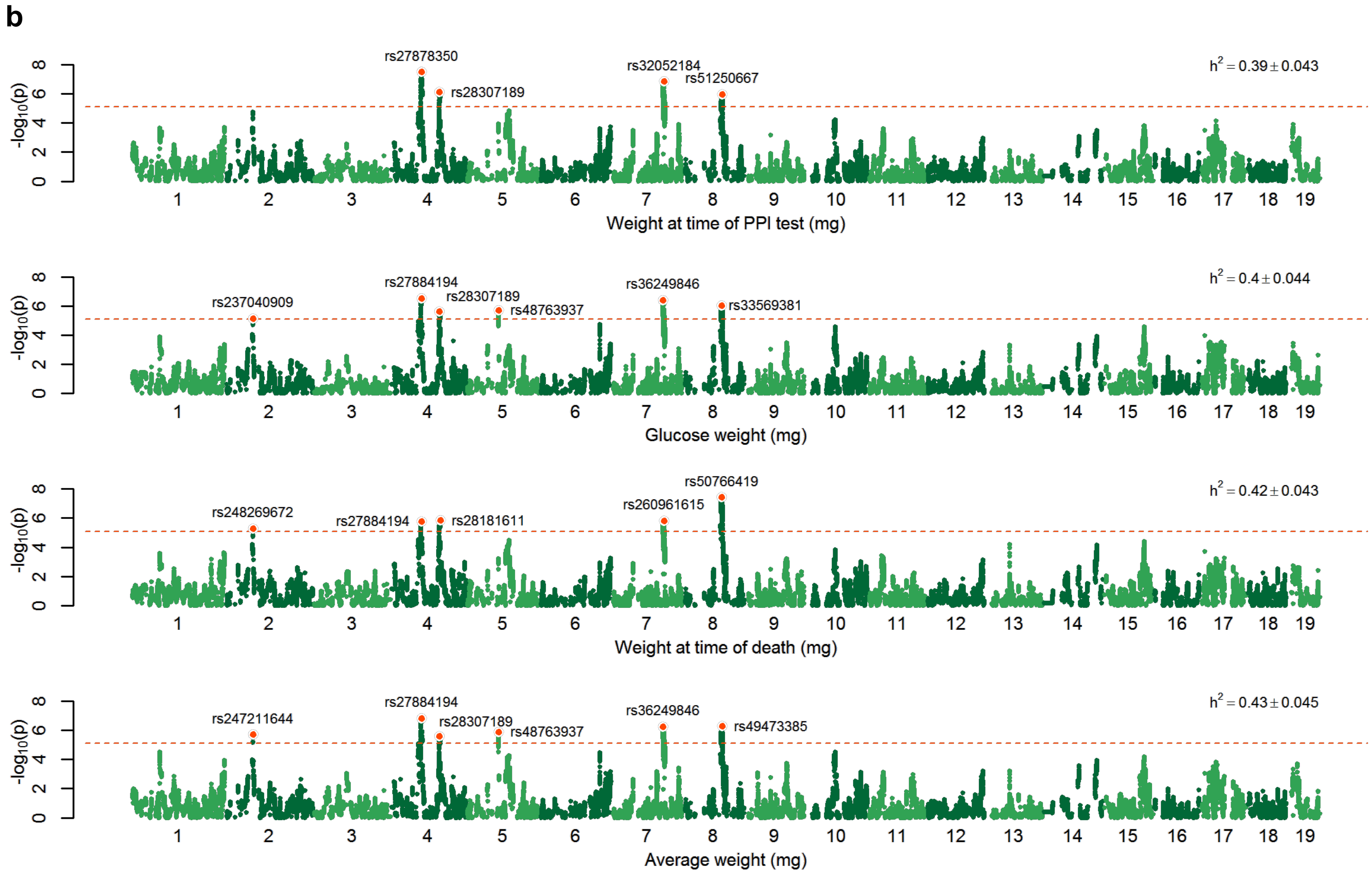
b
rs27878350
rs28307189
rs32052184
rs51250667
h² = 0.39 ± 0.043
-log10(p)
Weight at time of PPI test (mg)
rs237040909
rs27884194
rs28307189
rs48763937
rs36249846
rs33569381
h² = 0.4 ± 0.044
Glucose weight (mg)
rs248269672
rs27884194
rs28181611
rs260961615
rs50766419
h² = 0.42 ± 0.043
Weight at time of death (mg)
rs247211644
rs27884194
rs28307189
rs48763937
rs36249846
rs49473385
h² = 0.43 ± 0.045
Average weight (mg)
1 2 3 4 5 6 7 8 9 10 11 12 13 14 15 16 17 18 19

c

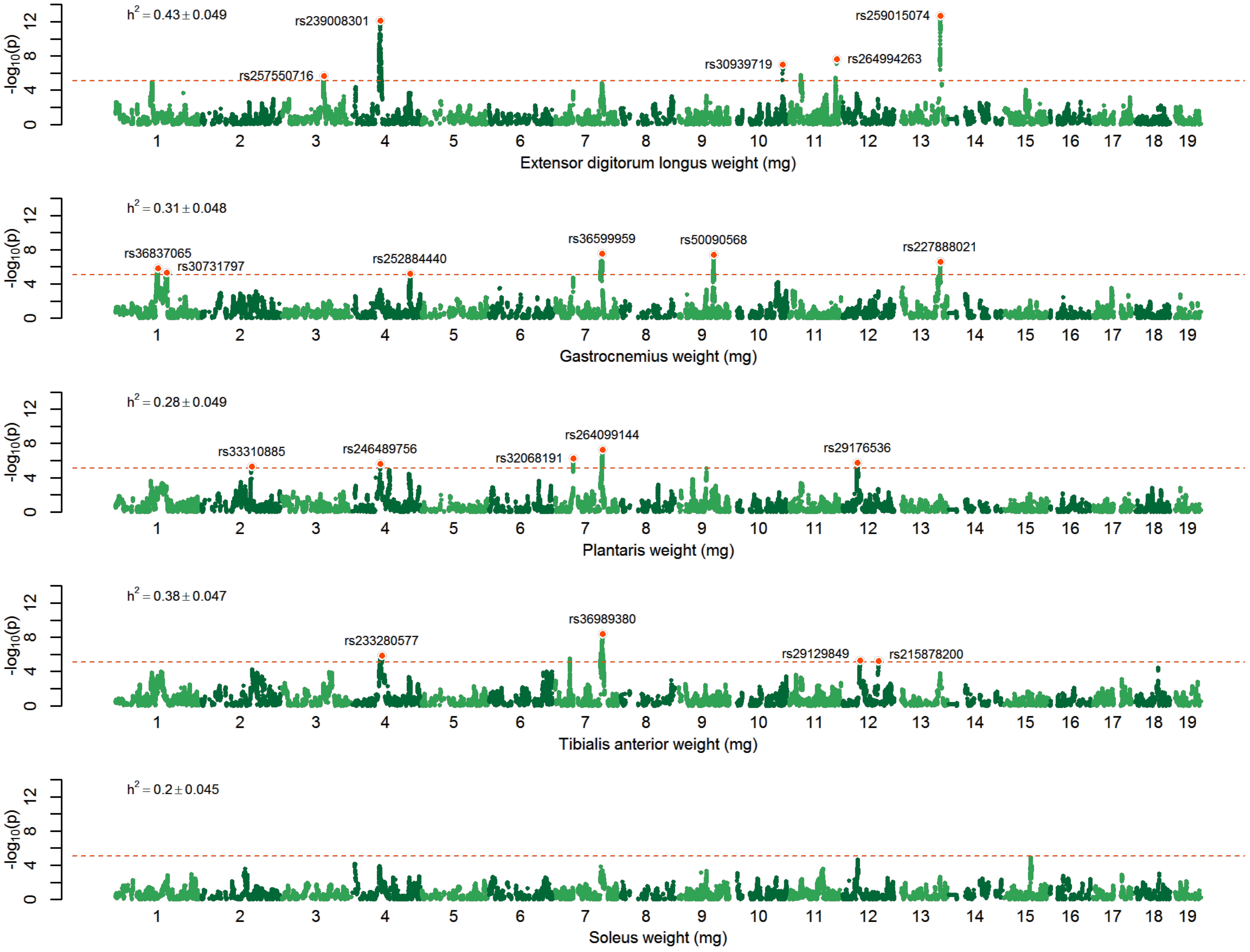
h2 = 0.43 ± 0.049
rs239008301
rs257550716
rs30939719
rs264994263
rs259015074
Extensor digitorum longus weight (mg)
h2 = 0.31 ± 0.048
rs36837065
rs30731797
rs252884440
rs36599959
rs50090568
rs227888021
Gastrocnemius weight (mg)
h2 = 0.28 ± 0.049
rs33310885
rs246489756
rs32068191
rs264099144
rs29176536
Plantaris weight (mg)
h2 = 0.38 ± 0.047
rs233280577
rs36989380
rs29129849
rs215878200
Tibialis anterior weight (mg)
h2 = 0.2 ± 0.045
Soleus weight (mg)
-log10(p)
1 2 3 4 5 6 7 8 9 10 11 12 13 14 15 16 17 18 19

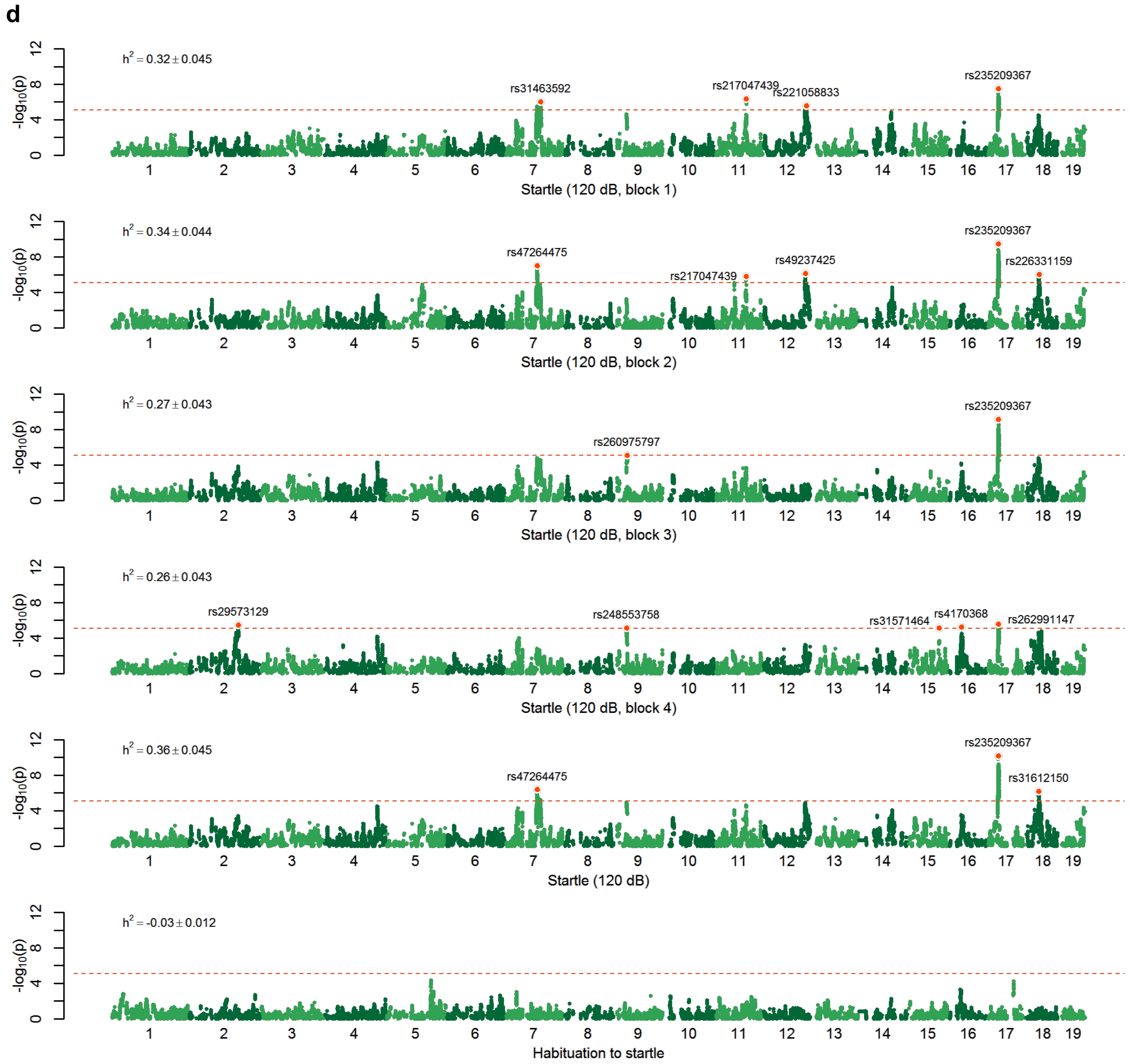
d
h² = 0.32 ± 0.045
rs31463592
rs217047439
rs221058833
rs235209367
Startle (120 dB, block 1)
h² = 0.34 ± 0.044
rs47264475
rs217047439
rs49237425
rs235209367
rs226331159
Startle (120 dB, block 2)
h² = 0.27 ± 0.043
rs260975797
rs235209367
Startle (120 dB, block 3)
h² = 0.26 ± 0.043
rs29573129
rs248553758
rs31571464
rs4170368
rs262991147
Startle (120 dB, block 4)
h² = 0.36 ± 0.045
rs47264475
rs235209367
rs31612150
Startle (120 dB)
h² = -0.03 ± 0.012
Habituation to startle
-log10(p)
1 2 3 4 5 6 7 8 9 10 11 12 13 14 15 16 17 18 19

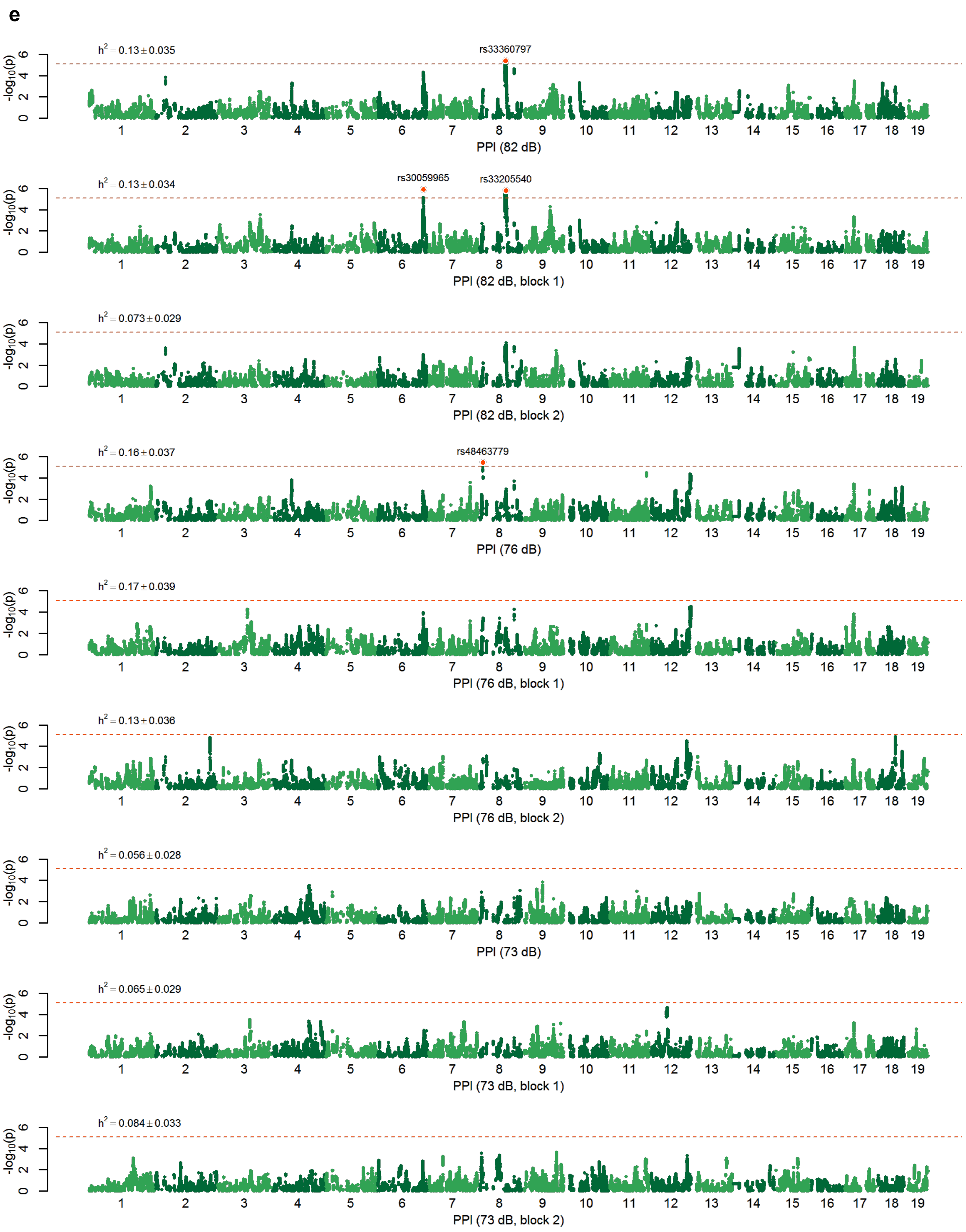
e
h2 = 0.13 ± 0.035
rs33360797
-log10(p)
PPI (82 dB)
h2 = 0.13 ± 0.034
rs30059965
rs33205540
PPI (82 dB, block 1)
h2 = 0.073 ± 0.029
PPI (82 dB, block 2)
h2 = 0.16 ± 0.037
rs48463779
PPI (76 dB)
h2 = 0.17 ± 0.039
PPI (76 dB, block 1)
h2 = 0.13 ± 0.036
PPI (76 dB, block 2)
h2 = 0.056 ± 0.028
PPI (73 dB)
h2 = 0.065 ± 0.029
PPI (73 dB, block 1)
h2 = 0.084 ± 0.033
PPI (73 dB, block 2)

g

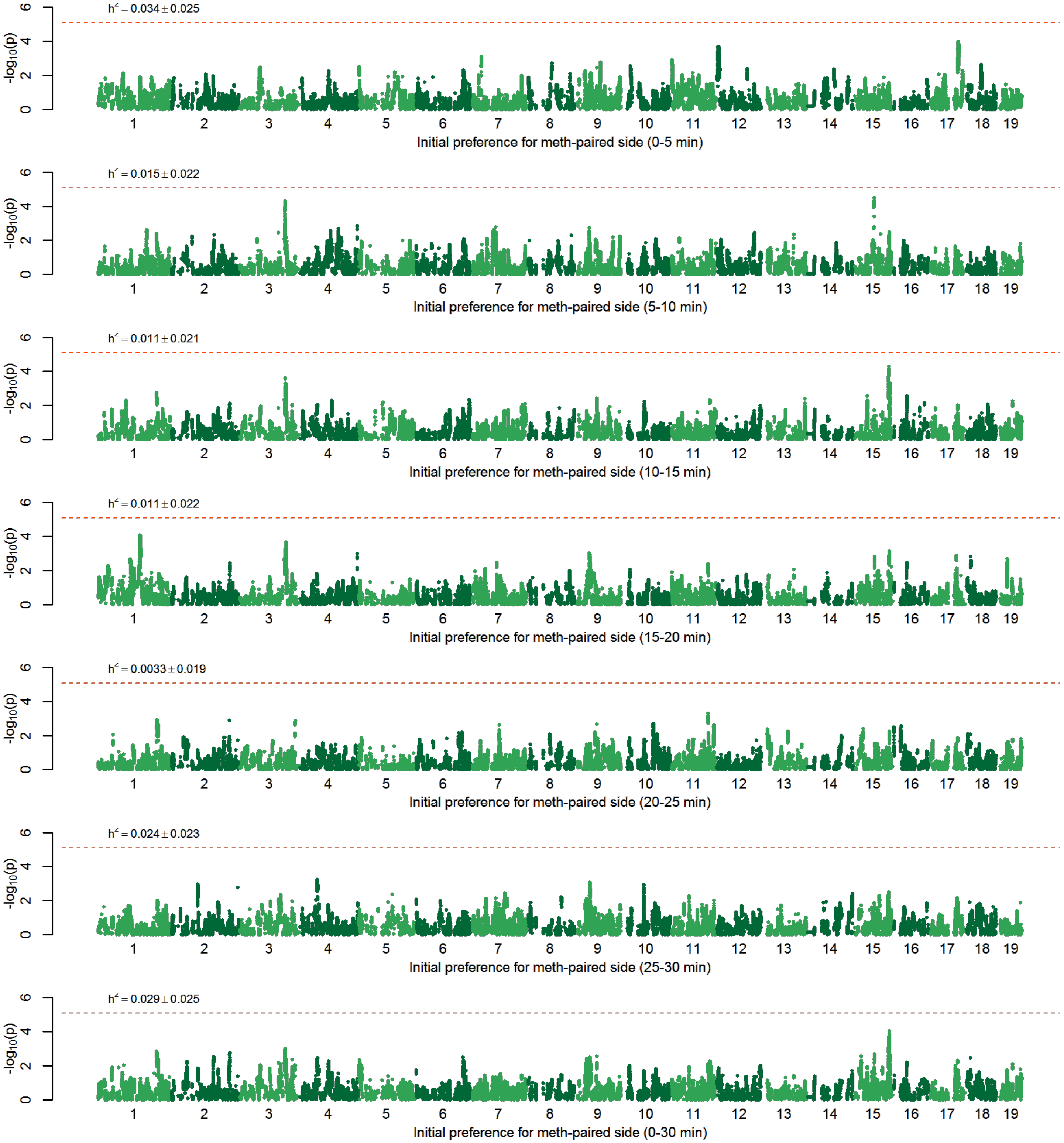

$h^2 = 0.034 \pm 0.025$
-log10(p)
Initial preference for meth-paired side (0-5 min)
$h^2 = 0.015 \pm 0.022$
Initial preference for meth-paired side (5-10 min)
$h^2 = 0.011 \pm 0.021$
Initial preference for meth-paired side (10-15 min)
$h^2 = 0.011 \pm 0.022$
Initial preference for meth-paired side (15-20 min)
$h^2 = 0.0033 \pm 0.019$
Initial preference for meth-paired side (20-25 min)
$h^2 = 0.024 \pm 0.023$
Initial preference for meth-paired side (25-30 min)
$h^2 = 0.029 \pm 0.025$
Initial preference for meth-paired side (0-30 min)

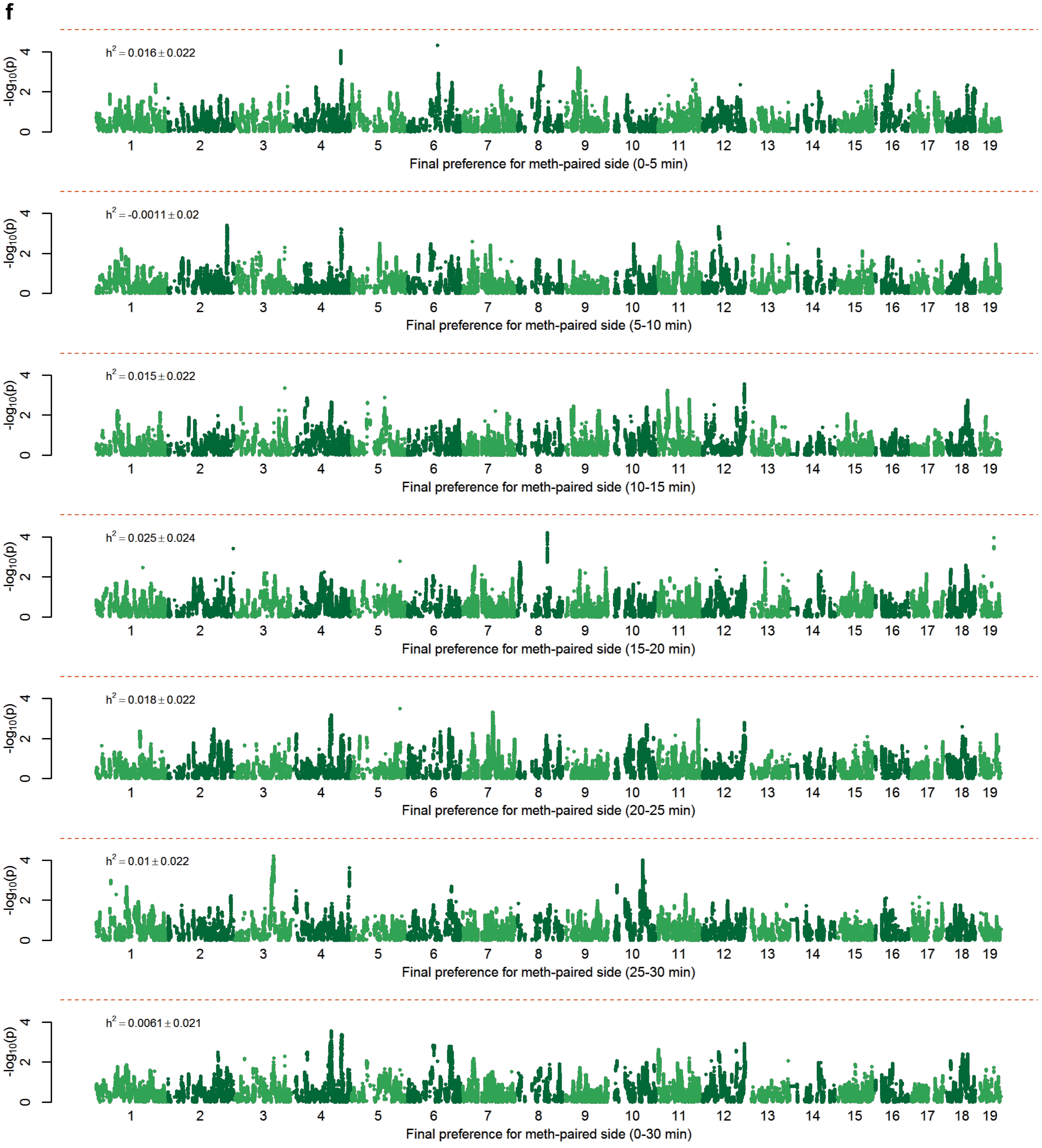
f
h² = 0.016 ± 0.022
Final preference for meth-paired side (0-5 min)
h² = -0.0011 ± 0.02
Final preference for meth-paired side (5-10 min)
h² = 0.015 ± 0.022
Final preference for meth-paired side (10-15 min)
h² = 0.025 ± 0.024
Final preference for meth-paired side (15-20 min)
h² = 0.018 ± 0.022
Final preference for meth-paired side (20-25 min)
h² = 0.01 ± 0.022
Final preference for meth-paired side (25-30 min)
h² = 0.0061 ± 0.021
Final preference for meth-paired side (0-30 min)
-log10(p)
0 2 4
1 2 3 4 5 6 7 8 9 10 11 12 13 14 15 16 17 18 19

h

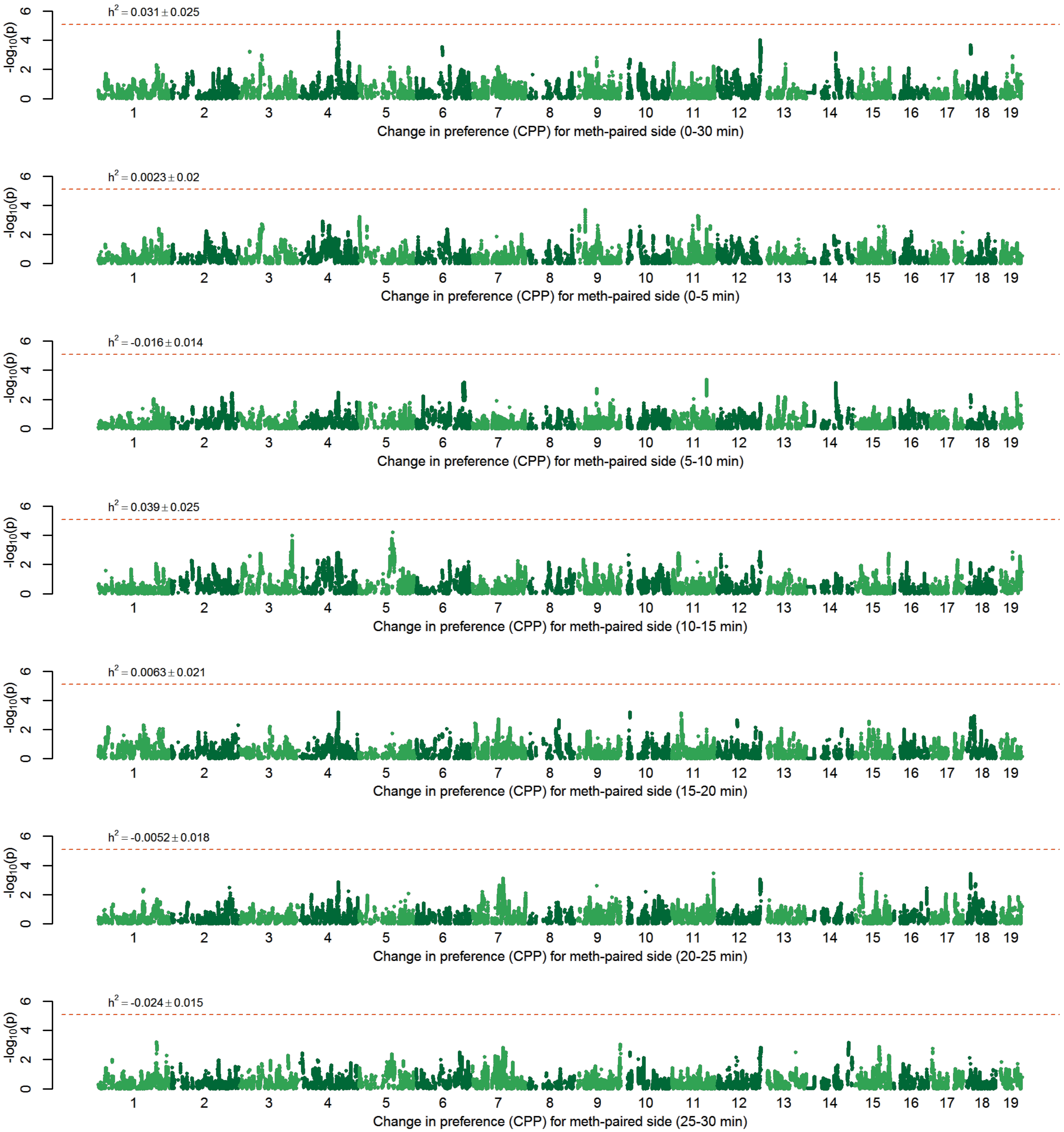
$h^2 = 0.031 \pm 0.025$
-log10(p)
Change in preference (CPP) for meth-paired side (0-30 min)
$h^2 = 0.0023 \pm 0.02$
-log10(p)
Change in preference (CPP) for meth-paired side (0-5 min)
$h^2 = -0.016 \pm 0.014$
-log10(p)
Change in preference (CPP) for meth-paired side (5-10 min)
$h^2 = 0.039 \pm 0.025$
-log10(p)
Change in preference (CPP) for meth-paired side (10-15 min)
$h^2 = 0.0063 \pm 0.021$
-log10(p)
Change in preference (CPP) for meth-paired side (15-20 min)
$h^2 = -0.0052 \pm 0.018$
-log10(p)
Change in preference (CPP) for meth-paired side (20-25 min)
$h^2 = -0.024 \pm 0.015$
-log10(p)
Change in preference (CPP) for meth-paired side (25-30 min)

i

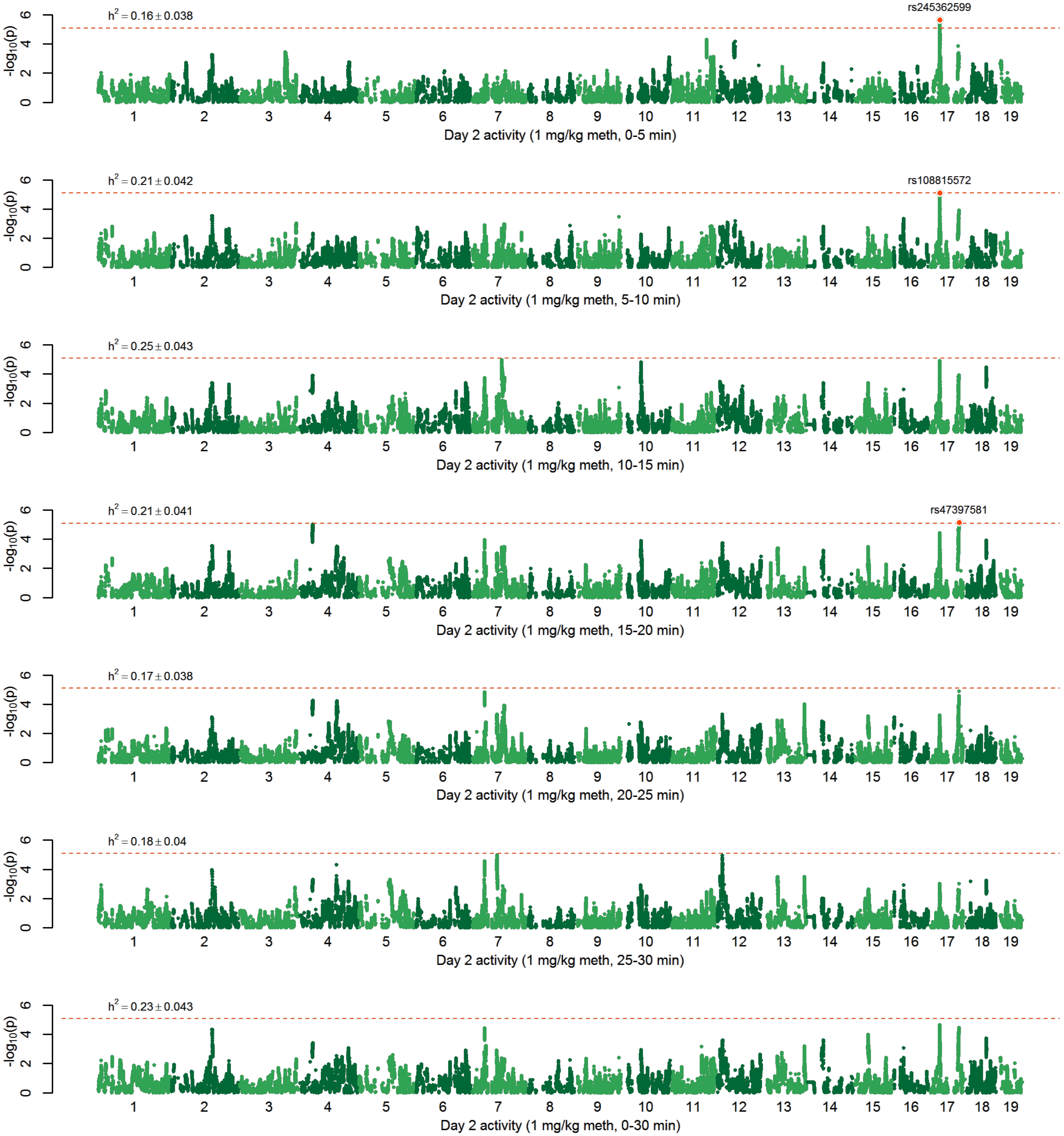
h2 = 0.16 ± 0.038
rs245362599
-log10(p)
Day 2 activity (1 mg/kg meth, 0-5 min)
h2 = 0.21 ± 0.042
rs108815572
Day 2 activity (1 mg/kg meth, 5-10 min)
h2 = 0.25 ± 0.043
Day 2 activity (1 mg/kg meth, 10-15 min)
h2 = 0.21 ± 0.041
rs47397581
Day 2 activity (1 mg/kg meth, 15-20 min)
h2 = 0.17 ± 0.038
Day 2 activity (1 mg/kg meth, 20-25 min)
h2 = 0.18 ± 0.04
Day 2 activity (1 mg/kg meth, 25-30 min)
h2 = 0.23 ± 0.043
Day 2 activity (1 mg/kg meth, 0-30 min)

j

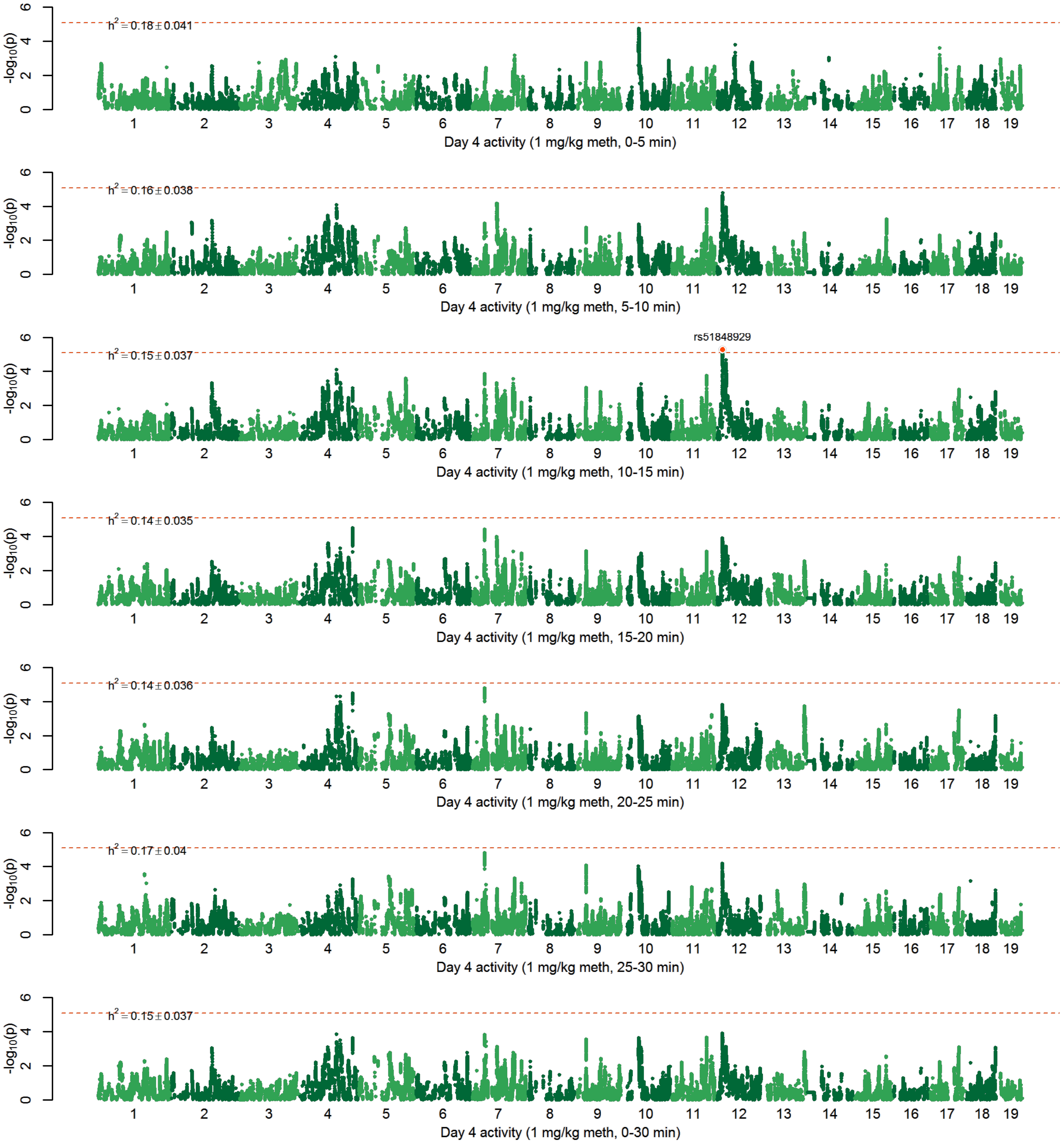
h2 = 0.18 ± 0.041
Day 4 activity (1 mg/kg meth, 0-5 min)
h2 = 0.16 ± 0.038
Day 4 activity (1 mg/kg meth, 5-10 min)
rs51848929
h2 = 0.15 ± 0.037
Day 4 activity (1 mg/kg meth, 10-15 min)
h2 = 0.14 ± 0.035
Day 4 activity (1 mg/kg meth, 15-20 min)
h2 = 0.14 ± 0.036
Day 4 activity (1 mg/kg meth, 20-25 min)
h2 = 0.17 ± 0.04
Day 4 activity (1 mg/kg meth, 25-30 min)
h2 = 0.15 ± 0.037
Day 4 activity (1 mg/kg meth, 0-30 min)
-log10(p)

k

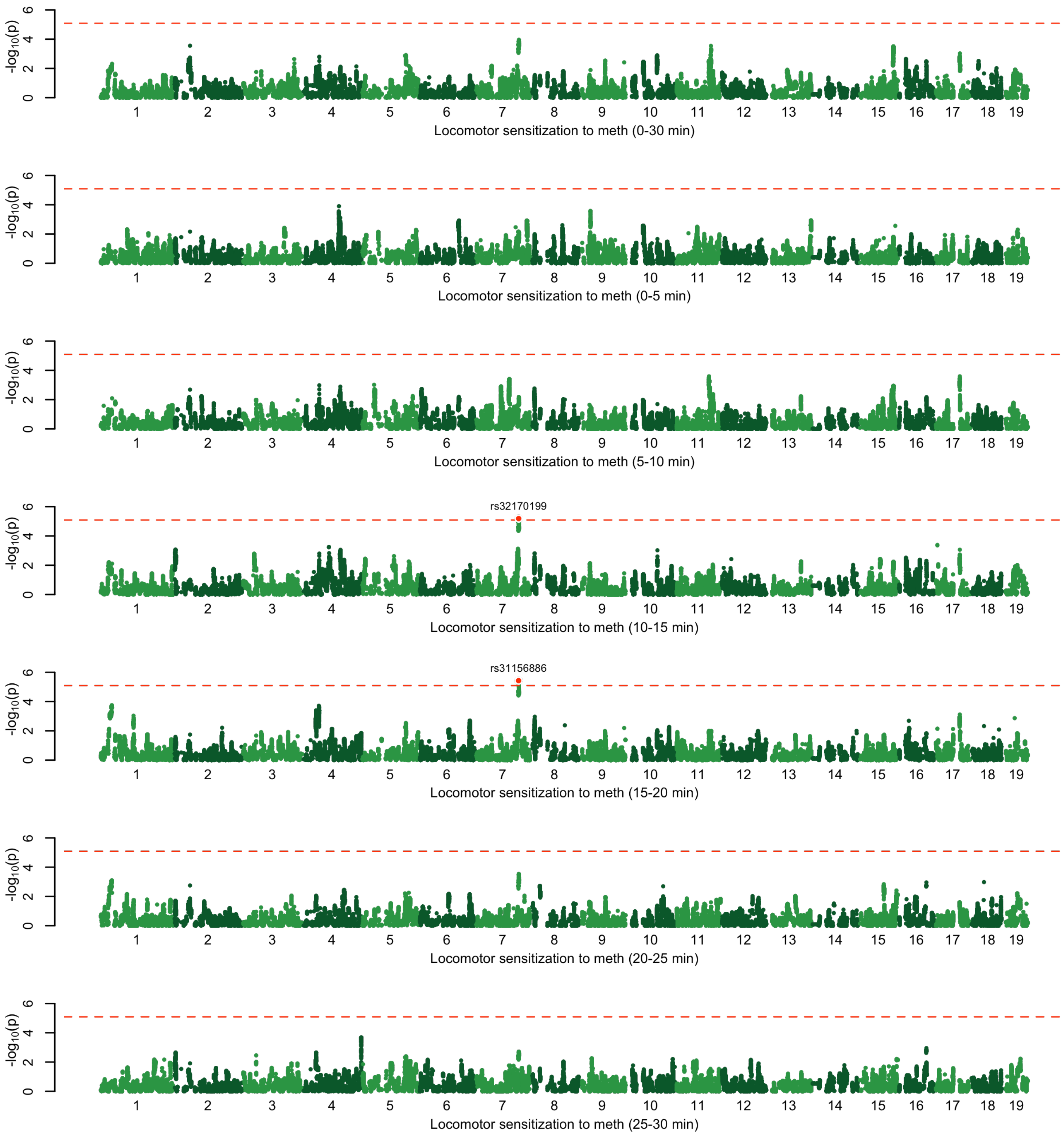

-log10(p)
0
2
4
6
1
2
3
4
5
6
7
8
9
10
11
12
13
14
15
16
17
18
19
Locomotor sensitization to meth (0-30 min)
Locomotor sensitization to meth (0-5 min)
Locomotor sensitization to meth (5-10 min)
rs32170199
Locomotor sensitization to meth (10-15 min)
rs31156886
Locomotor sensitization to meth (15-20 min)
Locomotor sensitization to meth (20-25 min)
Locomotor sensitization to meth (25-30 min)

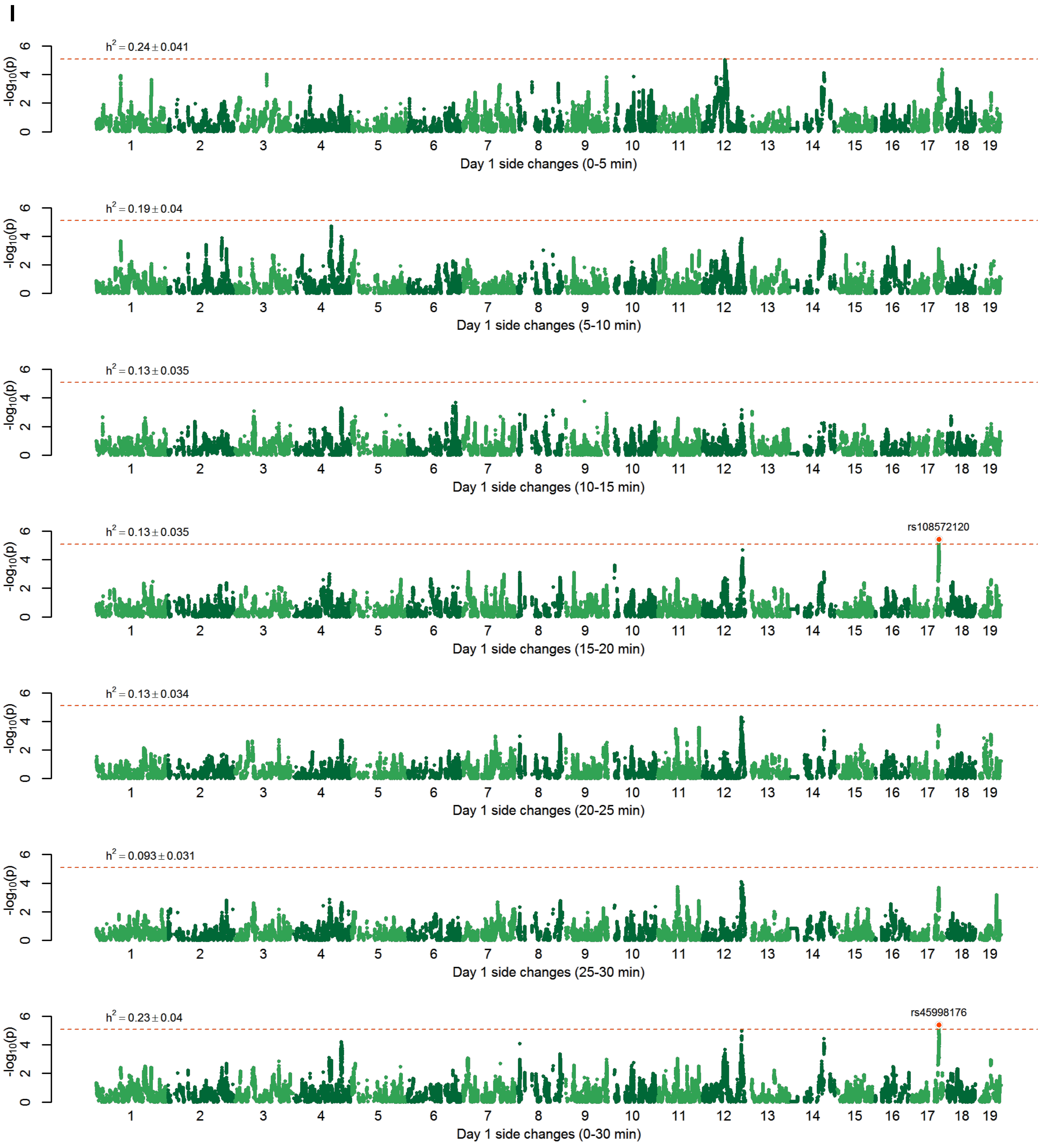

I
h^2 = 0.24 ± 0.041
Day 1 side changes (0-5 min)
h^2 = 0.19 ± 0.04
Day 1 side changes (5-10 min)
h^2 = 0.13 ± 0.035
Day 1 side changes (10-15 min)
h^2 = 0.13 ± 0.035
rs108572120
Day 1 side changes (15-20 min)
h^2 = 0.13 ± 0.034
Day 1 side changes (20-25 min)
h^2 = 0.093 ± 0.031
Day 1 side changes (25-30 min)
h^2 = 0.23 ± 0.04
rs45998176
Day 1 side changes (0-30 min)
-log10(p)

m

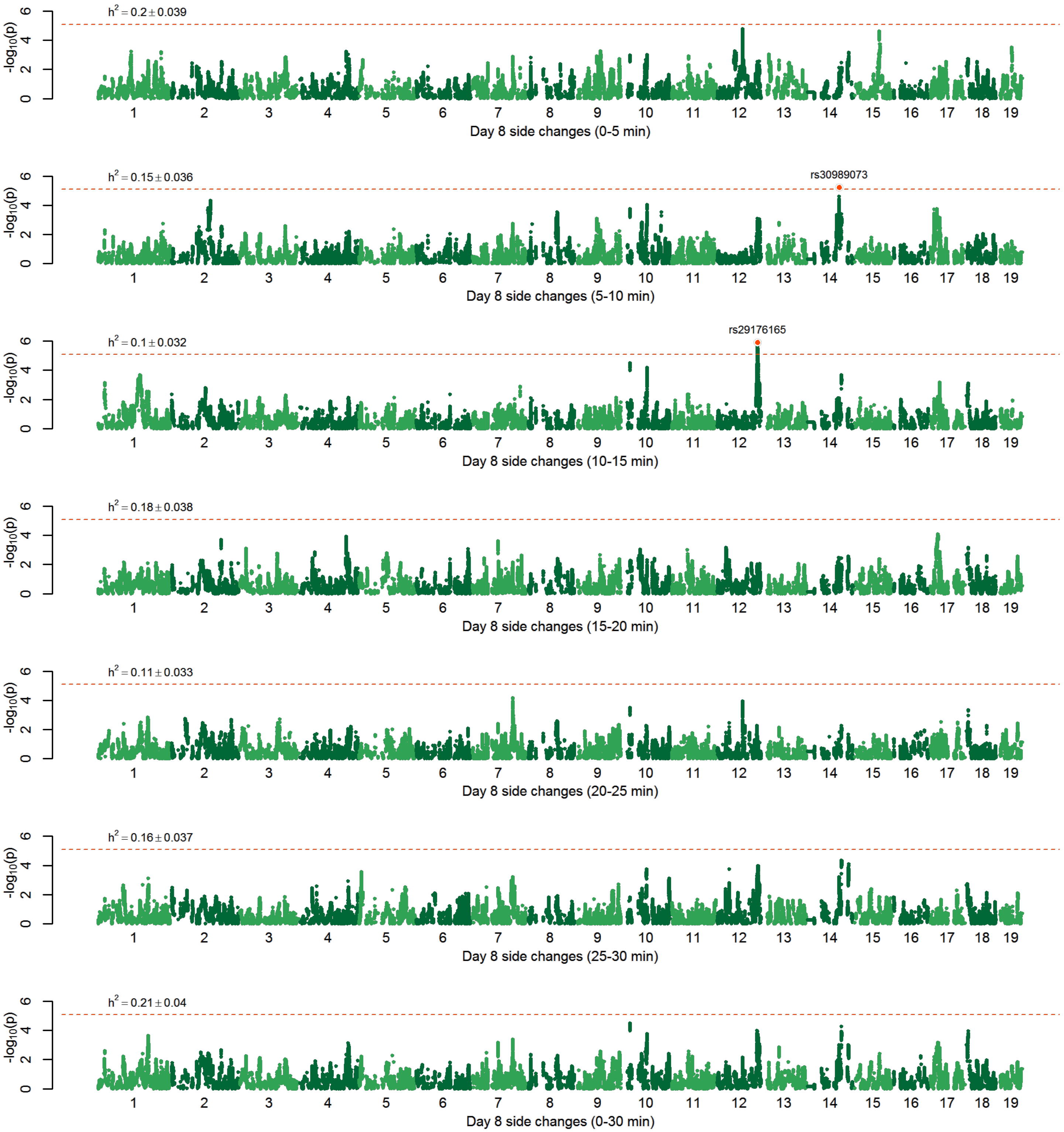
$h^2 = 0.2 \pm 0.039$
-log10(p)
0 2 4 6
1 2 3 4 5 6 7 8 9 10 11 12 13 14 15 16 17 18 19
Day 8 side changes (0-5 min)
$h^2 = 0.15 \pm 0.036$
rs30989073
Day 8 side changes (5-10 min)
$h^2 = 0.1 \pm 0.032$
rs29176165
Day 8 side changes (10-15 min)
$h^2 = 0.18 \pm 0.038$
Day 8 side changes (15-20 min)
$h^2 = 0.11 \pm 0.033$
Day 8 side changes (20-25 min)
$h^2 = 0.16 \pm 0.037$
Day 8 side changes (25-30 min)
$h^2 = 0.21 \pm 0.04$
Day 8 side changes (0-30 min)

n

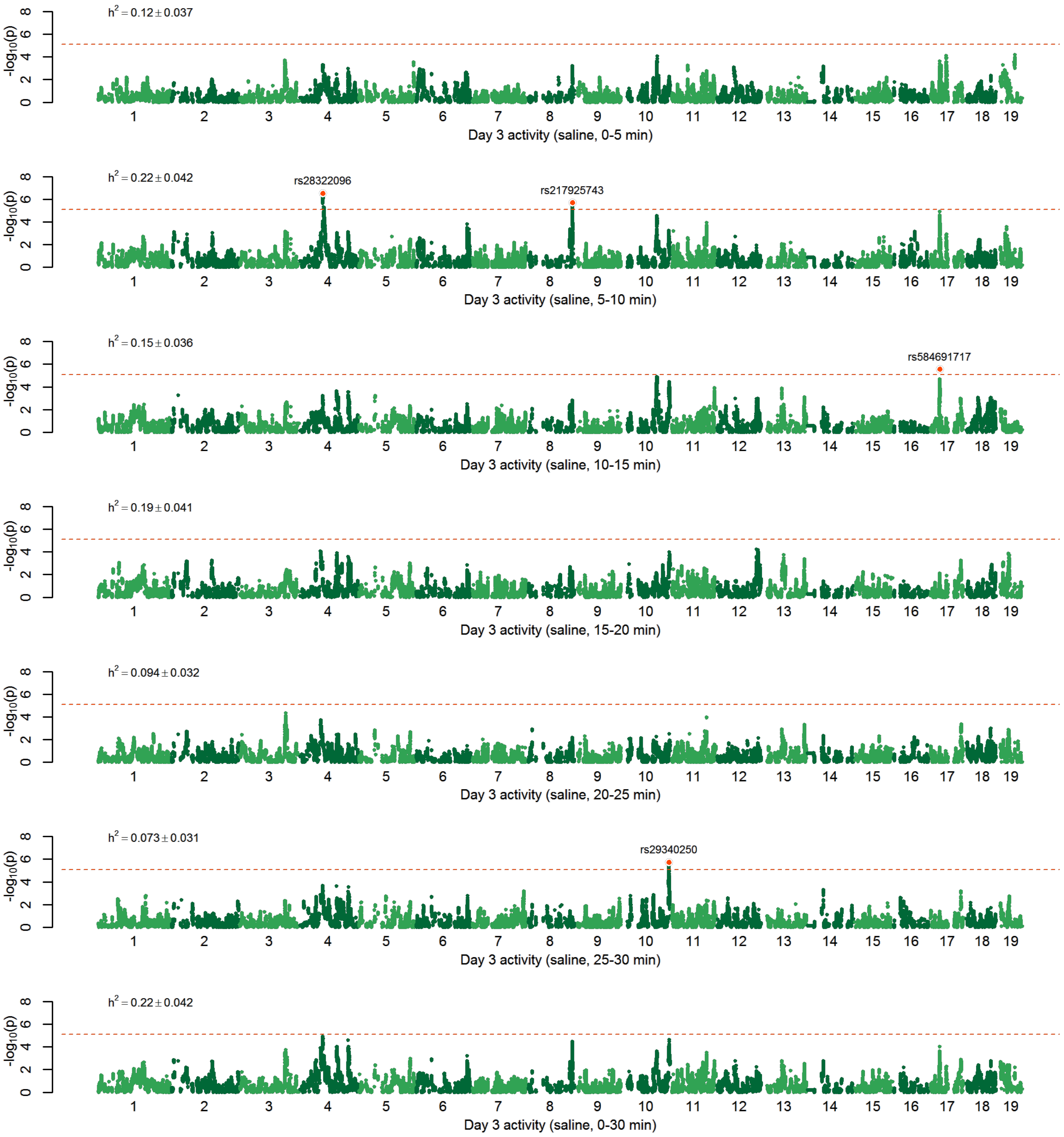

h² = 0.12 ± 0.037
Day 3 activity (saline, 0-5 min)
h² = 0.22 ± 0.042
rs28322096
rs217925743
Day 3 activity (saline, 5-10 min)
h² = 0.15 ± 0.036
rs584691717
Day 3 activity (saline, 10-15 min)
h² = 0.19 ± 0.041
Day 3 activity (saline, 15-20 min)
h² = 0.094 ± 0.032
Day 3 activity (saline, 20-25 min)
h² = 0.073 ± 0.031
rs29340250
Day 3 activity (saline, 25-30 min)
h² = 0.22 ± 0.042
Day 3 activity (saline, 0-30 min)
-log10(p)
1 2 3 4 5 6 7 8 9 10 11 12 13 14 15 16 17 18 19

o

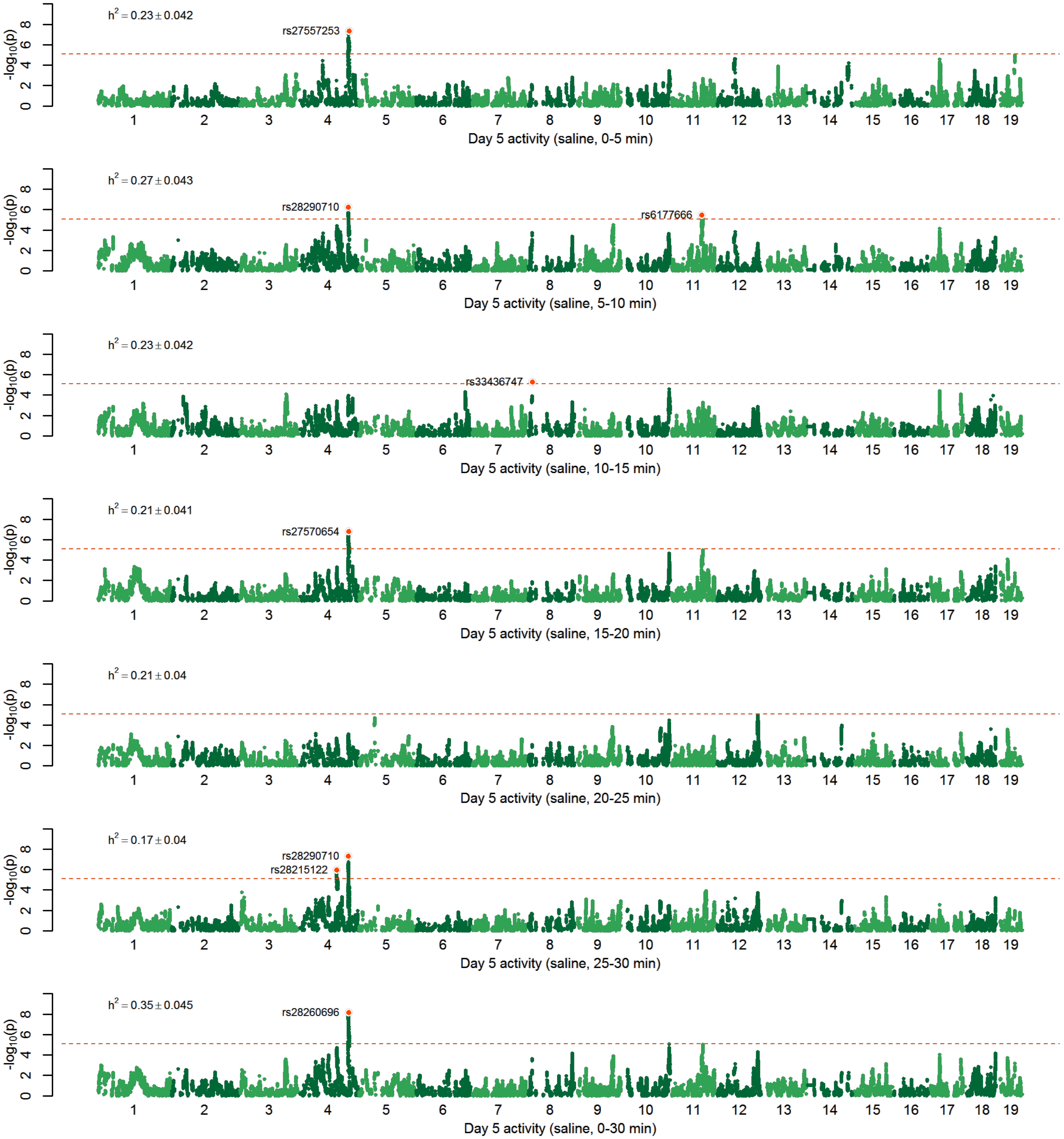
h² = 0.23 ± 0.042
rs27557253
Day 5 activity (saline, 0-5 min)
h² = 0.27 ± 0.043
rs28290710
rs6177666
Day 5 activity (saline, 5-10 min)
h² = 0.23 ± 0.042
rs33436747
Day 5 activity (saline, 10-15 min)
h² = 0.21 ± 0.041
rs27570654
Day 5 activity (saline, 15-20 min)
h² = 0.21 ± 0.04
Day 5 activity (saline, 20-25 min)
h² = 0.17 ± 0.04
rs28290710
rs28215122
Day 5 activity (saline, 25-30 min)
h² = 0.35 ± 0.045
rs28260696
Day 5 activity (saline, 0-30 min)
-log10(p)
1 2 3 4 5 6 7 8 9 10 11 12 13 14 15 16 17 18 19

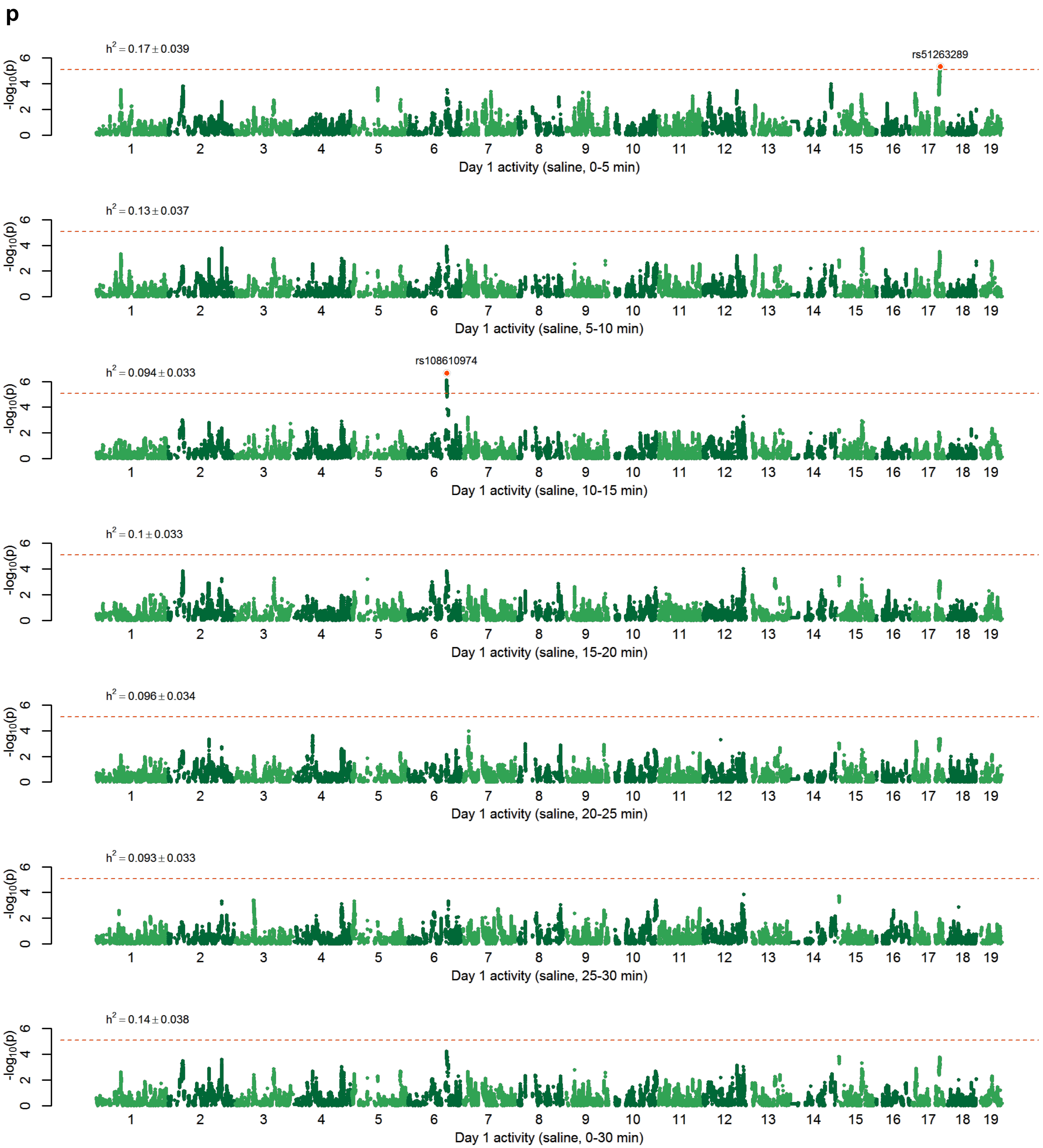
p
h^2 = 0.17 ± 0.039
rs51263289
-log10(p)
Day 1 activity (saline, 0-5 min)
h^2 = 0.13 ± 0.037
Day 1 activity (saline, 5-10 min)
h^2 = 0.094 ± 0.033
rs108610974
Day 1 activity (saline, 10-15 min)
h^2 = 0.1 ± 0.033
Day 1 activity (saline, 15-20 min)
h^2 = 0.096 ± 0.034
Day 1 activity (saline, 20-25 min)
h^2 = 0.093 ± 0.033
Day 1 activity (saline, 25-30 min)
h^2 = 0.14 ± 0.038
Day 1 activity (saline, 0-30 min)

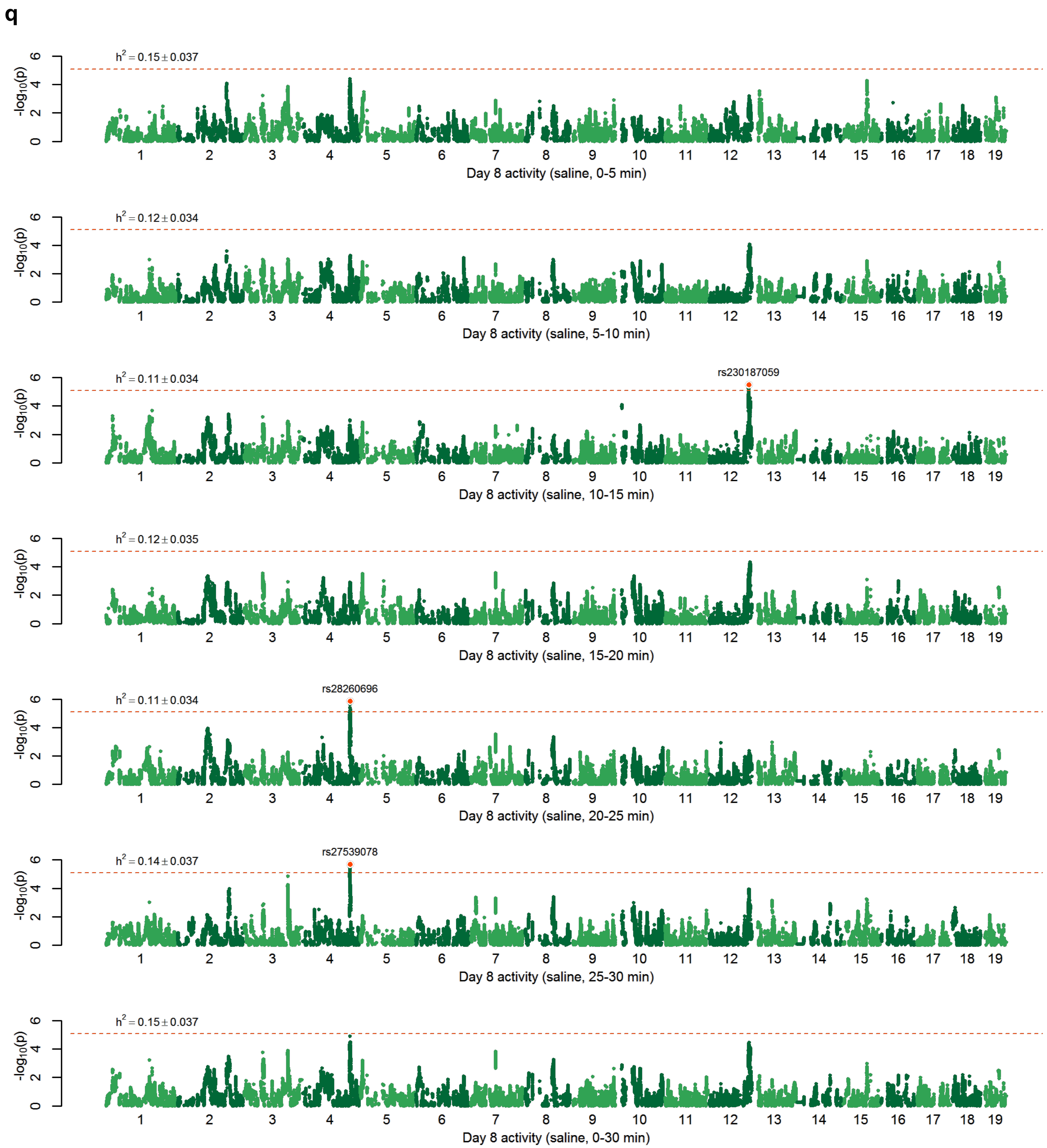
q
$h^2 = 0.15 \pm 0.037$
-log10(p)
Day 8 activity (saline, 0-5 min)
$h^2 = 0.12 \pm 0.034$
Day 8 activity (saline, 5-10 min)
$h^2 = 0.11 \pm 0.034$
rs230187059
Day 8 activity (saline, 10-15 min)
$h^2 = 0.12 \pm 0.035$
Day 8 activity (saline, 15-20 min)
$h^2 = 0.11 \pm 0.034$
rs28260696
Day 8 activity (saline, 20-25 min)
$h^2 = 0.14 \pm 0.037$
rs27539078
Day 8 activity (saline, 25-30 min)
$h^2 = 0.15 \pm 0.037$
Day 8 activity (saline, 0-30 min)

r

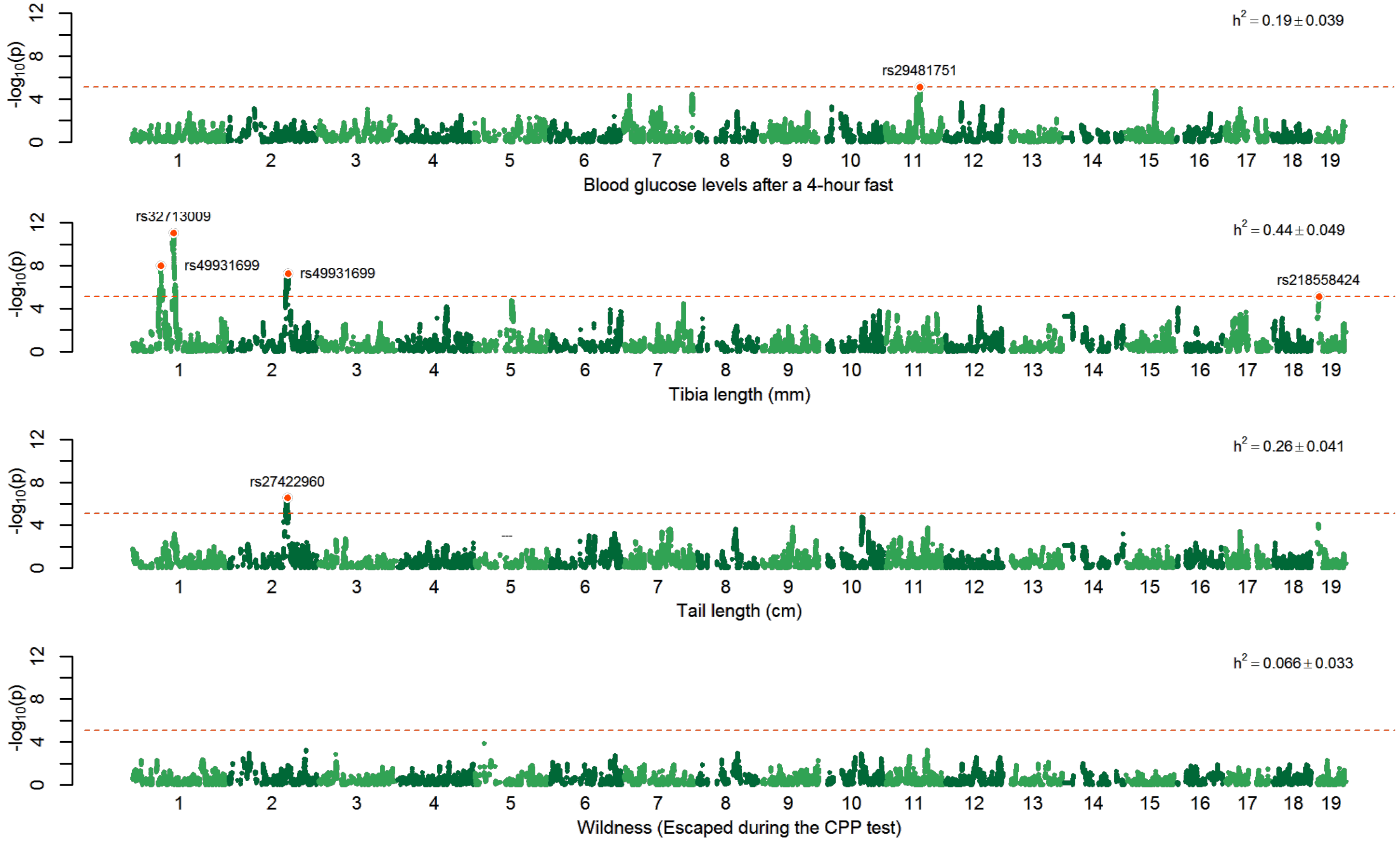
-log10(p)
h2 = 0.19 ± 0.039
rs29481751
Blood glucose levels after a 4-hour fast
h2 = 0.44 ± 0.049
rs32713009
rs49931699
rs49931699
rs218558424
Tibia length (mm)
h2 = 0.26 ± 0.041
rs27422960
Tail length (cm)
h2 = 0.066 ± 0.033
Wildness (Escaped during the CPP test)

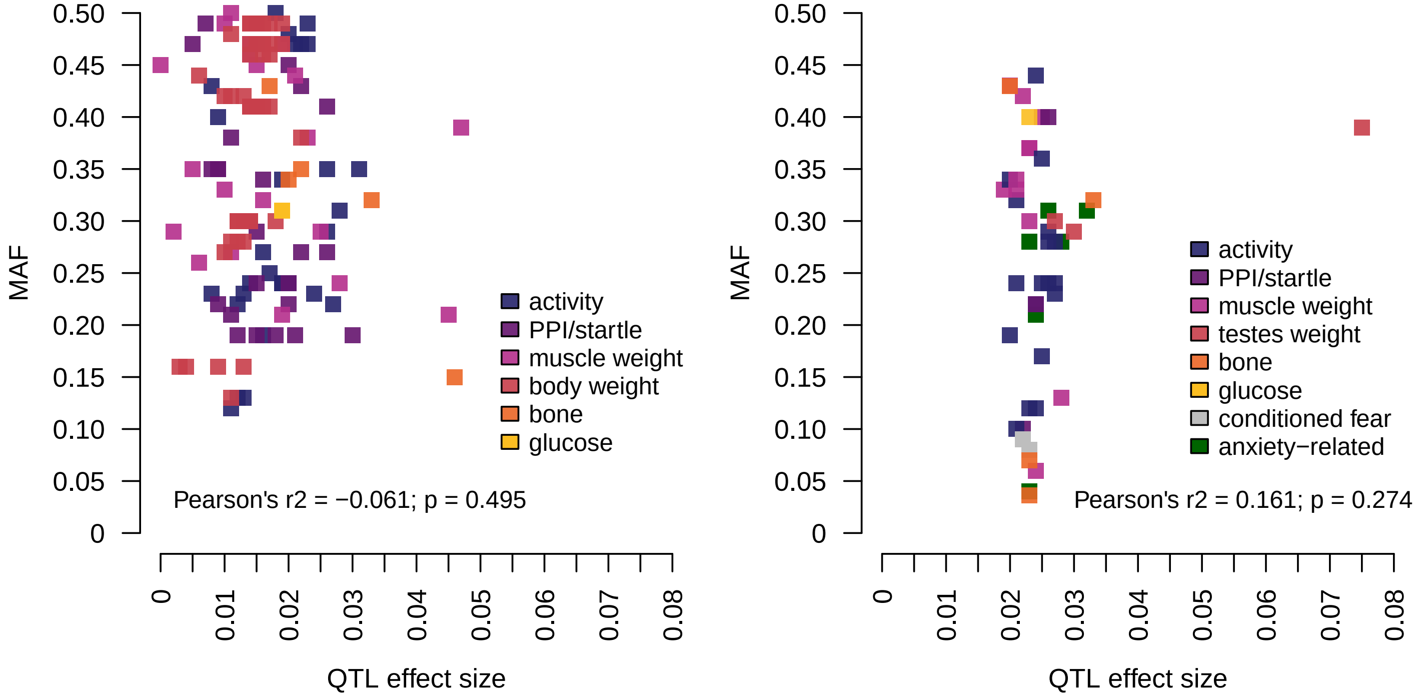

Figure 3: **MAF and locus effect size in G50-56 of the LG $\times$ SM AIL as compared to CFW mice. (a)** The proportion of phenotypic variance explained (PVE) by the most significant SNP at each locus (QTL effect size) is plotted against its MAF for LG $\times$ SM AIL mice. Only genome-wide significant QTLs are shown $(\alpha = 0.05)$ **(b)** QTL effect size vs. MAF in CFW mice. This plot was made using data from Supplementary Table 2 in Parker *et al.* (2016), which lists summary statistics for genome-wide significant QTLs identified at $\alpha = 0.1$.

Figure 4: **Trait correlation heat maps.** Heat maps of Pearson's r^2 correlations for the quantile-normalized residuals of traits measured in G50-56 AIL mice (covariate effects have been removed). We used the cor.test function in R to calculate r^2 using all pairwise complete observations. Significant correlations ($p<0.05$) are marked with an asterisk. We obtained p-values using the cor.test function in R and plotted the data with the R package corrplot. Primary physiological and behavioral traits are summarized in **a-b**; correlations for binned traits are plotted in **c-f**. Sample sizes and descriptions for each trait are provided in **Supplementary Data 2**. **(a)** Physiological traits. AVG weight is the average of weight measured on CPP D1, CPP D8, during PPI, glucose testing, and time of death (i.e. measurements taken one week apart). CPP, PPI, GLU weights are weights for days when CPP, PPI, or glucose levels were tested. RIP (rest in peace) weight is weight at the time of death. TA is tibialis anterior weight, EDL is extensor digitorum longus weight. **(b)** Primary behavioral traits. Only CPP, sensitization and activity traits measured from 0-30 minutes are included (see d-f for binned measurements). 'CPP diff' is the difference in CPP on D8 minus CPP on D1, 'Meth sens is locomotor sensitization to 1 mg/kg methamphetamine (D4 minus D2 activity). Activity (act) trait labels include testing day and bin number separated by a period, and t stands for total activity (0-30 minutes). Saline is locomotor activity following vehicle administration (control) and meth is locomotor activity following methamphetamine administration (1 mg/kg). PPI traits are averaged over two prepulse blocks (3, 6, and 12 refer to the prepulse intensity). AVG startle is the average of startle blocks 1-4. Habituation is the difference between startle block 4 and startle block 1. **(c)** Startle, PPI and habituation. Individual PPI and startle blocks are shown; trait labels are the same as in **(b)**. PPI3, PPI6, and PPI12 refer to prepulse intensity (3, 6, or 12 dB above 70 dB background noise). **(d)** CPP. Labels for binned measurements are as described in **(b)**. CPP diff is the difference between D8 minus D1 preference. CPP1 and CPP8 refer to CPP on D1 (initial preference for the left side of the testing chamber before it was paired with methamphetamine) and D8 (preference for the left side of the testing chamber after conditioning). **(e)** Saline-induced activity. Correlations for saline activity (act) for D1, D3, D5, and D8 and side changes on D1 and D8 are shown. Trait labels include testing day and bin number separated by a period. For example, act1.1 stands for D1 activity during bin 1 (0-5 minutes) and t stands for total activity (0-30 minutes). On D1 and D8, mice are allowed to explore both sides of the CPP testing chamber, and there is more total area to explore. On D3 and D5, mice are restricted to the right side of the chamber. **(f)** Methamphetamine-induced activity. Correlations for activity in response to methamphetamine and locomotor sensitization.

a

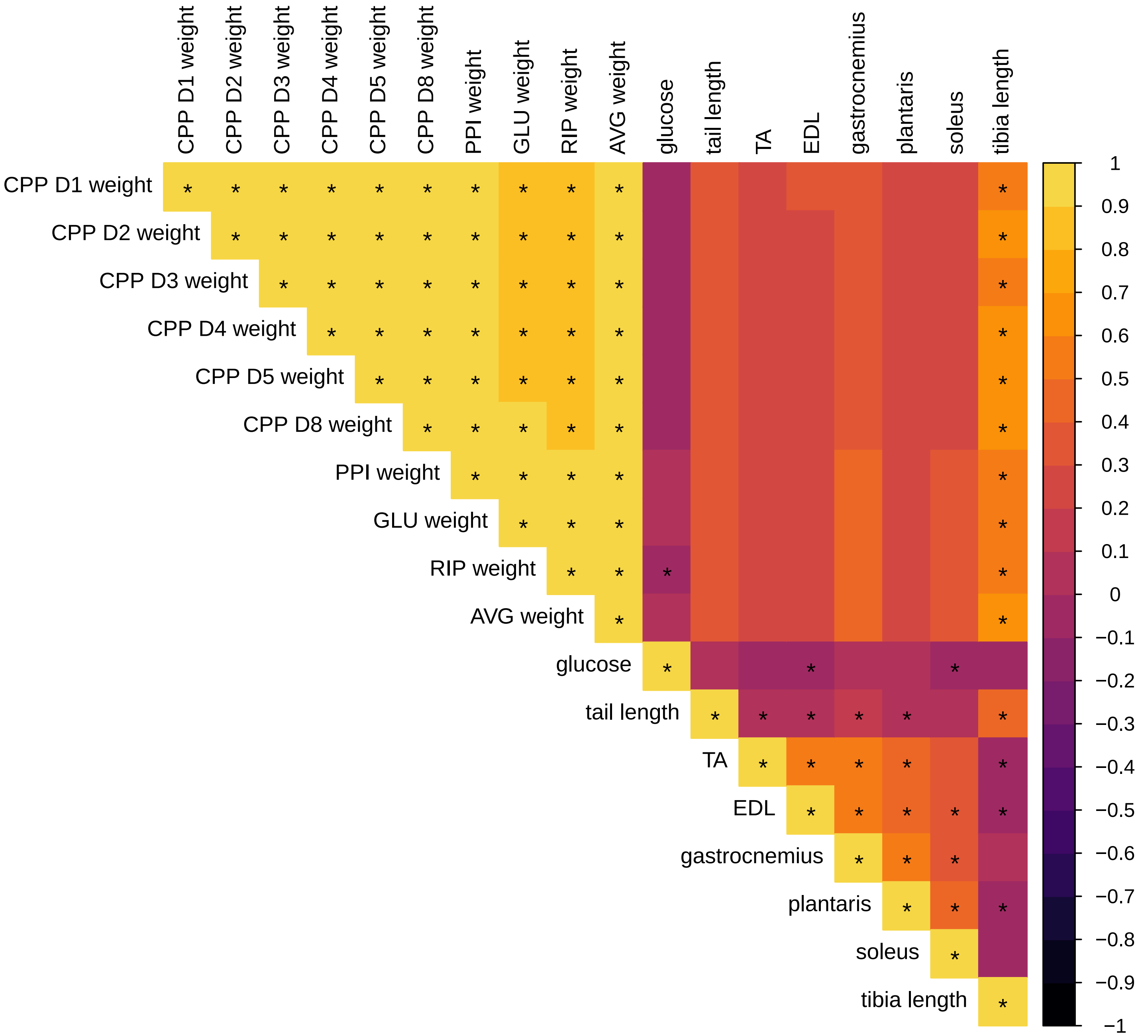
CPP D1 weight
CPP D2 weight
CPP D3 weight
CPP D4 weight
CPP D5 weight
CPP D8 weight
PPI weight
GLU weight
RIP weight
AVG weight
glucose
tail length
TA
EDL
gastrocnemius
plantaris
soleus
tibia length
1
0.9
0.8
0.7
0.6
0.5
0.4
0.3
0.2
0.1
0
−0.1
−0.2
−0.3
−0.4
−0.5
−0.6
−0.7
−0.8
−0.9
−1

b

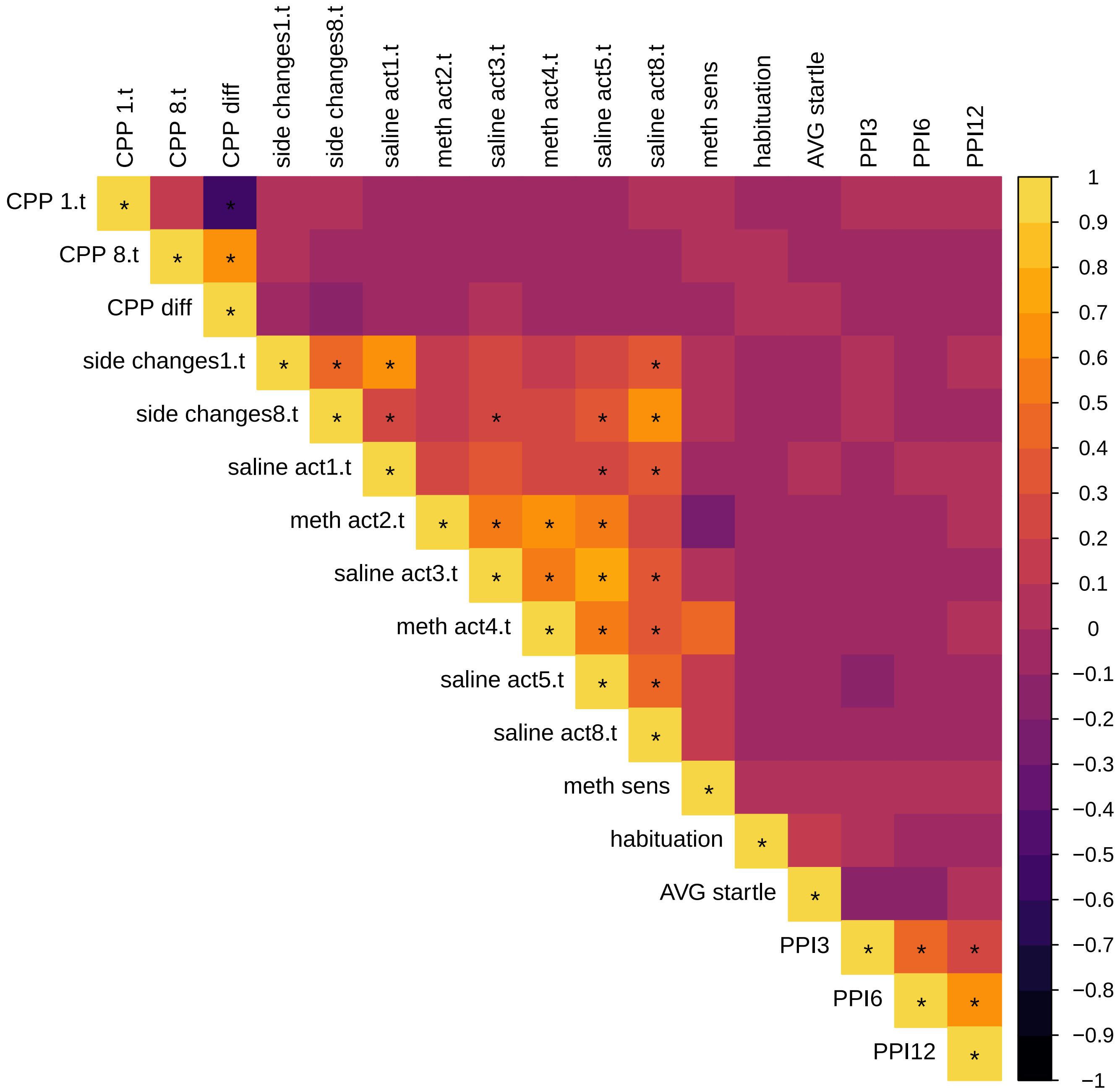
CPP 1.t
CPP 8.t
CPP diff
side changes1.t
side changes8.t
saline act1.t
meth act2.t
saline act3.t
meth act4.t
saline act5.t
saline act8.t
meth sens
habituation
AVG startle
PPI3
PPI6
PPI12
1
0.9
0.8
0.7
0.6
0.5
0.4
0.3
0.2
0.1
0
−0.1
−0.2
−0.3
−0.4
−0.5
−0.6
−0.7
−0.8
−0.9
−1

c

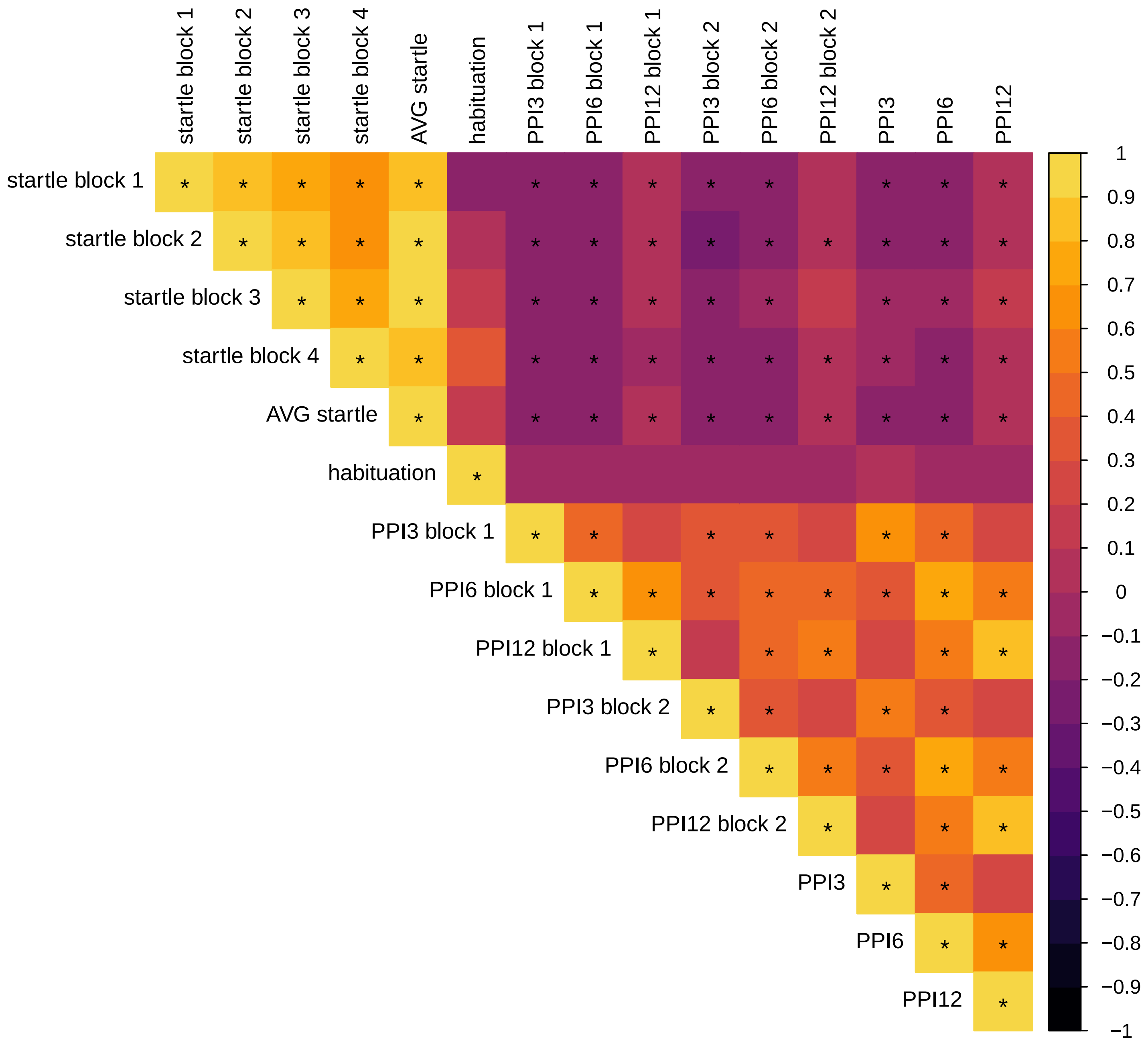
startle block 1
startle block 2
startle block 3
startle block 4
AVG startle
habituation
PPI3 block 1
PPI6 block 1
PPI12 block 1
PPI3 block 2
PPI6 block 2
PPI12 block 2
PPI3
PPI6
PPI12
1
0.9
0.8
0.7
0.6
0.5
0.4
0.3
0.2
0.1
0
−0.1
−0.2
−0.3
−0.4
−0.5
−0.6
−0.7
−0.8
−0.9
−1

d

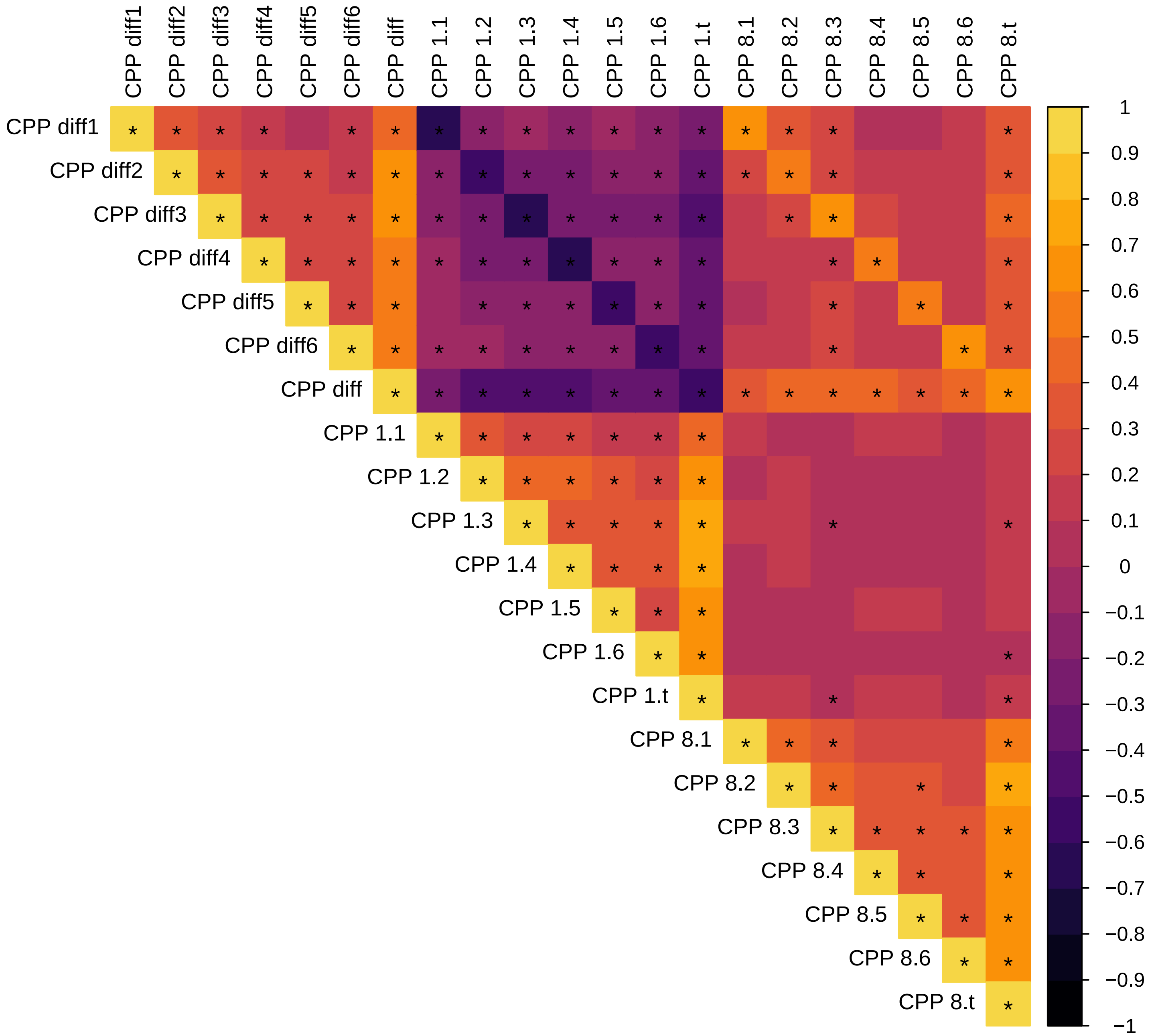
CPP diff1
CPP diff2
CPP diff3
CPP diff4
CPP diff5
CPP diff6
CPP diff
CPP 1.1
CPP 1.2
CPP 1.3
CPP 1.4
CPP 1.5
CPP 1.6
CPP 1.t
CPP 8.1
CPP 8.2
CPP 8.3
CPP 8.4
CPP 8.5
CPP 8.6
CPP 8.t
1
0.9
0.8
0.7
0.6
0.5
0.4
0.3
0.2
0.1
0
−0.1
−0.2
−0.3
−0.4
−0.5
−0.6
−0.7
−0.8
−0.9
−1

e

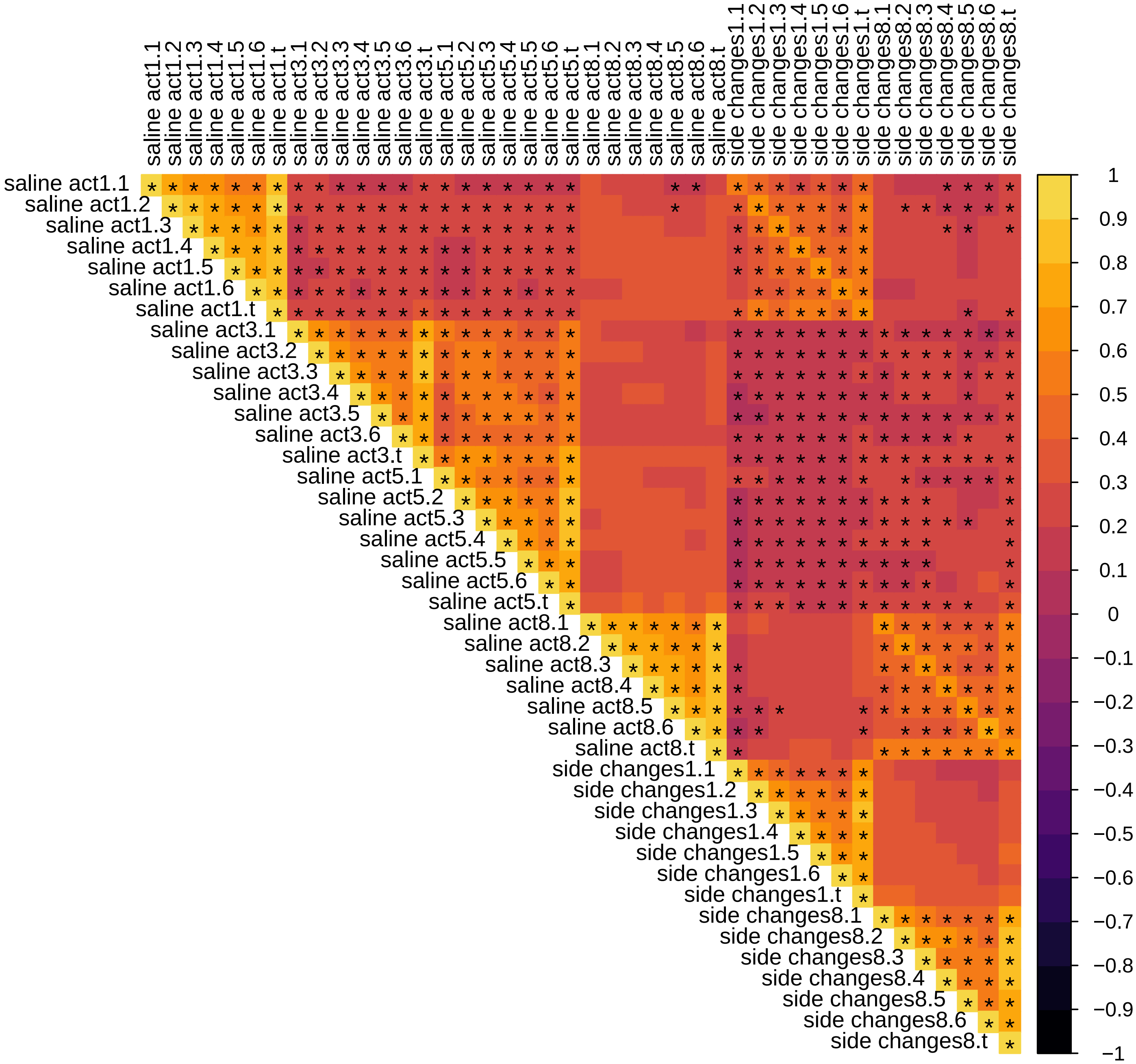
saline act1.1
saline act1.2
saline act1.3
saline act1.4
saline act1.5
saline act1.6
saline act1.t
saline act3.1
saline act3.2
saline act3.3
saline act3.4
saline act3.5
saline act3.6
saline act3.t
saline act5.1
saline act5.2
saline act5.3
saline act5.4
saline act5.5
saline act5.6
saline act5.t
saline act8.1
saline act8.2
saline act8.3
saline act8.4
saline act8.5
saline act8.6
saline act8.t
side changes1.1
side changes1.2
side changes1.3
side changes1.4
side changes1.5
side changes1.6
side changes1.t
side changes8.1
side changes8.2
side changes8.3
side changes8.4
side changes8.5
side changes8.6
side changes8.t
1
0.9
0.8
0.7
0.6
0.5
0.4
0.3
0.2
0.1
0
−0.1
−0.2
−0.3
−0.4
−0.5
−0.6
−0.7
−0.8
−0.9
−1

f

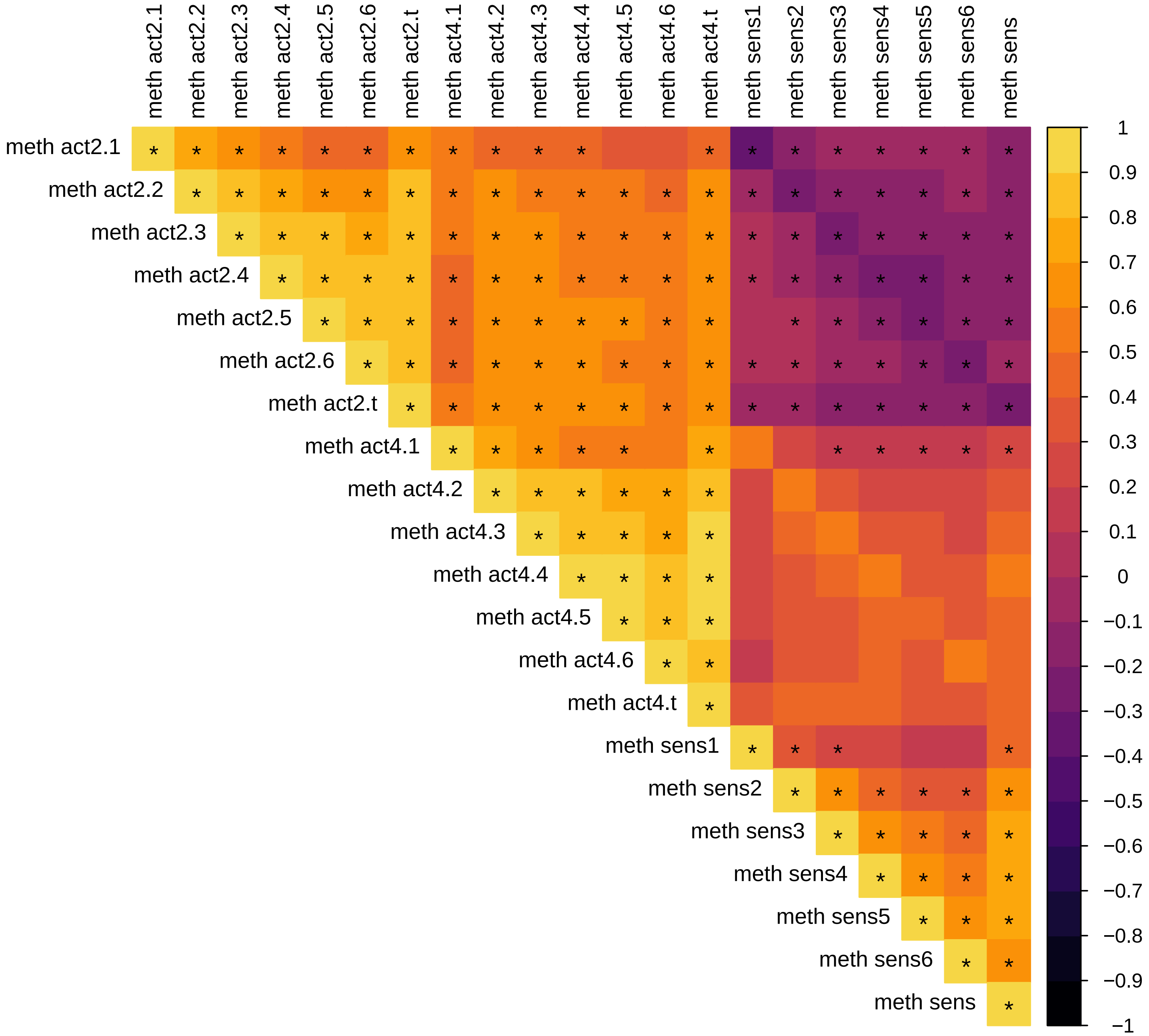
meth act2.1
meth act2.2
meth act2.3
meth act2.4
meth act2.5
meth act2.6
meth act2.t
meth act4.1
meth act4.2
meth act4.3
meth act4.4
meth act4.5
meth act4.6
meth act4.t
meth sens1
meth sens2
meth sens3
meth sens4
meth sens5
meth sens6
meth sens
1
0.9
0.8
0.7
0.6
0.5
0.4
0.3
0.2
0.1
0
−0.1
−0.2
−0.3
−0.4
−0.5
−0.6
−0.7
−0.8
−0.9
−1

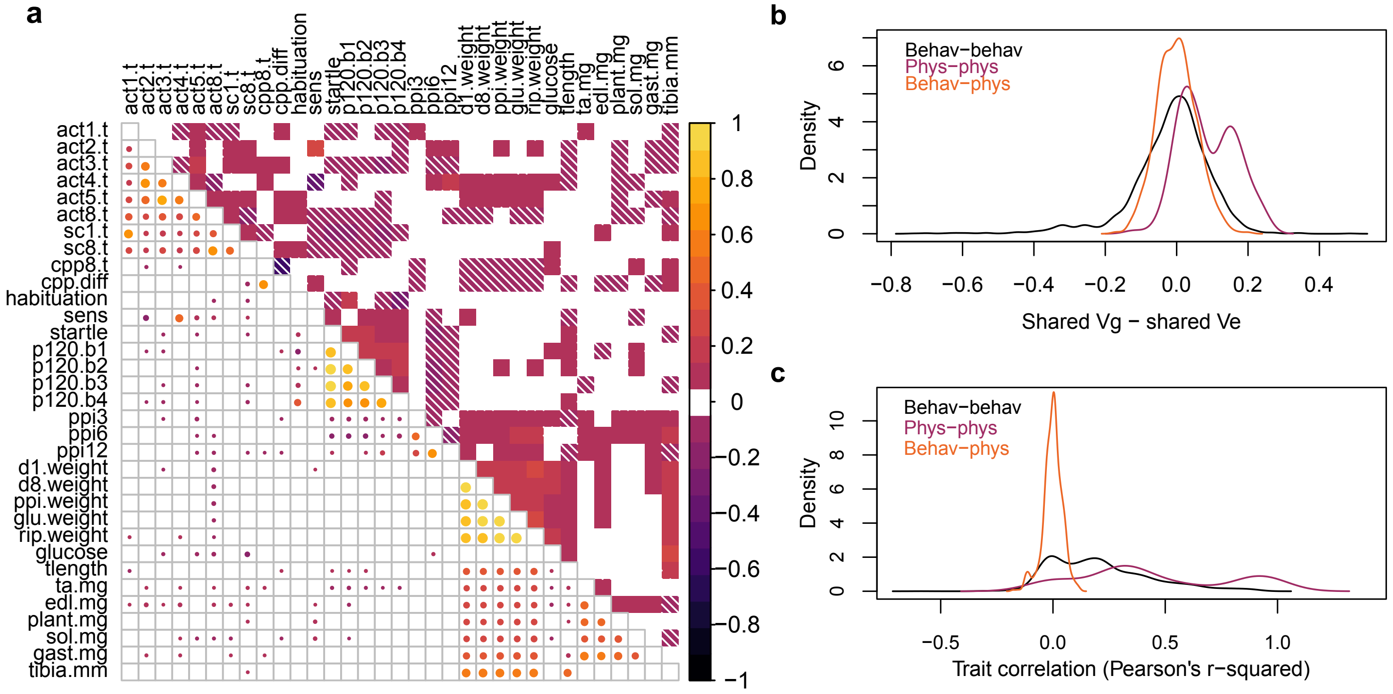

Figure 5: **Genetic and environmental correlations between traits. (a)** Matrix of trait correlations (lower triangle) plotted beside the difference between Vg, the genetic variance, and Ve, the environmental variance shared by each pair of traits (upper triangle). For clarity, only significant trait correlations are shown (Pearson's r^2 , $p<0.05$), and only primary traits are plotted (correlation data is available in **Supplementary Data 3** and **Supplementary Fig. 4**). The sign and magnitude of each correlation is depicted by color and circle size, respectively. In the upper triangle, the difference between Vg and Ve is indicated with color. Negative values, which are marked by white diagonal lines, indicate that the environmental variance shared by the two traits is greater than their shared genetic variance. Shared Vg, Ve and their standard errors are provided in **Supplementary Data 3**. **(b)** Density plot of the difference between shared Vg and shared Ve for all pairs of traits (including sub-traits not pictured in **(a)**). Values are plotted separately for pairs of behavioral traits (black), pairs of physiological traits (purple), and behavioral-physiological trait pairs (orange). Negative values indicate that the environmental variance shared by the two traits is greater than the genetic variance. Positive values indicate that the shared genetic variance is greater. **(c)** Density plot of trait correlations for pairs of behavioral traits, pairs of physiological traits, and behavioral-physiological trait pairs.

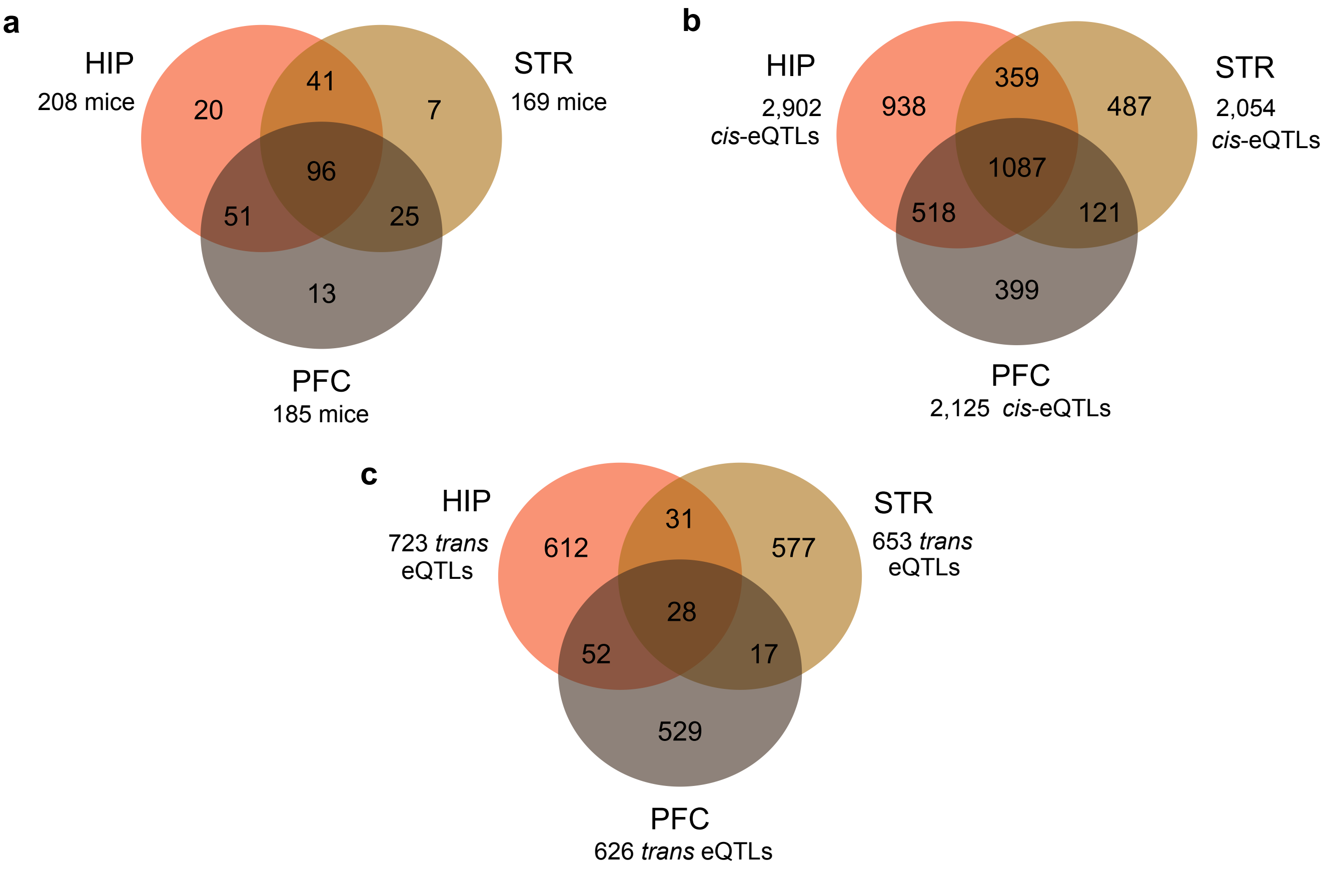

Figure 6: **Summary of eQTLs by brain region. (a)** Number of overlapping samples analyzed from each tissue after quality control. **(b)** Number of *cis*-eQTL genes identified in each tissue at FDR<0.05. **(c)** Number of genes with *trans*-eQTLs identified in HIP $(p = 9.01 \times 10^{-6})$, PFC $(p = 1.04 \times 10^{-5})$, and STR $(p = 8.68 \times 10^{-6})$ at $\alpha = 0.05$.

a

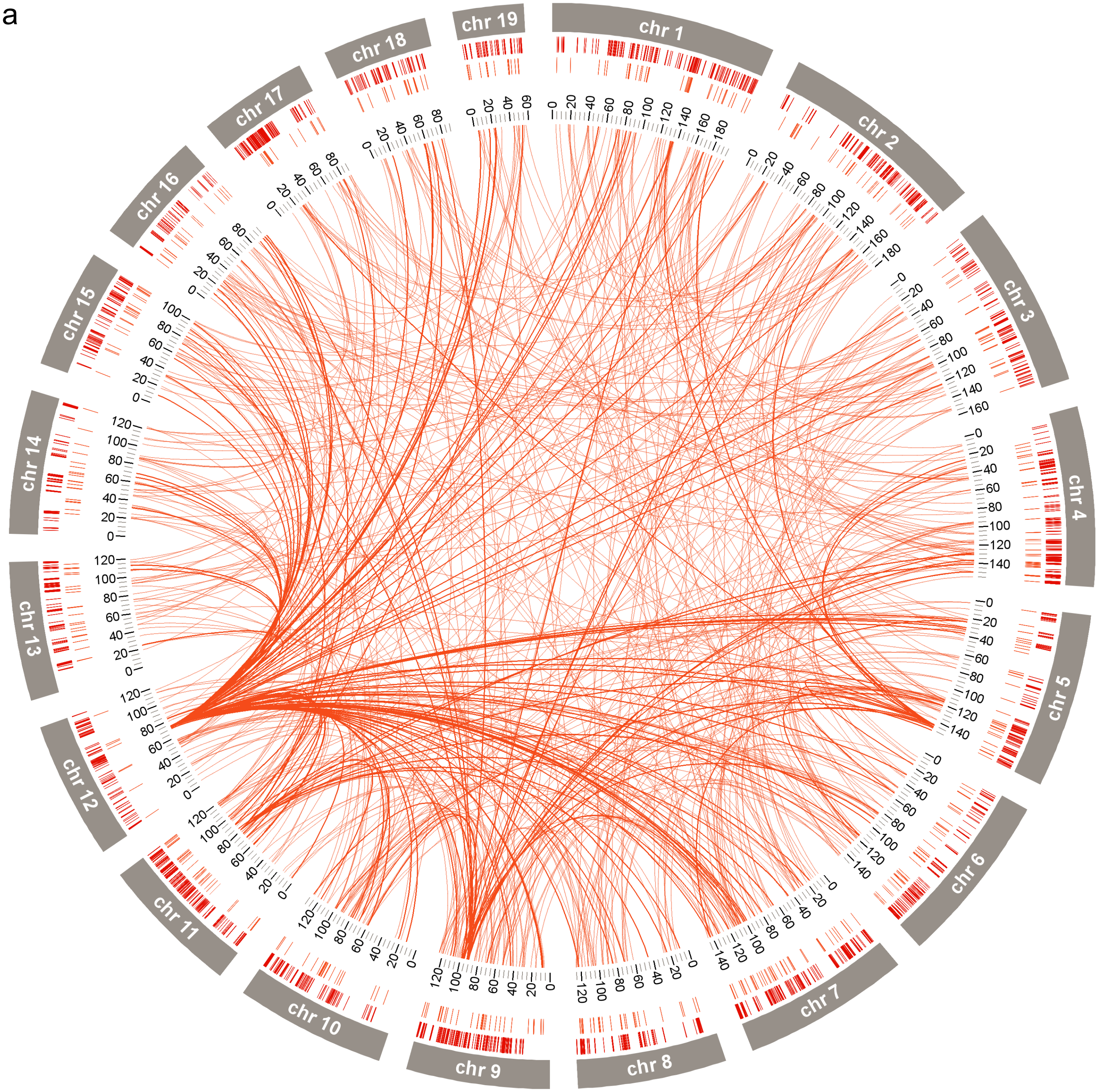

Figure 7: ***cis*-eQTLs and *trans*-eQTLs in HIP, PFC and STR. (a)** Circos plot of eQTLs in HIP. In **a-c**, each ring inside the circle shows locations of *cis*-eQTLs. *trans*-eQTLs are shown inside the circle. Increasing line opacity indicates *trans*-eQTLs that were associated with a greater number of *trans*-eQTL genes.

b

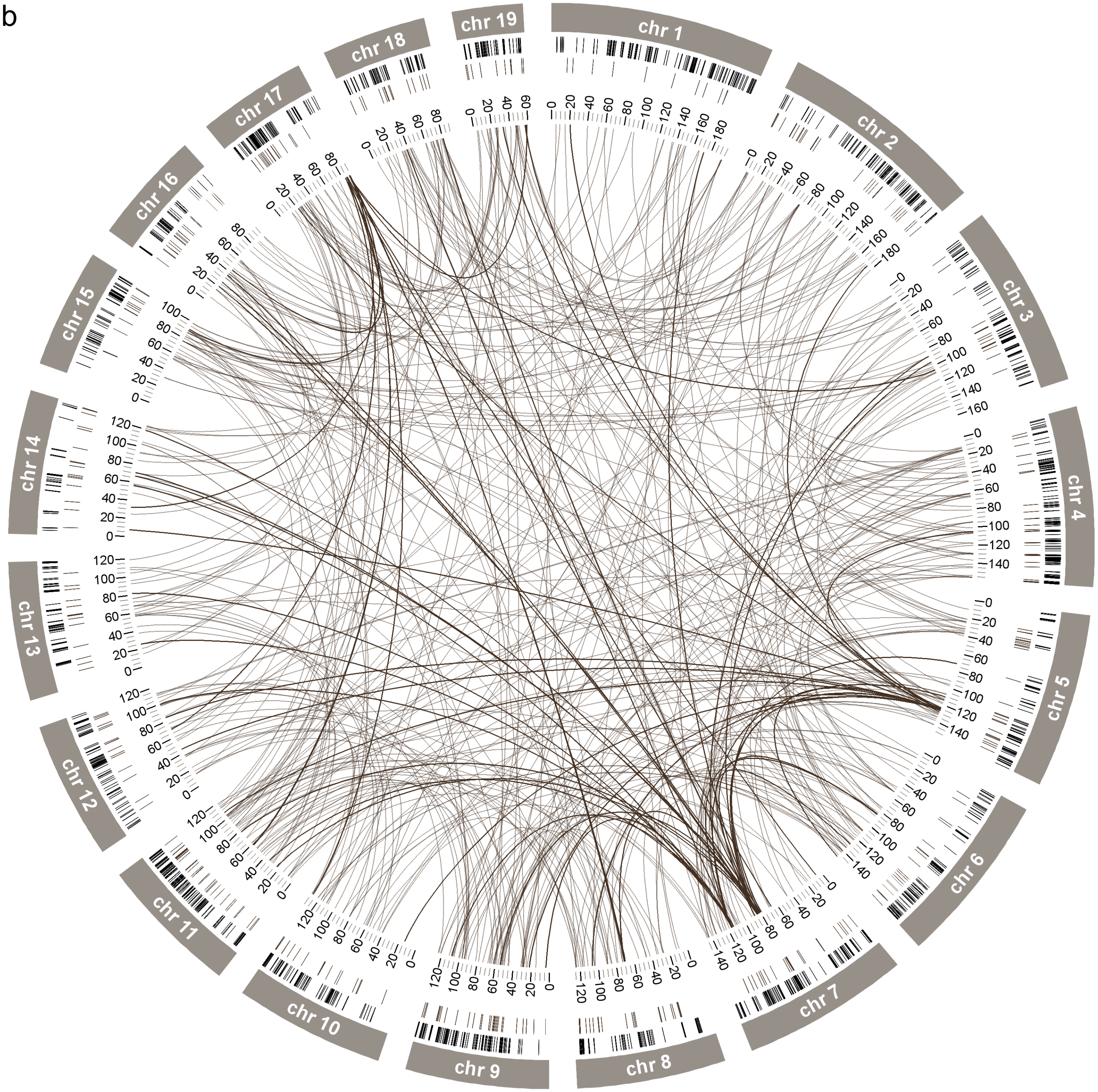

Figure 7: ***cis*-eQTLs and *trans*-eQTLs in HIP, PFC and STR. (b)** Circos plot of eQTLs in PFC. In **a-c**, each ring inside the circle shows locations of *cis*-eQTLs. *trans*-eQTLs are shown inside the circle. Increasing line opacity indicates *trans*-eQTLs that were associated with a greater number of *trans*-eQTL genes.

c

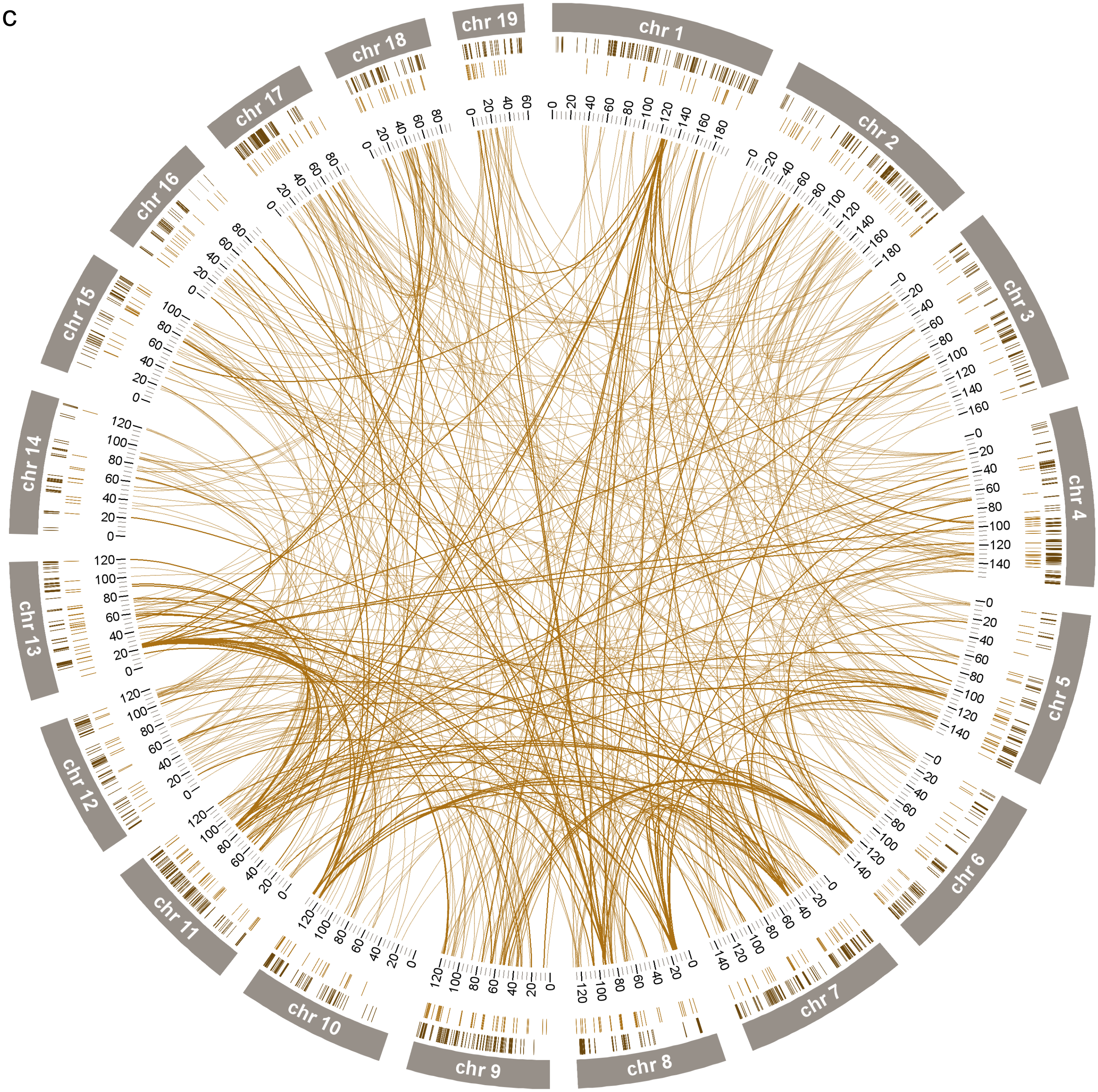

Figure 7: ***cis*-eQTLs and *trans*-eQTLs in HIP, PFC and STR. (c)** Circos plot of eQTLs in STR. In **a-c**, each ring inside the circle shows locations of *cis*-eQTLs. *trans*-eQTLs are shown inside the circle. Increasing line opacity indicates *trans*-eQTLs that were associated with a greater number of *trans*-eQTL genes.

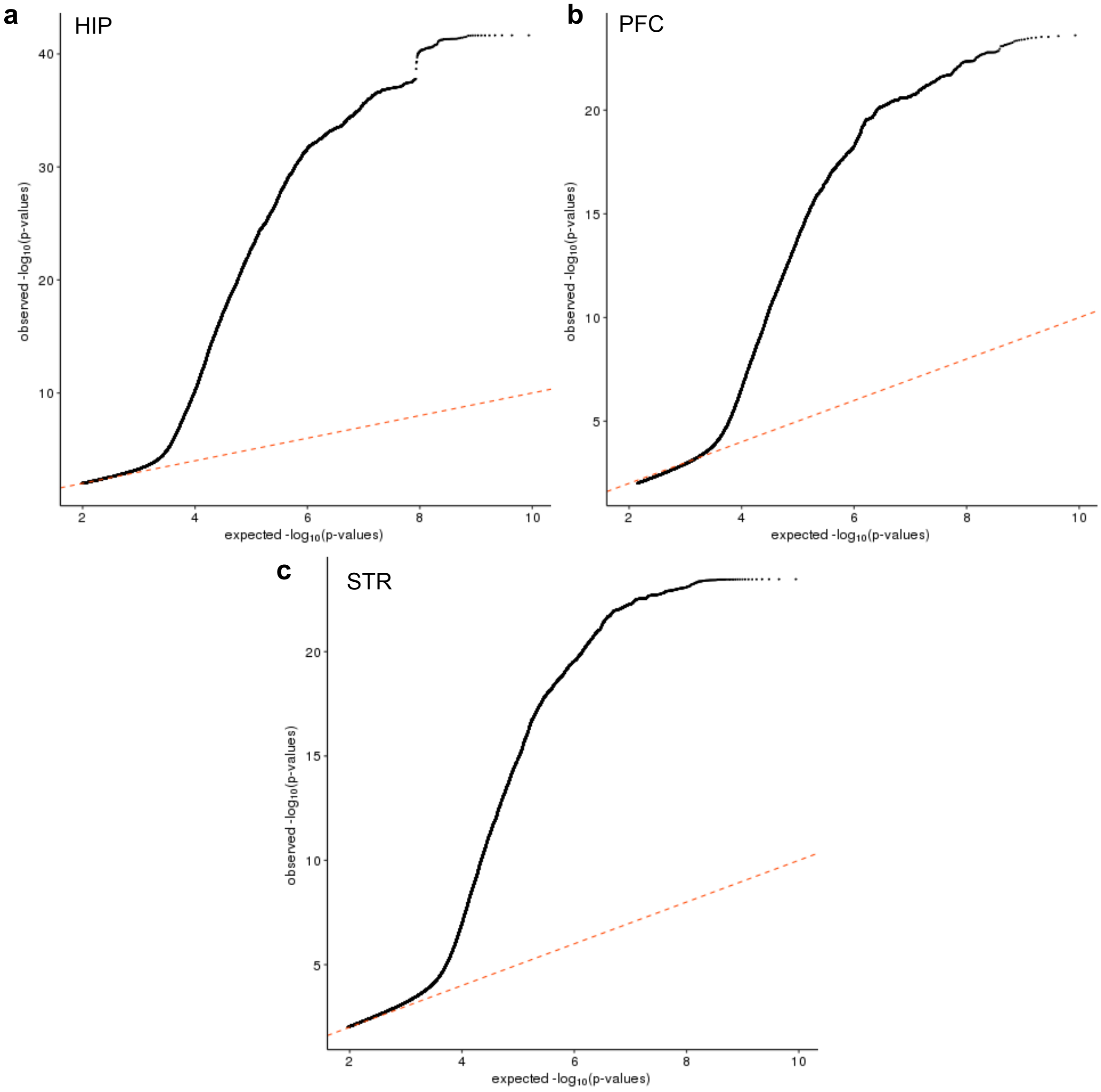

Figure 8: **Quantile-quantile plot of *trans*-eQTLs in HIP, PFC and STR. (a)** Quantile-quantile plots for *trans*-eQTLs in HIP, **(b)** PFC, and **(c)** STR. Each plot compares quantiles of all observed p-values less than 0.01 for all genes tested in each tissue (y-axis) against expected quantiles under the null (uniform) distribution of p-values (x-axis).

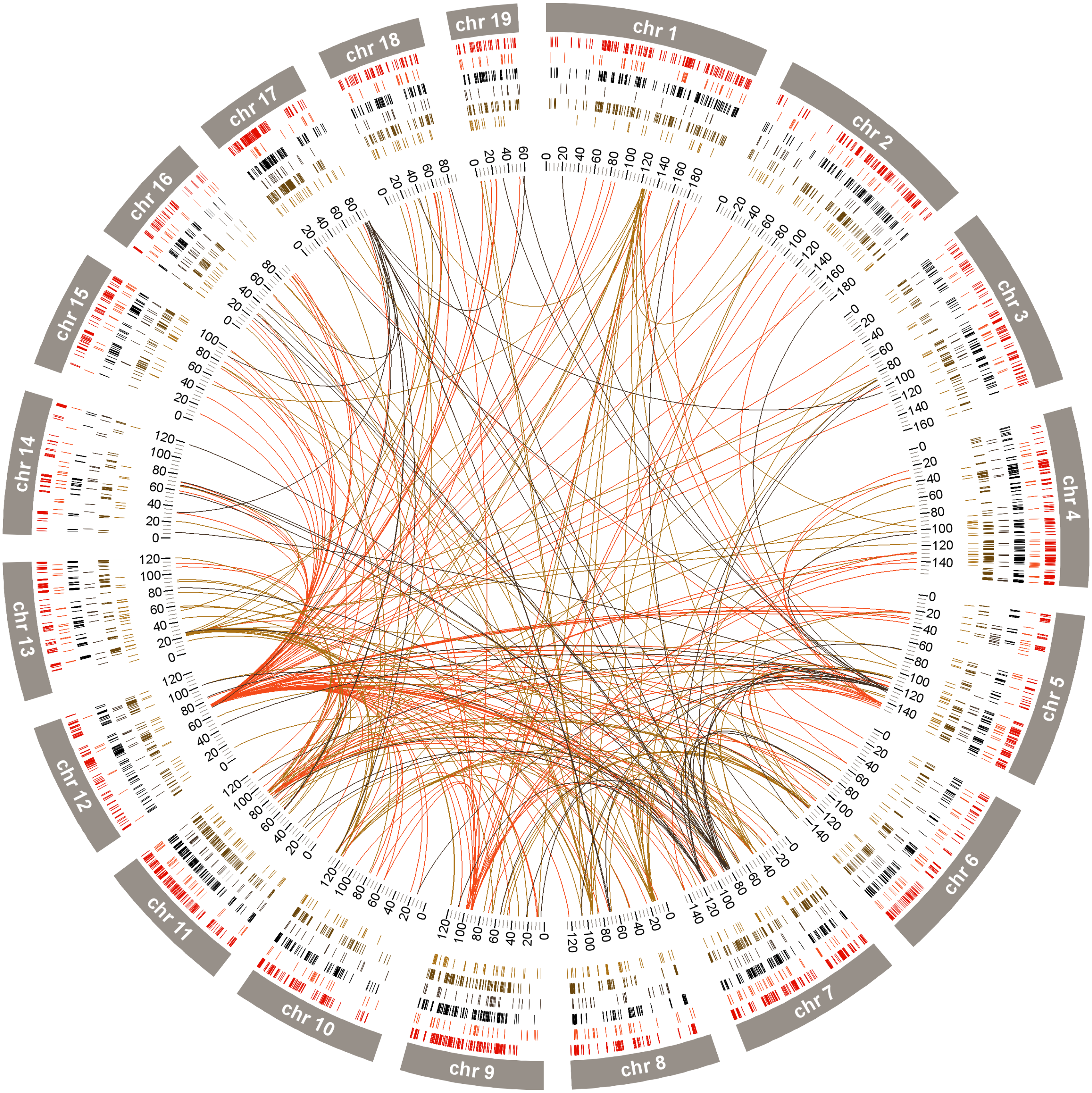

Figure 9: **Master eQTLs in HIP, PFC and STR.** We identified over 7,000 *cis*-eQTLs (FDR<0.05) and 2,002 *trans*-eQTLs ($\alpha = 0.05$) in HIP (n=208; outer red for *cis*-eQTLs, inner red for *trans*-eQTLs), PFC (n=185; outer black for *cis*-eQTLs, inner black for *trans*-eQTL) and STR (n=169; outer brown for *cis*-eQTLs, inner brown for *trans*-eQTLs). We also identified master eQTLs, which we defined as loci that regulate the expression of ten or more target genes in a given tissue (central lines link master eQTLs to eQTL genes).

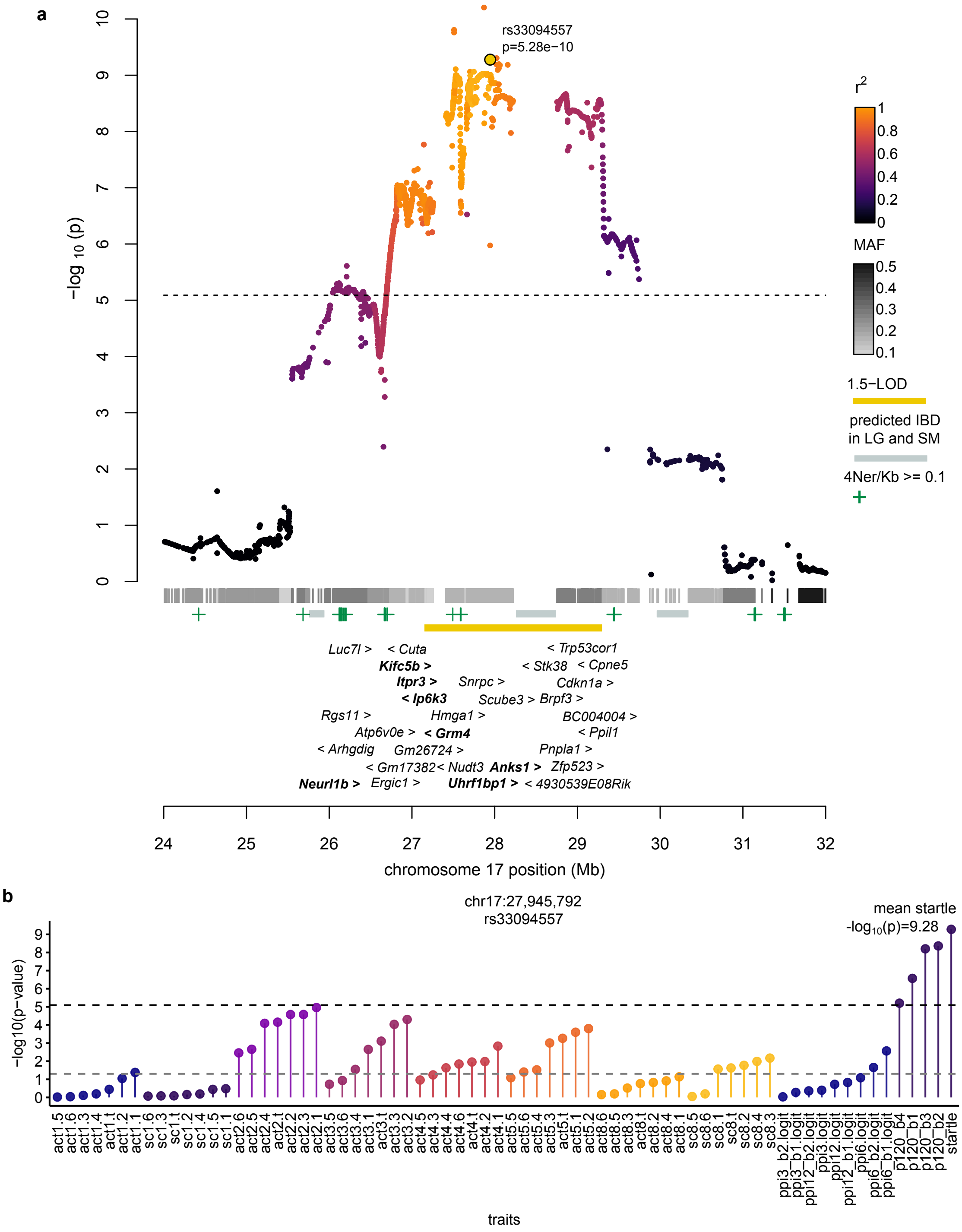

Figure 10: A locus strongly associated with startle on chromosome 17 has pleiotropic effects on behavior. (a) Regional association plot of the association between rs33094557 (gold dot) and the mean startle response. The location of *cis*-eQTL genes, 1.5-LOD interval (gold bar), areas of elevated recombination from Brunschwig *et al.* (plus symbols), regions predicted by Nikolskiy *et al.* to be nearly IBD between LG and SM (grey bars), and SNP MAFs (grey heatmap) are indicated. Points are colored by LD (r^2) with rs33094557. The dashed line indicates a significance threshold of $-log_{10}(p) = 5.09(\alpha = 0.05)$. eQTL genes containing missense mutations (dbSNP v.142) are highlighted with bold text. There are two SNPs which appear more significant than rs33094557; however, they did not exhibit strong LD with nearby SNPs. We considered that these SNPs might be imputation errors. Therefore, we conservatively listed rs33094557, the most significant SNP that was consistent with adjacent markers, as the top association. We used rs33094557 to calculate the 1.5-LOD interval. **(b)** PheWAS plot showing an association on chromosome 17 between rs33094557 and other traits measured in this study. Dashed lines mark the genome-wide significance threshold as in **(a)** and a nominal significance level of p=0.05. Traits are listed by ID, grouped by type and sorted in ascending order of association with rs33094557 (full trait descriptions are provided in Supplementary Data 2).

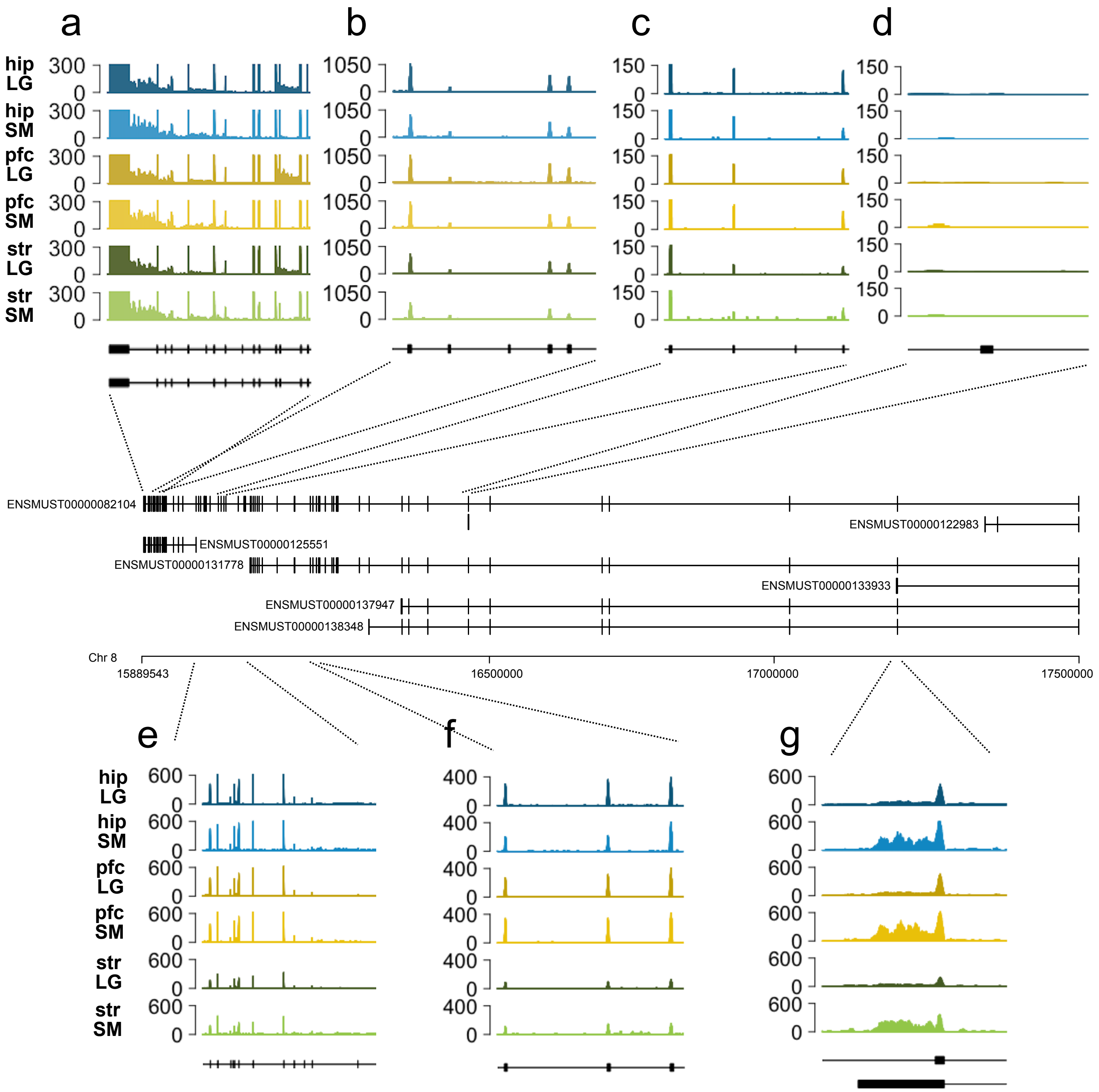

Figure 11: **Average *Csmd1* read coverage for SM and LG homozygotes in HIP, PFC and STR.** *Csmd1* coverage in each tissue is averaged by genotype class. Only mice that were consistently homozygous-LG or consistently homozygous-SM throughout the *Csmd1* locus were included in the average (we excluded mice that had more than two inconsistent genotypes among the 559 SNPs in the region). HIP had 13 SM and 93 LG homozygotes, PFC had 11 SM and 101 LG homozygotes, and STR had 10 SM and 79 LG homozygotes. Reads are plotted as RPKM (length normalization was performed with respect to a bin size of 0.001 kb). Ensembl (release 90) annotates seven transcript structures for *Csmd1*, among which only ENSMUST00000082104 is known to code for a protein. Read coverage indicates the (differential) expression of alternative, presumably non-coding transcripts. Transcript ENSMUST00000125551 is believed to retain intronic sequence, supporting intronic reads differing consistently between LG and SM in all three tissues **(a)**. Conversely, SM samples seem to express alternative transcript ENSMUST00000133933 at higher levels than LG across tissues **(g)**. Overall, LG and SM samples exhibit a similar expression pattern of exons unique to the protein coding transcript **(e)**. Several exons, however, show a slightly higher read coverage in LG than in SM, e.g. first and last exon in **(b)**, and first and second exon (from left) in **(f)**, HIP. Furthermore, samples seem to express novel transcripts missing in the annotation (missing read coverage in *b*-*d*).

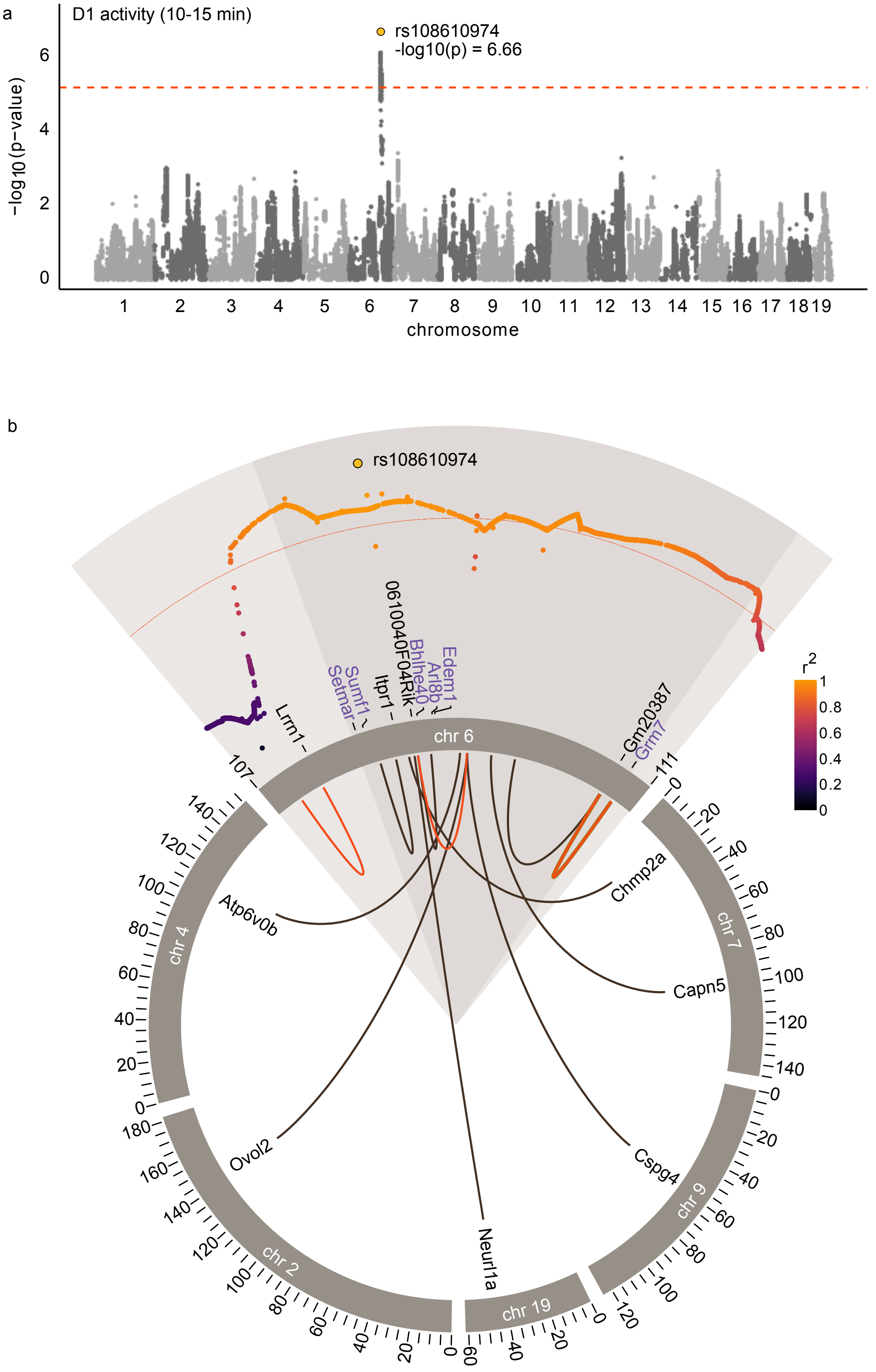

Figure 12: **Several *trans*-eQTLs located in *Itpr1* overlap a locus associated with D1 side changes on chromosome 6. (a)** Manhattan plot of loci identified for D1 side changes (10-15 min). The most significant SNP on chromosome 6 (rs108610974; $p = 2.18 \times 10^{-7}$) is highlighted in gold in both plots. The dashed lines in each plot indicate a permutation-derived threshold of $p = 8.06 \times 10^{-6}$, or $-log_{10}(p) = 5.09 (\alpha = 0.05)$. **(b)** Circos plot highlighting eQTLs that overlap the region associated with D1 side changes. A zoomed-in plot of the locus is shown on chromosome 6 with $-log_{10}$ (p-values) on the y-axis and physical position (Mb) on the x-axis. The dark shaded region highlights the 1.5-LOD interval, and SNPs are colored according to LD (r^2) with rs108610974. eQTL genes are shown in black and other genes at the locus that did not have eQTLs are shown in purple. The inner circle shows eQTLs and their target genes; HIP eQTLs are shown in orange, PFC eQTLs are shown in black, and one STR eQTL is shown in brown. Only chromosomes targeted by *trans*-eQTLs at the chromosome 6 region are plotted.

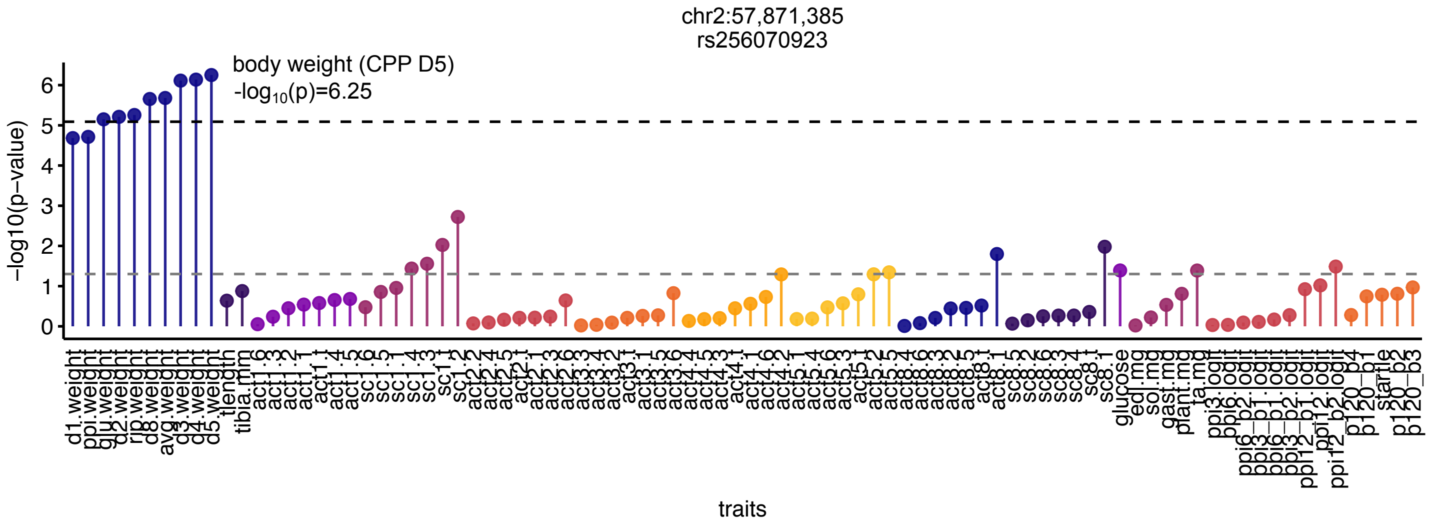

Figure 13: **Pleiotropic effects of a locus associated with body weight on chromosome 2.** PheWAS plot of an association on chromosome 2 between rs256070923 and other traits measured in this study. Dashed lines mark the genome-wide significance threshold $-log_{10}(p) = 5.09(\alpha = 0.05)$ and a nominal significance level of $p = 0.05$. Traits are listed by ID, grouped by type and sorted in ascending order of association with rs256070923 (full trait descriptions are provided in **Supplementary Data 2**).

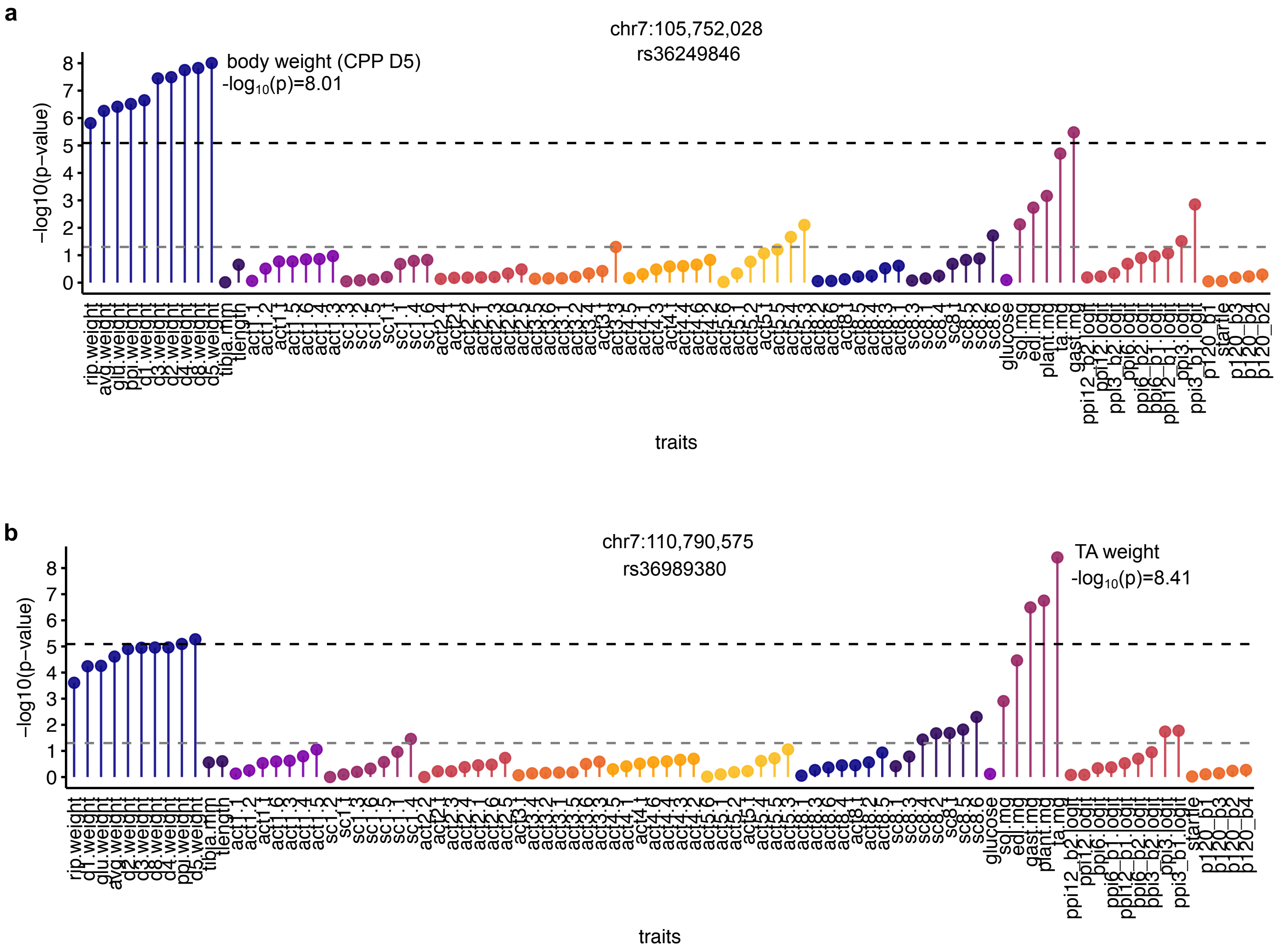

Figure 14: **A locus on chromosome 7 has pleiotropic effects on body weight and TA mass. (a)** PheWAS plot of an association on chromosome 7 between rs36249846 (the top SNP for body weight on D5 of the CPP test at this locus) and other traits measured in this study. **(b)** PheWAS plot of an association between rs36989380 (the top SNP for TA mass at this locus) and other traits measured in this study. rs36249846 and rs36989380 are located 5.04 Mb apart; LD (r^2) between the two SNPs is 0.557. Dashed lines mark the genome-wide significance threshold $-log_{10}(p) = 5.09(\alpha = 0.05)$ and a nominal significance level of $p = 0.05$. Traits are listed by ID, grouped by type and sorted in ascending order of association with the top SNP (full trait descriptions are provided in **Supplementary Data 2**).

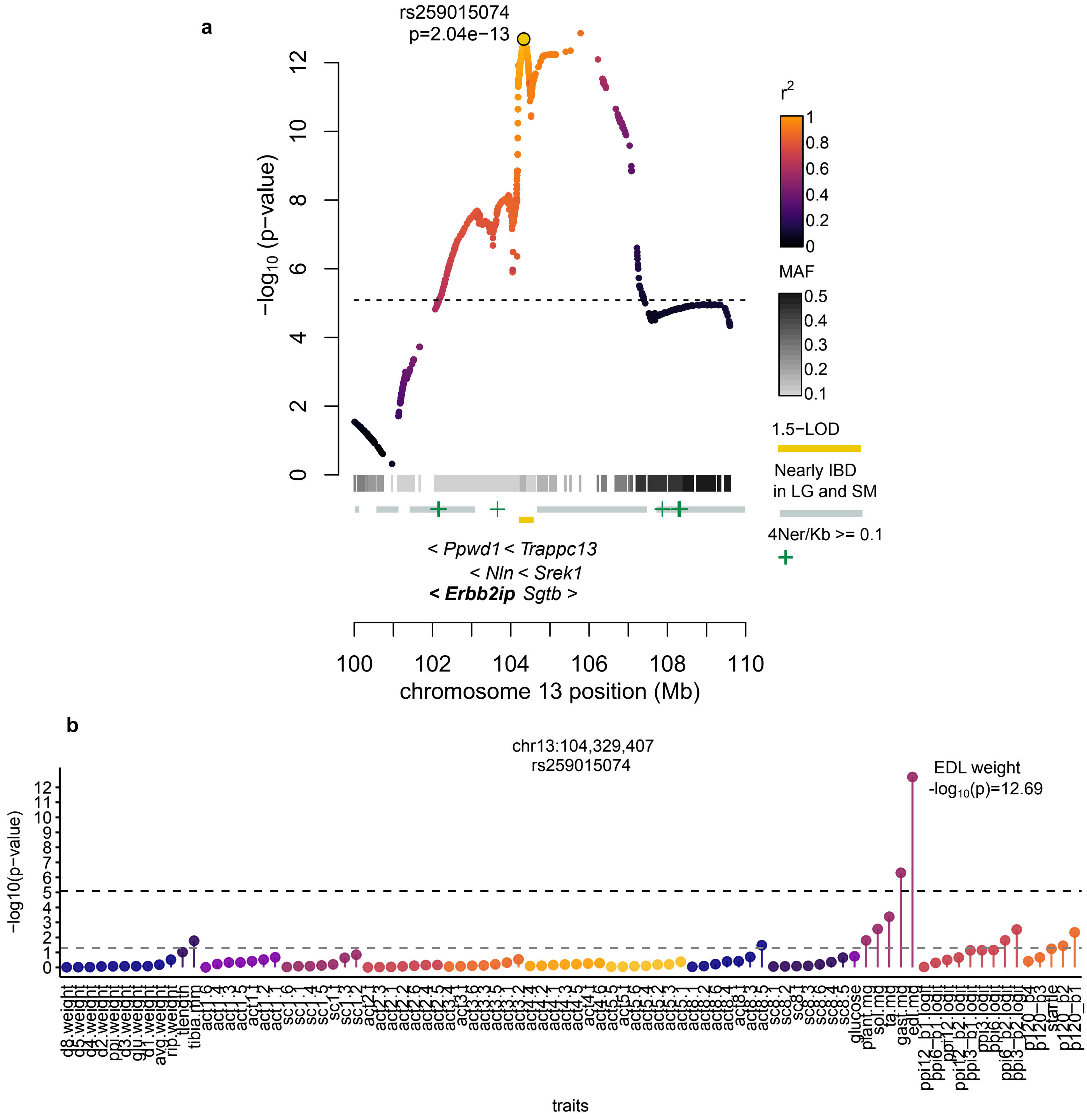

Figure 15: **A strong association with EDL mass on chromosome 13. (a)** Regional association plot of the association between rs259015074 (gold dot) and EDL mass, drawn from the set of 4.3M SNPs imputed from LG and SM. The location of *cis*-eQTL genes, 1.5-LOD interval (gold bar), areas of elevated recombination from Brunschwig *et al.* (plus symbols), regions predicted by Nikolskiy *et al.* to be nearly IBD between LG and SM (grey bars), and SNP MAFs (grey heat map) are indicated. Points are colored by LD (r^2) with rs259015074. The dashed line indicates a significance threshold of $-log_{10}(p) = 5.09 (\alpha = 0.05)$. eQTL genes containing missense mutations (dbSNP v.142) are highlighted in bold. **(b)** PheWAS plot showing an association on chromosome 13 between rs259015074 and other traits measured in this study. Dashed lines mark the genome-wide significance threshold as in **(a)** and a nominal significance level of p=0.05. Traits are listed by ID, grouped by type and sorted in ascending order of association with rs259015074 (full trait descriptions are provided in **Supplementary Data 2**).

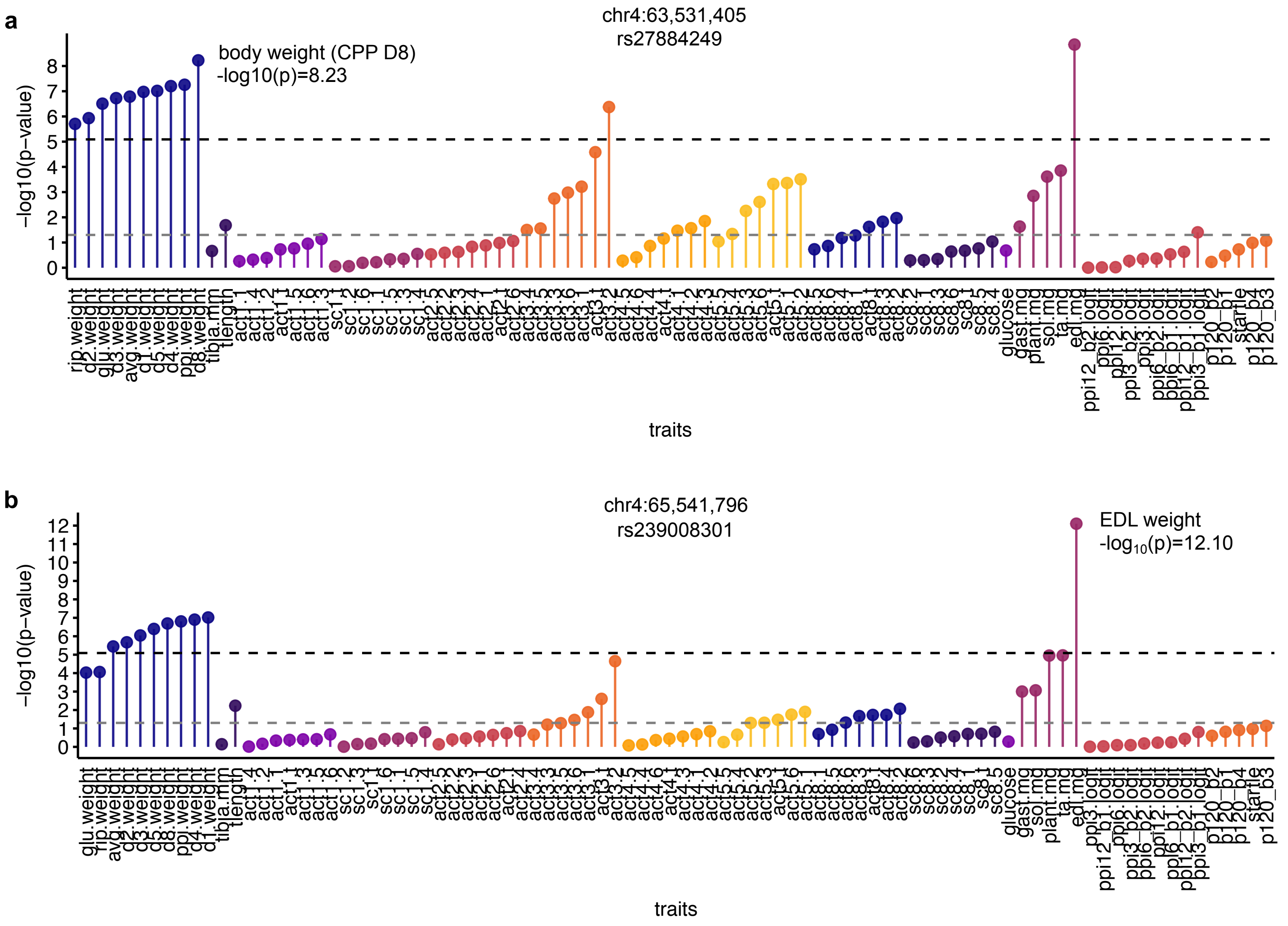

Figure 16: **Pleiotropic effects on physiology and behavior at a locus on chromosome 4. (a)** PheWAS plot of an association on chromosome 4 between rs27884249 (the top SNP for body weight on D8 of the CPP test at this locus) and other traits measured in this study. **(b)** PheWAS plot of an association between rs239008301 (the top SNP for EDL mass at this locus) and other traits measured in this study. rs27884249 and rs239008301 are located 2.01 Mb apart; LD (r^2) between the two SNPs is 0.663. Dashed lines mark the genome-wide significance threshold of $-log_{10}(p) = 5.09(\alpha = 0.05)$ and a nominal significance level of $p = 0.05$. Traits are listed by ID, grouped by type and sorted in ascending order of association with the top SNP (full trait descriptions are provided in **Supplementary Data 2**).

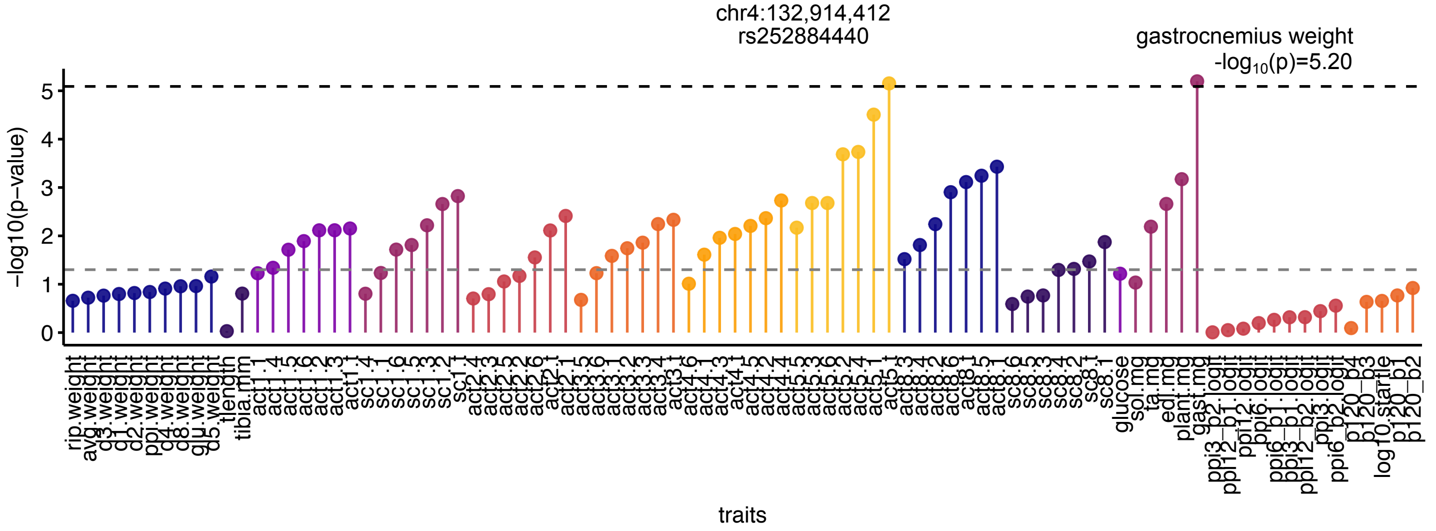

Figure 17: **Pleiotropic effects on gastrocnemius weight and locomotor activity at a locus on chromosome 4.** PheWAS plot of an association on chromosome 4 between rs252884440 (the top SNP for gastrocnemius muscle weight at this locus) and other traits measured in this study. Dashed lines mark the genome-wide significance threshold of $-log_{10}(p) = 5.09(\alpha = 0.05)$ and a nominal significance level of $p = 0.05$. Traits are listed by ID, grouped by type and sorted in ascending order of association with the top SNP (full trait descriptions are provided in **Supplementary Data 2**).

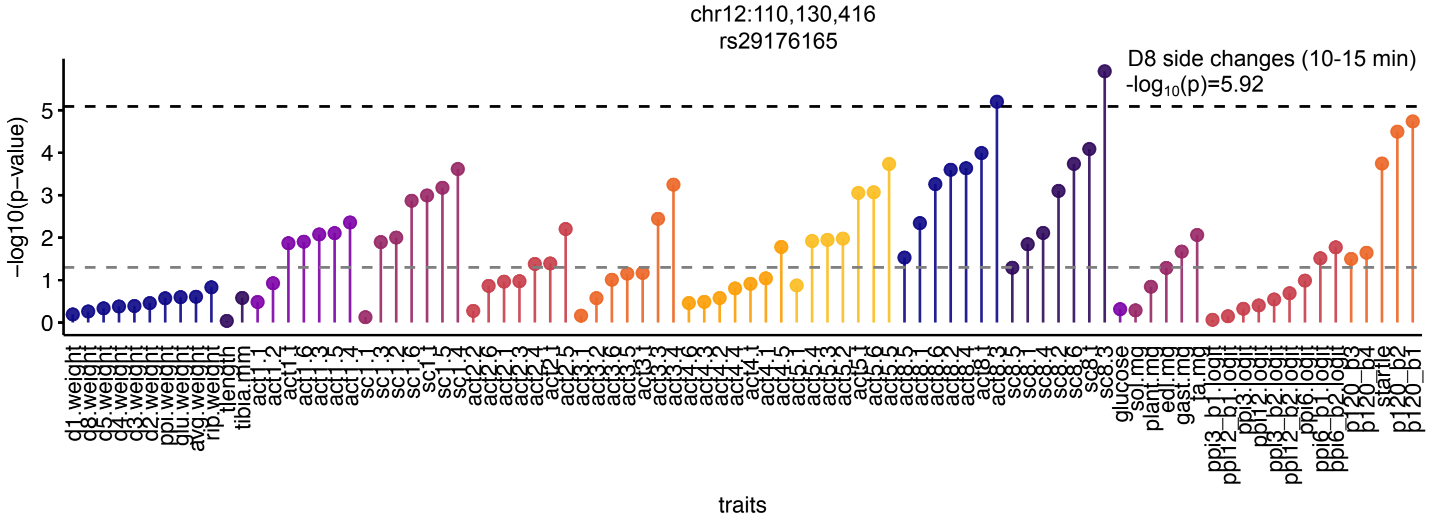

Figure 18: **Pleiotropic effects of a locus associated with locomotor activity on chromosome 12.** PheWAS plot of an association on chromosome 12 between rs29176165 and other traits measured in this study. Dashed lines mark the genome-wide significance threshold of $-log_{10}(p) = 5.09(\alpha = 0.05)$ and a nominal significance level of $p = 0.05$. Traits are listed by ID, grouped by type and sorted in ascending order of association with the top SNP (full trait descriptions are provided in **Supplementary Data 2**).

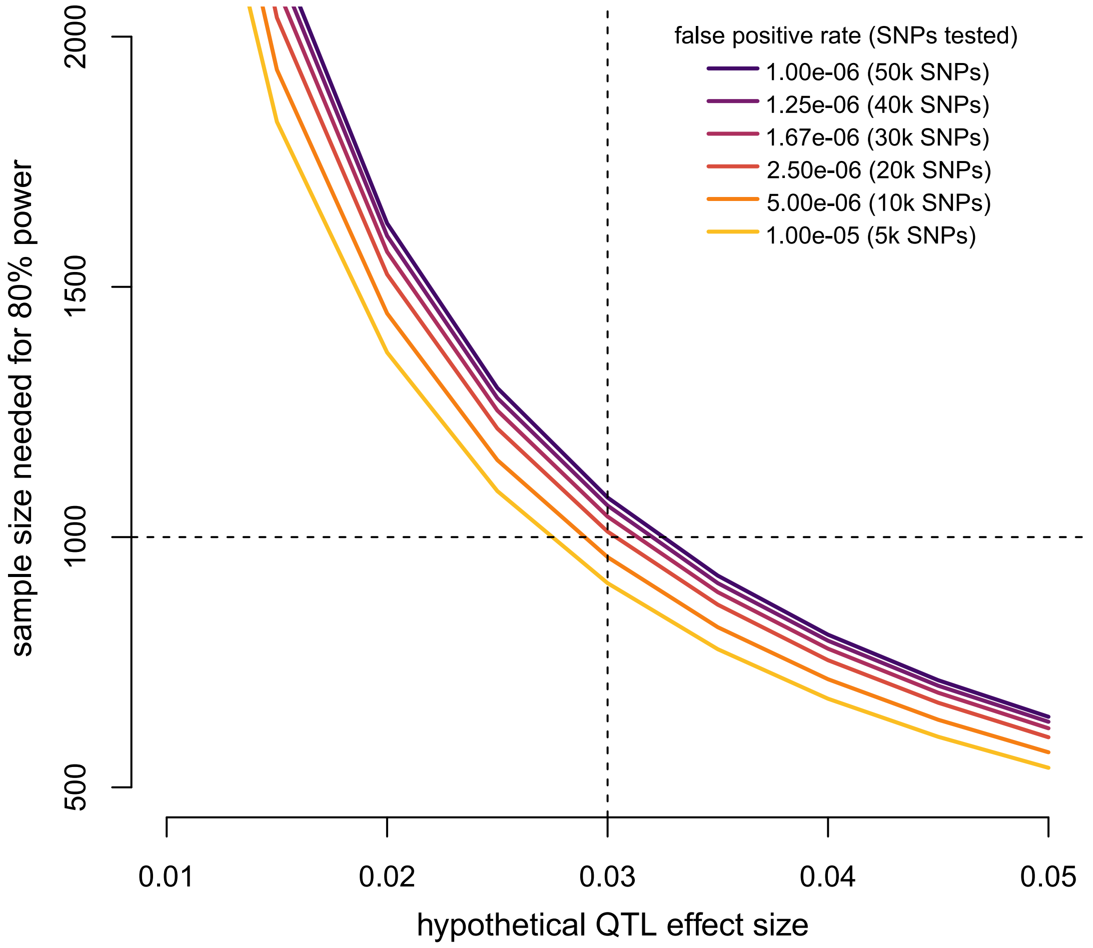

Figure 19: **Sample size needed for 80% power to detect associations with effect sizes ranging from 0.01 to 0.05.** Each line represents the Bonferroni-corrected false positive rate ($\alpha = 0.05$) for a hypothetical data set containing between 5,000 and 50,000 independent SNPs. For example, a sample of 1,000 mice would be needed to detect associations with an effect size of 0.03 given a Bonferroni-corrected false positive rate of 1.0×10^{-06} (or, equivalently, given 50,000 independent association tests). Calculations are based on a simple linear model that does not account for relatedness or non-additive genetic effects.

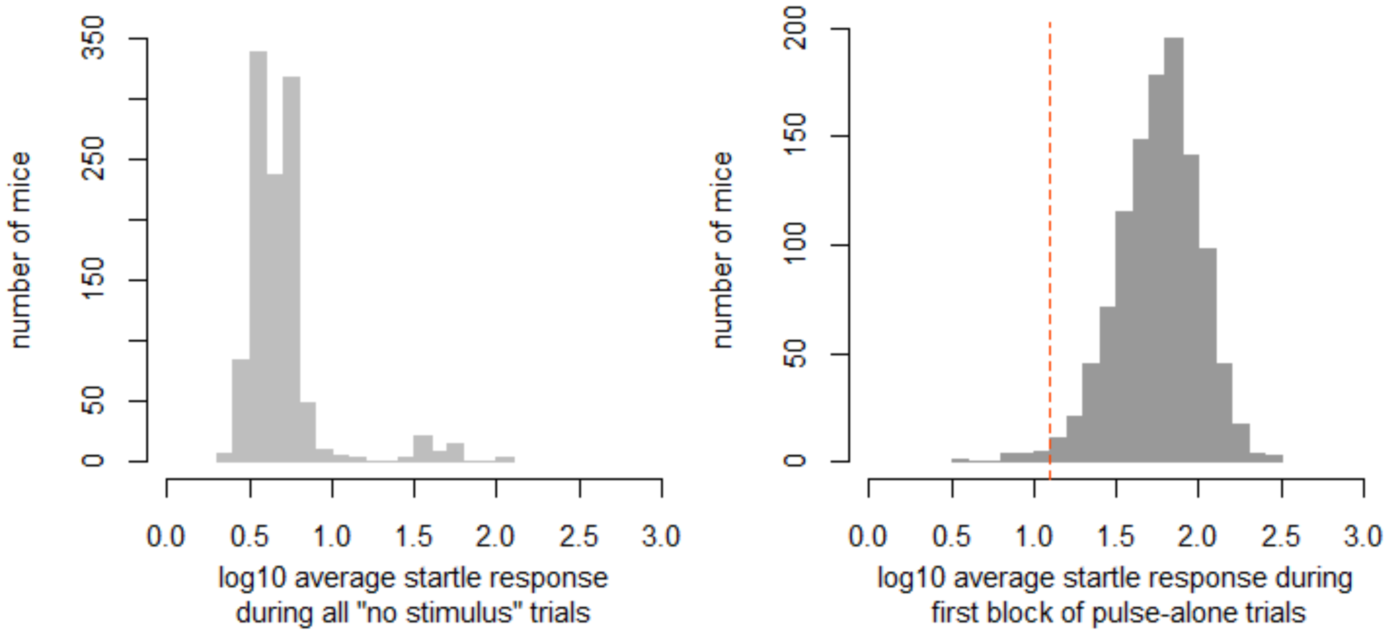

Figure 20: **Identification of outliers for startle and PPI. (a)** Distribution of the mean response measured across eight trials when no startle stimulus was presented. The right tail includes 44 mice, all of whom were tested in box 3, that appear to startle in the absence of a startle stimulus. We interpreted this as a technical effect and included box 3 as a covariate for all PPI and startle traits. **(b)** Distribution of the mean startle response during the first block of pulse-alone trials. Mice falling within the tail of the startle response distribution are not responding to the startle stimuli, possibly due to a hearing impairment. We excluded PPI and startle measures for 13 mice whose mean startle response overlapped the distribution of no-stimulus trials (not including mice from box 3). The cutoff point of 1.1 is marked with a dashed line. Each panel includes data for 1,123 phenotyped mice.

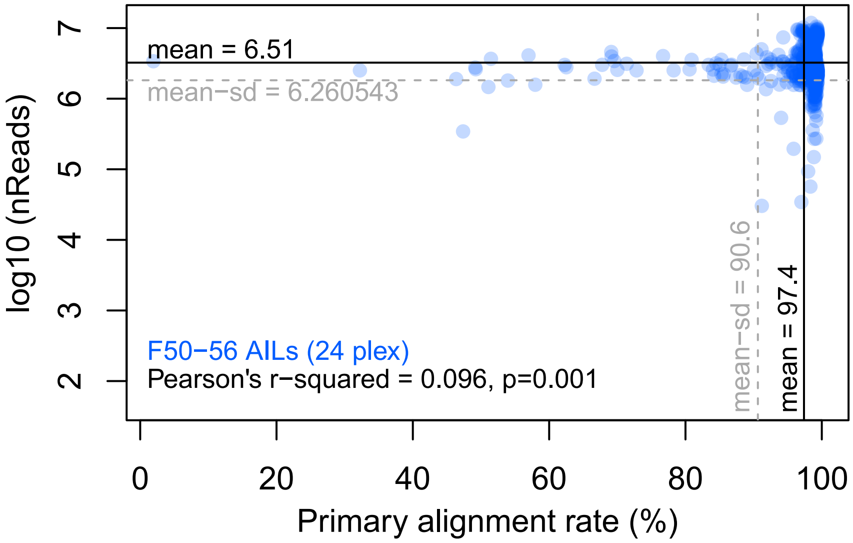

Figure 21: **Primary alignment rate and number of reads in GBS samples.** Mean alignment rate and mean log_{10}-transformed number of reads for all 1,100 GBS samples are shown as solid lines; their standard deviations are shown as dashed lines. We sequenced 24 samples per lane on an Illumina HiSeq 2500 (Illumina, San Diego, USA) using 100 bp SE reads. We discarded 32 samples with $<$1M reads aligned to the main chromosome contigs (1-19, X, Y) or with a primary alignment rate $<$77% (3 s.d. Lower than the mean). Data for the remaining 1,078 samples are plotted as blue points.

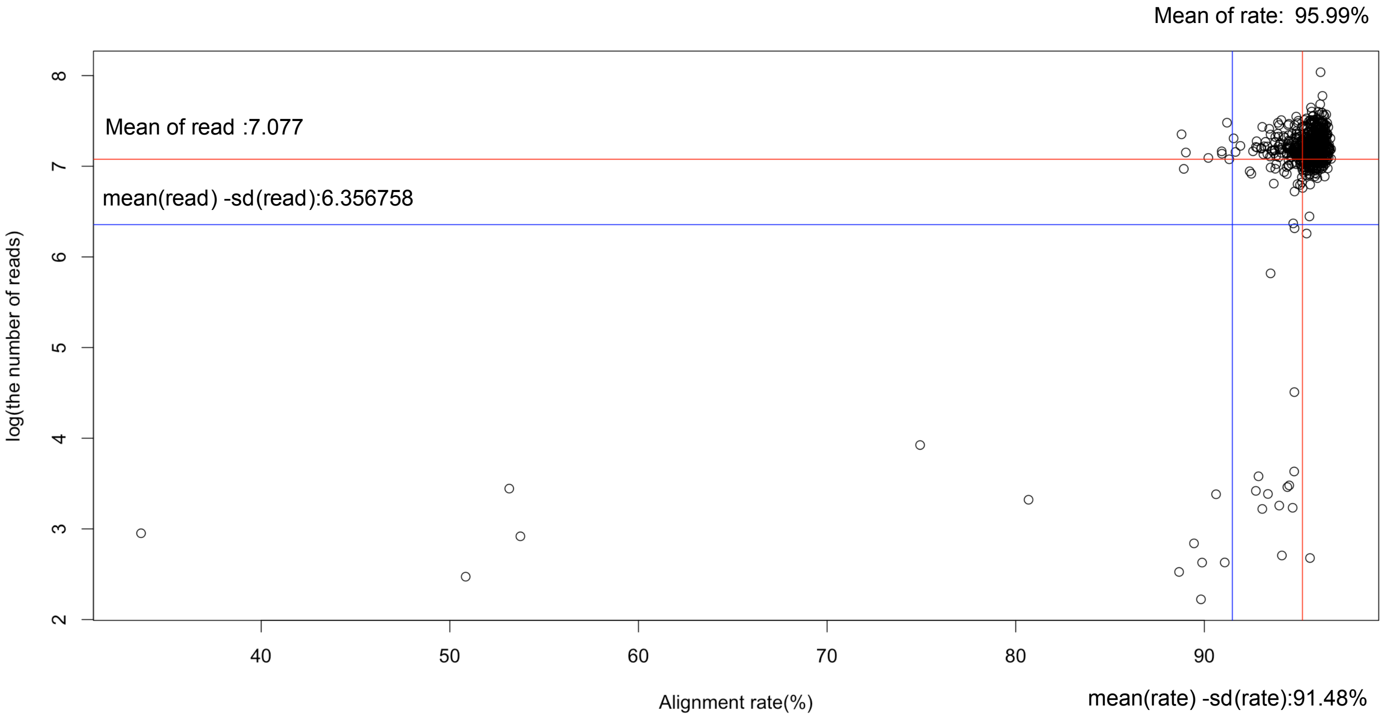

Figure 22: **Primary alignment rate and number of reads in RNA-seq samples.** Samples with an alignment rate less than 91.48% (blue vertical line) or fewer than 5M reads (blue horizontal line; $log_{10} - transformedvalue = 6.698$) were removed.

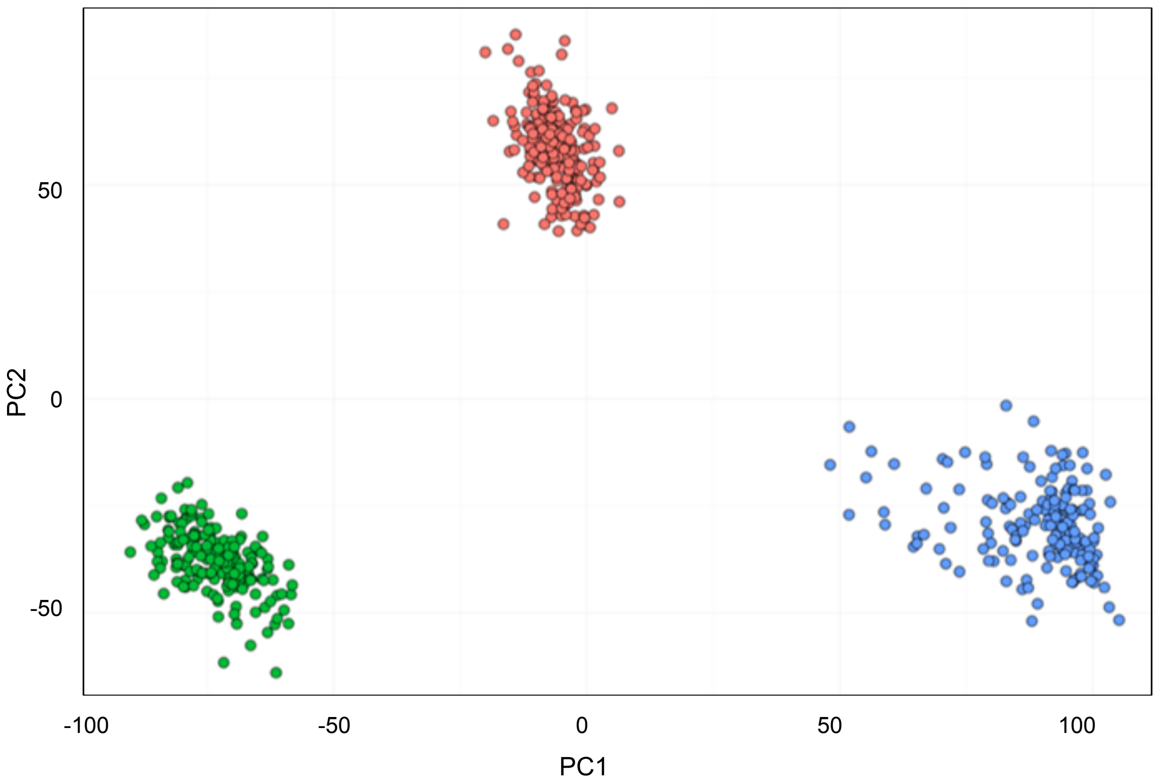

Figure 23: **Principal components analysis of RNA-seq data after correcting sample mix-ups.** The first two PCs cluster RNA-seq samples into the correct tissue types, indicating that we assigned mixed-up samples to the correct tissue. HIP is shown in pink, PFC in green, and STR in blue.

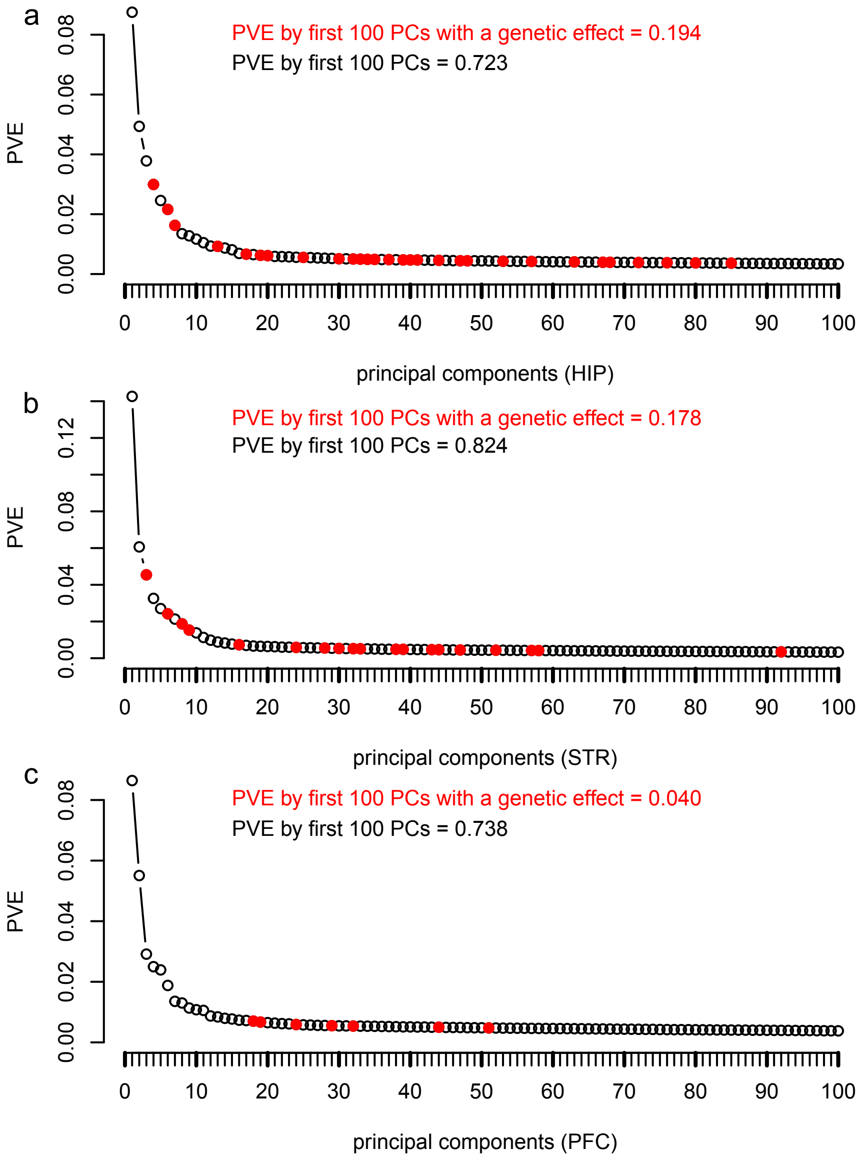

Figure 24: **Principal components of gene expression data for HIP, STR and PFC.** Scree plots showing the percent variance explained (PVE) by the first 100 principal components (PCs) of gene expression data for HIP **(a)**, STR **(b)**, and PFC **(c)**. We performed GWAS in each tissue using the first 100 PCs as phenotypes. PCs that showed evidence of association with a SNP ($-log_{10}(p) = 5.09; \alpha = 0.05$) were retained; these are shown as solid red dots. In total, 71 PCs were removed from HIP data, 81 PCs were removed from STR data, and 93 PCs were removed from PFC data.
