# Supplementary material for: Genome wide association analysis in a mouse advanced intercross line
Source: Nat Commun. 2018 Dec 4;9:5162. doi: 10.1038/s41467-018-07642-8 (PMC6279738; doi:10.1038/s41467-018-07642-8)
Supplement: Supplementary file 3 — Description of Additional Supplementary Files [file 41467_2018_7642_MOESM3_ESM.pdf]

Legends for Supplementary Data 1-6

Supplementary Data 1: GWAS results. This table summarizes 124 genome-wide significant associations identified in this study. Most information pertains to the most significantly associated SNP in each region, including: p-values, rsids, MAFs and alleles. We have also provided raw trait means and quantile-normalized residual trait means stratified by genotype at the most significant SNP at each trait-associated locus (covariates in Supplementary Data 2 were regressed out to produce the residual values) Finally, we list the number of genes, *cis*-eQTL genes and genes containing potentially deleterious mutations (missense, stop-gain, or stop-loss as annotated by dbSNP v.142) that fall within each locus. A gene was counted as being within the locus if its transcription start-end coordinates overlapped the region. A *cis*-eQTL gene was counted if any part of the gene body or its 1 Mb *cis* region fell inside the locus.

Supplementary Data 2: Traits, heritabilities, and covariates. Information about the traits measured in G50-56 mice is provided, including trait descriptions, raw trait means and standard deviations, GWAS sample size, covariates, and heritability estimates with standard error. Estimates of SNP heritability and pedigree-based heritability from GEMMA are provided.

Supplementary Data 3: Trait correlations. Pearson's correlation coefficients (including p-values and FDR), genetic variance components (V_g) and their standard errors, and environmental variance components (V_e) and their standard errors are reported for each pair of traits (full trait names and descriptions are provided in Supplementary Data 2).

Supplementary Data 4: *cis*-eQTLs. *cis*-eQTLs (FDR<0.05) and gene information is provided, including the physical position of *cis*-eQTL SNPs, raw eQTL p-values, eigenMT-adjusted eQTL p-values, the number of *cis* SNPs tested and the effective number of independent tests, FDR, tissue, gene coordinates, *cis*-region coordinates, strand, and gene width.

Supplementary Data 5: *trans*-eQTLs. This table reports *trans*-eQTLs (α=0.05) and target gene information, including position, tissue, rsid and dbSNP (v.142) annotations for each eQTL SNP, eQTL p-values, transcription start and end positions for the target gene, and gene annotation downloaded from the Mouse Genome Informatics database.

Supplementary Data 6: AIL pedigree. Text file containing the LG x SM AIL pedigree from generation 0 (the initial cross between LG/J and SM/J) to generation 56. Columns include mouse ID, sire ID, dam ID, sex (1=M; 2=F), and generation. We inherited the LG x SM AIL from Dr. James Cheverud in generation 33. We added 0.1 to all mouse ID numbers in all subsequent generations to prevent overlap between ear tag ID numbers used by our lab and ear tag IDs used by the Cheverud lab.
